# Low membrane fluidity triggers lipid phase separation and protein segregation in living bacteria

**DOI:** 10.15252/embj.2021109800

**Published:** 2022-01-17

**Authors:** Marvin Gohrbandt, André Lipski, James W Grimshaw, Jessica A Buttress, Zunera Baig, Brigitte Herkenhoff, Stefan Walter, Rainer Kurre, Gabriele Deckers‐Hebestreit, Henrik Strahl

**Affiliations:** ^1^ Mikrobiologie, Fachbereich Biologie/Chemie Universität Osnabrück Osnabrück Germany; ^2^ Lebensmittelmikrobiologie und ‐hygiene Institut für Ernährungs‐ und Lebensmittelwissenschaften Rheinische Friedrich‐Wilhelms‐Universität Bonn Bonn Germany; ^3^ Centre for Bacterial Cell Biology Biosciences Institute Faculty of Medical Sciences Newcastle University Newcastle upon Tyne UK; ^4^ Center of Cellular Nanoanalytics Integrated Bioimaging Facility Universität Osnabrück Osnabrück Germany

**Keywords:** homeoviscous adaptation, lipid domains, lipid phase separation, membrane fluidity, protein partitioning, Membranes & Trafficking, Microbiology, Virology & Host Pathogen Interaction

## Abstract

All living organisms adapt their membrane lipid composition in response to changes in their environment or diet. These conserved membrane‐adaptive processes have been studied extensively. However, key concepts of membrane biology linked to regulation of lipid composition including homeoviscous adaptation maintaining stable levels of membrane fluidity, and gel‐fluid phase separation resulting in domain formation, heavily rely upon *in vitro* studies with model membranes or lipid extracts. Using the bacterial model organisms *Escherichia coli* and *Bacillus subtilis*, we now show that inadequate *in vivo* membrane fluidity interferes with essential complex cellular processes including cytokinesis, envelope expansion, chromosome replication/segregation and maintenance of membrane potential. Furthermore, we demonstrate that very low membrane fluidity is indeed capable of triggering large‐scale lipid phase separation and protein segregation in intact, protein‐crowded membranes of living cells; a process that coincides with the minimal level of fluidity capable of supporting growth. Importantly, the *in vivo* lipid phase separation is not associated with a breakdown of the membrane diffusion barrier function, thus explaining why the phase separation process induced by low fluidity is biologically reversible.

## Graphical abstract

Essential cellular processes in bacteria, including cytokinesis, envelope expansion, chromosome replication/segregation and maintenance of membrane potential, are impaired by low membrane fluidity.

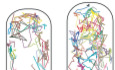

## Introduction

Biological membranes are complex arrangements predominantly composed of lipids and both integral and surface‐attached proteins (Nicolson, 2014). The primordial function of biological membranes was likely to act as a simple, semipermeable diffusion barrier separating the cell from its environment, and genomes from each other (Chen & Valde, 2010). Later, membranes and membrane proteins evolved to fulfil a multitude of cellular functions including transport, respiration and morphogenesis. Since the physicochemical state of biological membranes is highly sensitive to changes in the environment including temperature, osmolarity, salinity, pH or diet (Razin, 1967; Hazel, 1995; Ernst *et al*, 2016), careful homeostatic regulation of key membrane parameters such as thickness or fluidity is vital for cell function (Parsons & Rock, 2013; Ernst *et al*, 2016; Harayama & Riezman, 2018; Levental *et al*, 2020).

Arguably, the best studied membrane‐adaptive process is homeoviscous adaptation that acts upon changes in temperature (Hazel, 1995; Parsons & Rock, 2013; Ernst *et al*, 2016). With increasing temperature, lipid bilayers exhibit reduced head group packing, increased fatty acid disorder and increased fluidity in terms of increased rotational and lateral diffusion of molecules (Chapman, 1975; Heimburg, 2007). While all of these membrane parameters are closely interconnected, membrane fluidity is thought to be the key property actively maintained at stable levels that optimally support vital membrane functions through homeoviscous adaptation processes (Hazel, 1995; Parsons & Rock, 2013; Ernst *et al*, 2016). All living organisms achieve this by actively adapting their lipid composition. Most commonly, this is obtained by altering the content of lipids carrying fluidity‐promoting unsaturated fatty acids (UFA) or branched chain fatty acids (BCFA) and fluidity‐reducing saturated fatty acids (SFA), respectively, thereby counteracting shifts in membrane fluidity (Hazel, 1995; Diomandé *et al*, 2015; Ernst *et al*, 2016).

While adaptive changes in lipid fatty acid composition as well as the regulatory processes involved are increasingly well characterised (Mansilla *et al*, 2004; Ernst *et al*, 2018; Ballweg *et al*, 2020), the cellular consequences of inadequate membrane fluidity are significantly less understood. Sufficiently high membrane fluidity has been implicated in promoting folding, catalytic activity and diffusion of membrane proteins (Lee, 2004; Andersen & Koeppe, 2007). Too high membrane fluidity, in turn, has been shown to increase proton permeability *in vitro* (Rossignol *et al*, 1982; van de Vossenberg *et al*, 1999), thus potentially hampering with efficient ion homeostasis and energy conservation (Valentine, 2007), while too low membrane fluidity impedes respiration due to reduced ubiquinone diffusivity (Budin *et al*, 2018). However, our understanding of the behaviour of biological membranes upon changing fluidity is predominantly based on *in vitro* and *in silico* studies with simplified model lipids, or *in vitro* studies with either natural lipid extracts or isolated membranes (Baumgart *et al*, 2007; Schäfer *et al*, 2011; Nickels *et al*, 2017).

One of the fascinating properties of lipids is their ability to undergo phase transitions between distinct configurations that differ in terms of ability to form bilayers, membrane thickness and degree of lipid packing (Chapman, 1975). The biologically relevant bilayer‐forming lipid phases are: (i) the liquid‐disordered phase characterised by low packing density and high diffusion rates that forms the regular state of biological membranes, (ii) the cholesterol/hopanoid‐dependent liquid‐ordered phase that forms nanodomains (lipid rafts) found in biological membranes, both representing different fluid phases; and (iii) the gel or solid phase characterised by dense lipid packing with little lateral or rotational diffusion, which is generally assumed to be absent in biologically active membranes (Veatch, 2007; Sáenz *et al*, 2015; Schmid, 2017). In fact, the temperature associated with gel phase formation has been postulated to define the lower end of the temperature range able to support vital cell functions (Drobnis *et al*, 1993; Ghetler *et al*, 2005; Burns *et al*, 2017), and maintaining biological membranes in the correct phase (homeophasic regulation) has been suggested as an alternative rationale behind temperature‐dependent lipid adaptation (Hazel, 1995). Finally, lipid phases can co‐exist, resulting in separated membrane areas exhibiting distinctly different composition and characteristics (Baumgart *et al*, 2007; Elson *et al*, 2010; Heberle & Feigenson, 2011; Nickels *et al*, 2017; Shen *et al*, 2017). This principal mechanism of lipid domain formation is best studied in the context of lipid rafts (Lingwood & Simons, 2010; Shaw *et al*, 2021). Here, the co‐existence of fluid liquid‐disordered and liquid‐ordered phases, and the associated protein segregation, has been demonstrated in membranes of living eukaryotic cells (Toulmay & Prinz, 2013; preprint: Shelby *et al*, 2021). In contrast, comprehensive *in vivo* studies on gel‐fluid lipid phase separation in live cells have been challenging due to the tendency of cholesterol/hopanoids to suppress gel‐fluid phase transitions (Heberle & Feigenson, 2011), and due to the difficulty to modify the membrane fatty acid composition and, thus, fluidity without inducing lipotoxicity (Shen *et al*, 2017; Budin *et al*, 2018).

While *in vitro* and *in silico* approaches with simplified lipid models have provided detailed insights into the complex physicochemical behaviour of lipid bilayers, testing the formed hypotheses and models in the context of protein‐rich biological membranes is now crucial. Bacteria tolerate surprisingly drastic changes in their lipid composition and only possess one or two membrane layers as part of their cell envelope. Consequently, bacteria are both a suitable and a more tractable model to study the fundamental biological process linked to membrane fluidity and phase separation *in vivo*.

We analysed the biological importance of membrane homeoviscous adaptation in *Escherichia coli* (phylum *Proteobacteria*) and *Bacillus subtilis* (phylum *Firmicutes*), respectively. These organisms were chosen due to their prominence as Gram‐negative and Gram‐positive model organisms, and the different archetypes of membrane fatty acid composition (straight versus branched chain fatty acids) they represent. We have established protocols that allow the fatty acid composition of both organisms to be progressively altered and the cellular consequences to be directly monitored in growing cells. This approach allowed us to address three central questions linked to homeostatic regulation of membrane composition and fluidity: (i) what are the cellular consequences of an inadequate level of membrane fluidity that necessitate the extensive and conserved homeostatic regulatory processes, (ii) how do changes in lipid fatty acid composition translate to changes in membrane fluidity of living cells and (iii) what is the lipid phase behaviour in living cells with protein‐crowded membranes and intact lipid domain organisation?

Our results demonstrate that too low membrane fluidity results in growth arrest in both organisms, which is accompanied by severe disturbances of the cell morphogenesis and ion homeostasis. Furthermore, too low fluidity triggers a striking, large‐scale lipid phase separation into liquid‐disordered and gel phase membranes, accompanied by segregation of otherwise disperse membrane proteins such as ATP synthase and glucose permease. Our results revealed that phase separation between liquid‐disordered and gel state membranes is associated with loss of essential membrane functions, thereby limiting the range of membrane fluidity able to support life. At last, our findings demonstrating that gel‐liquid phase separation and associated membrane protein segregation indeed occurs in protein‐crowded, native plasma membranes of living cells, are fully consistent with the comparable phenomena observed in *in vitro* and *in silico* model systems (Baumgart *et al*, 2007; Veatch, 2007; Lingwood & Simons, 2010; Schäfer *et al*, 2011; Domański *et al*, 2012). Thus, the results provide strong *in vivo* support for the general validity of the respective models.

## Results

### Depletion of BCFAs in *B. subtilis*

To modify the fatty acid composition in *B. subtilis*, we constructed a Δ*bkd* Δ*des* deletion strain (Appendix Table S1). The *bkd* operon encodes enzymes catalysing the conversion of branched chain amino acids into intermediates for BCFA synthesis (Debarbouille *et al*, 1999). The lack of this activity can be complemented by supplementation with precursors 2‐methylbutyric acid (MB) or isobutyric acid (IB) (Kaneda, 1977; Boudreaux *et al*, 1981). This provides the experimental means to control the lipid *iso*‐ and *anteiso*‐BCFA composition (Appendix Fig S1A) normally responsible for the homeostatic adaptation of membrane fluidity in response to environmental changes (Diomandé *et al*, 2015). In addition, the strain is deficient for the lipid desaturase Des to prevent rapid adaptation of membrane fluidity by converting SFAs or BCFAs into UFAs (Diomandé *et al*, 2015). In the remaining text, the *B. subtilis* strain is labelled “Δ*bkd*” for simplicity.

**Figure 1:**
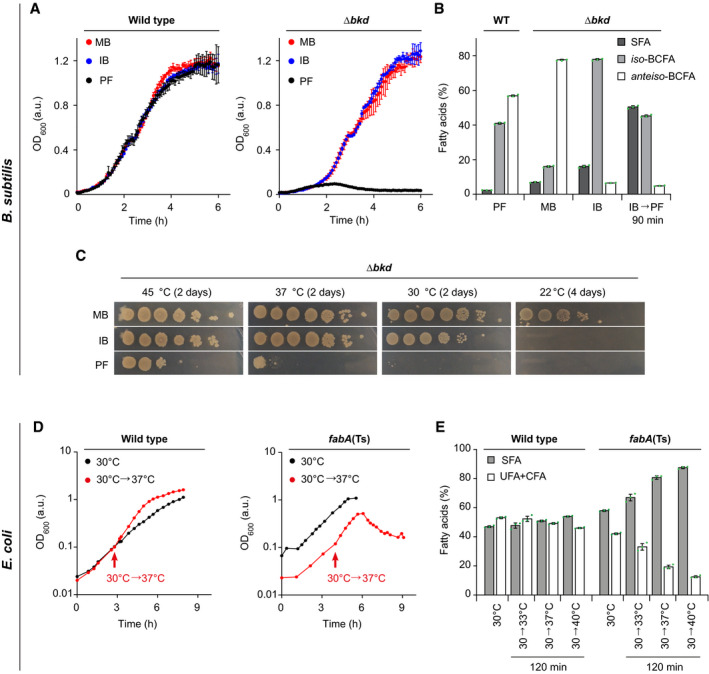
Membrane fatty acid composition‐dependent growth of *B. subtilis* and *E. coli* Growth of *B. subtilis* WT and fatty acid precursor‐auxotrophic Δ*bkd* cells in medium supplemented with precursor 2‐methyl butyric acid (MB), isobutyric acid (IB) or grown precursor‐free (PF).Fatty acid composition of *B. subtilis* WT cells grown in PF medium, and Δ*bkd* grown with MB, IB or depleted for precursor for 90 min (IB→PF). For detailed analyses, see Appendix Fig S1B.Temperature‐dependent growth of *B. subtilis* Δ*bkd* on solid medium in serial 10‐fold dilutions. For comparison between WT, Δ*des* and Δ*bkd* Δ*des* cells, see Appendix Fig S1C.Temperature‐dependent growth behaviour of *E. coli* WT and *fabA*(Ts), including a shift from 30 to 37°C as non‐permissive temperature of *fabA*(Ts).Fatty acid composition of *E. coli* WT and *fabA*(Ts) cells grown at 30°C and shifted to different temperatures for 120 min. For detailed analyses, see Appendix Fig S2B and C. Data information: (A) The diagram depicts mean and standard deviation (SD) of technical triplicates for each strain. (B, E) The histograms depict mean and SD of biological triplicates. (C, D) The experiments are representative of three independent repeats. CFA, cyclopropane fatty acid. Strains used: (A–C) *B. subtilis* 168, HS527; (D, E) *E. coli* MG1, MG4 (strains Y‐Mel and UC1098, respectively, additionally encoding fluorescent ATP synthase (F_O_F_1_ *a*‐mNG)). Source data are available online for this figure.

We compared growth of *B. subtilis* 168 used as wild‐type (WT) and Δ*bkd* cells at 37°C upon supplementation with BCFA precursors MB or IB (Fig 1A). While BCFA precursors had little impact on growth of WT cells, the auxotrophic Δ*bkd* strain only grew in the presence of either of the precursors. Corresponding fatty acid analyses revealed large shifts in the composition of the Δ*bkd* strain depending on the supplied precursor (Fig 1B and Appendix Fig S1B). As expected, cells supplemented with MB exhibited a high content (77%) of *anteiso*‐BCFAs, whereas cells grown with IB showed a high content of *iso‐*BCFAs (77%). To obtain cells depleted for both BCFA types, cells were grown in the presence of IB, followed by wash and incubation in precursor‐free (PF) medium. This precursor depletion leads to growth arrest after about 90 min (Appendix Fig S1B), corresponding to an accumulated SFA content of ~50% (Fig 1B).

Analyses of Δ*bkd* cells grown at different growth temperatures with different BCFA precursors (Fig 1C and Appendix Fig S1C) indicated that only MB, the precursor for *anteiso‐*BCFAs, is capable for supporting robust growth at low temperatures (22°C). At 30°C and 37°C, growth was comparable in the presence of either MB or IB, while no growth was observed in the absence of precursors, demonstrating that a high BCFA level is essential for growth under these conditions. At 45°C, Δ*bkd* could grow at low dilutions even in the absence of BCFA precursors (Fig 1C and Appendix Fig S1C). For these reasons, we chose growth with IB at 37°C, as the reference condition for *B. subtilis* Δ*bkd*.

### Depletion of UFAs in *E. coli*

In *E. coli*, membrane fluidity is modulated by UFAs (Parsons & Rock, 2013) (Appendix Fig S2A). While synthesis of UFAs is essential for *E. coli* growth, a temperature‐sensitive *fabF fabA*(Ts) mutant (Appendix Table S1), in which a shift to non‐permissive growth temperatures leads to UFA depletion, has been isolated (Cronan & Gelmann, 1973). DNA sequencing of *fabF* and *fabA* from this rather old isolate revealed that FabF (β‐ketoacyl‐ACP synthase II) is non‐functional due to S291N and G262S substitutions. FabA (β‐hydroxyacyl‐ACP‐dehydratase/isomerase) carries a single G101D substitution (Rock *et al*, 1996). Based on the structure of the FabA head‐to‐tail homodimer (Nguyen *et al*, 2014), G101 is positioned at the border of the dimerisation interface. Consequently, the G101D substitution could plausibly cause thermosensitivity by destabilising the essential dimer structure of FabA at elevated temperatures, thereby provoking the gradual depletion of UFA (Cronan & Gelmann, 1973). Thus, this strain provides the experimental tool to control the UFA/SFA balance in growing cells. Throughout the text, the strain is labelled “*fabA*(Ts)” for simplicity.

While the growth of *fabA*(Ts) at 30°C is comparable to *E. coli* Y‐Mel used as WT, transfer to non‐permissive temperatures such as 37°C only supported growth for about 120 min, followed by growth arrest and onset of cell lysis (Fig 1D). Corresponding fatty acid analyses confirmed a strong, temperature‐dependent decrease in UFAs (Fig 1E and Appendix Fig S2). In agreement with Cronan and Gelmann (1973), a minimal amount of 10–15% UFAs appeared to be essential to support growth (Fig 1E and Appendix Fig S2B). In comparison, WT cells showed only minor, temperature‐dependent changes in fatty acid composition caused by homeoviscous adaptation towards increased SFA content at higher temperatures (Fig 1E and Appendix Fig S2C).

### Reduced membrane fluidity in cells depleted of UFAs or BCFAs

**Figure 2:**
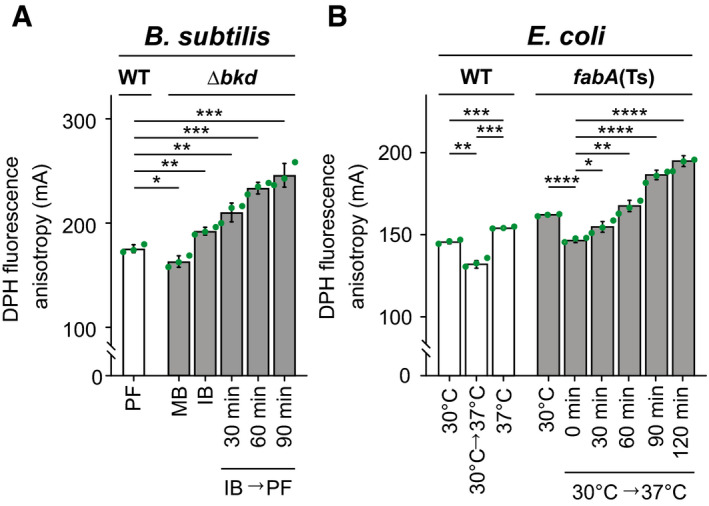
Reduced membrane fluidity in cells depleted for UFAs or BCFAs DPH anisotropy of *B. subtilis* WT or Δ*bkd* cells supplemented either with MB, IB or depleted for precursor (IB→PF) for the times indicated. High DPH anisotropy indicates low membrane fluidity.DPH anisotropy of *E. coli* WT cells grown steady state at 30, 37°C or shifted from 30 to 37°C followed by immediate measurement. In addition, DPH anisotropy of *fabA*(Ts) cells grown steady state at 30°C or shifted from 30 to 37°C followed by measurement at the times indicated. Data information: (A, B) The experiments are representative of three independent repeats. The histograms depict means and SD of technical triplicates, together with *P* values of an unpaired, two‐sided *t*‐test. Significance was assumed with ****P < 0.0001, ***P < 0.001, **P < 0.01, *P < 0.05, n.s., not significant. Strains used: (A) *B. subtilis* 168, HS527; (B) *E. coli* Y‐Mel, UC1098. Source data are available online for this figure.

To confirm that changes in fatty acid composition translate to shifts in *in vivo* membrane fluidity, we monitored changes in steady‐state fluorescence anisotropy of 1,6‐diphenyl‐1,3,5‐hexatriene (DPH), the rotational freedom of which is sensitive to acyl chain disorder and, thus, indirectly to the fluidity of lipid bilayers (Lentz, 1993). DPH anisotropy measurements with *B. subtilis* Δ*bkd* revealed the highest membrane fluidity for cells with the highest *anteiso‐*BCFA content (Fig 2A). Cells with high *iso‐*BCFA content exhibited membrane fluidity levels slightly lower than those found for WT. These results confirm that *anteiso‐*BCFAs promote higher membrane fluidity than the corresponding *iso‐*forms *in vivo*; a difference previously based on *in vitro* evidence only (Lewis *et al*, 1987). The changes observed upon depletion of BCFAs, which is accompanied by accumulation of SFAs, were more drastic and resulted in a gradual reduction of membrane fluidity, ultimately leading to growth arrest (Fig 2A and Appendix Fig S1B).

DPH anisotropy measurements conducted with *E. coli* followed a similar trend (Fig 2B). Both *E. coli* WT and *fabA*(Ts) cells grown at 30°C exhibited an expected, immediate increase in membrane fluidity upon a shift to 37°C; a phenomenon that in WT cells is overtime counteracted by homeoviscous adaptation restoring membrane fluidity close to pre‐shift levels. However, in *fabA*(Ts) continued growth at 37°C resulted in a gradual increase in DPH anisotropy, thus confirming a substantial reduction of membrane fluidity.

In conclusion, the established fatty acid depletion procedures allow membrane fluidity to be controllably lowered to a point incapable of supporting growth in both organisms. In the following chapters, we use this approach to analyse which cellular processes are impaired by inadequate levels of membrane fluidity.

### Consequences of low membrane fluidity on membrane diffusion barrier function

The prevalence of adaptive mechanisms maintaining membrane fluidity (Hazel, 1995) might indicate its importance for preserving the fundamental membrane barrier function. To analyse the consequences of too low membrane fluidity on membrane leakiness, we used the combination of two fluorescent dyes. Sytox Green is a membrane‐impermeable, DNA‐intercalating dye used to assess the integrity of bacterial plasma membranes in terms of permeability (Roth *et al*, 1997). DiSC_3_(5), a voltage‐sensitive dye accumulating in cells with high membrane potential (te Winkel *et al*, 2016), indicates changes in either membrane ion conductivity or respiration.

**Figure 3:**
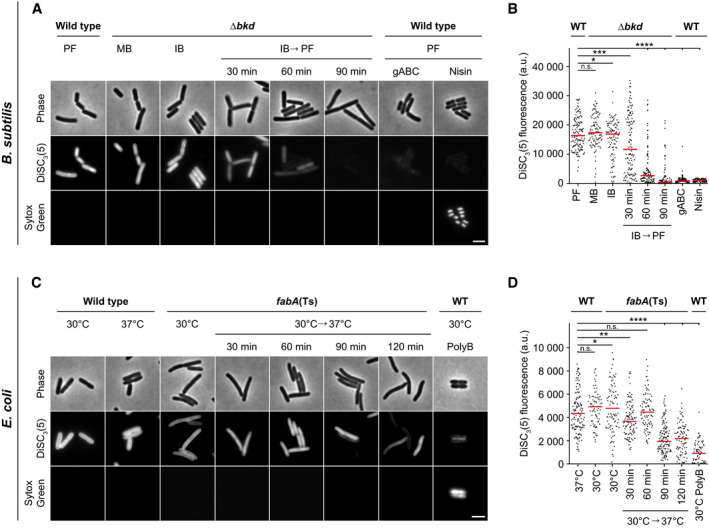
Consequences of low membrane fluidity on membrane diffusion barrier function Images of *B. subtilis* WT and Δ*bkd* cells co‐labelled with the membrane potential‐sensitive dye DISC_3_(5) and the membrane permeability indicator Sytox Green. Membrane properties were assessed for Δ*bkd* cells grown in the presence of MB, IB or washed precursor‐free (IB→PF) for the times indicated. As controls, WT cells were measured in the presence of depolarising antimicrobial peptide gramicidin ABC (gABC) or pore‐forming lantibiotic Nisin. For cross‐correlation between membrane depolarisation and membrane permeabilisation, see Appendix Fig S3A–C.Quantification of DISC_3_(5) fluorescence for cells (*n* = 100–142) depicted in panel A. Median represented by red line.Images of *E. coli* WT and *fabA*(Ts) cells co‐labelled with the same indicator dyes as in panel A. Membrane properties were assessed for *fabA*(Ts) at 30°C and upon transfer to non‐permissive 37°C for the times indicated. As controls, WT cells were incubated with the pore‐forming antibiotic Polymyxin B (PolyB). For cross‐correlation between membrane depolarisation and membrane permeabilisation, see Appendix Fig S3D and E. The integrity of the diffusion barrier function was additionally studied via ONPG permeability in a Δ*lacY* background (see Fig EV1).Quantification of DISC_3_(5) fluorescence for cells (*n* = 76–141) depicted in panel C. Median represented by red line. Data information: (A–D) The experiments are representative of three independent repeats. (B, D) Red lines indicate the median. *P* values represent the results of unpaired, two‐sided *t*‐tests. Significance was assumed with ****P < 0.0001, ***P < 0.001, **P < 0.01, *P < 0.05, n.s., not significant. (A, C) Scale bar, 3 µm. Strains used: (A, B) *B. subtilis* 168, HS527; (C, D) *E. coli* Y‐Mel, UC1098. Source data are available online for this figure.

Growing *B. subtilis* Δ*bkd* cells, irrespectively of the supplied BCFA precursor, exhibited DiSC_3_(5) fluorescence signals comparable to those observed for WT (Fig 3A and B, and Appendix Fig S3A–C). This indicates that the corresponding changes in the membrane fatty acid composition and fluidity had surprisingly little impact on membrane potential. In contrast, depletion of BCFAs triggered a gradual membrane depolarisation that was, in a mild form, already detectable after 30 min. A complete membrane depolarisation was observed after 90 min coinciding with growth arrest (Appendix Fig S1B). However, membranes remained impermeable for Sytox Green (Fig 3A), demonstrating that the gradual membrane depolarisation was not caused by a simple disruption of membrane continuity. In contrast, even the severely BCFA‐depleted membranes were fully capable of forming a continuous, tight diffusion barrier.

**Figure EV1:**
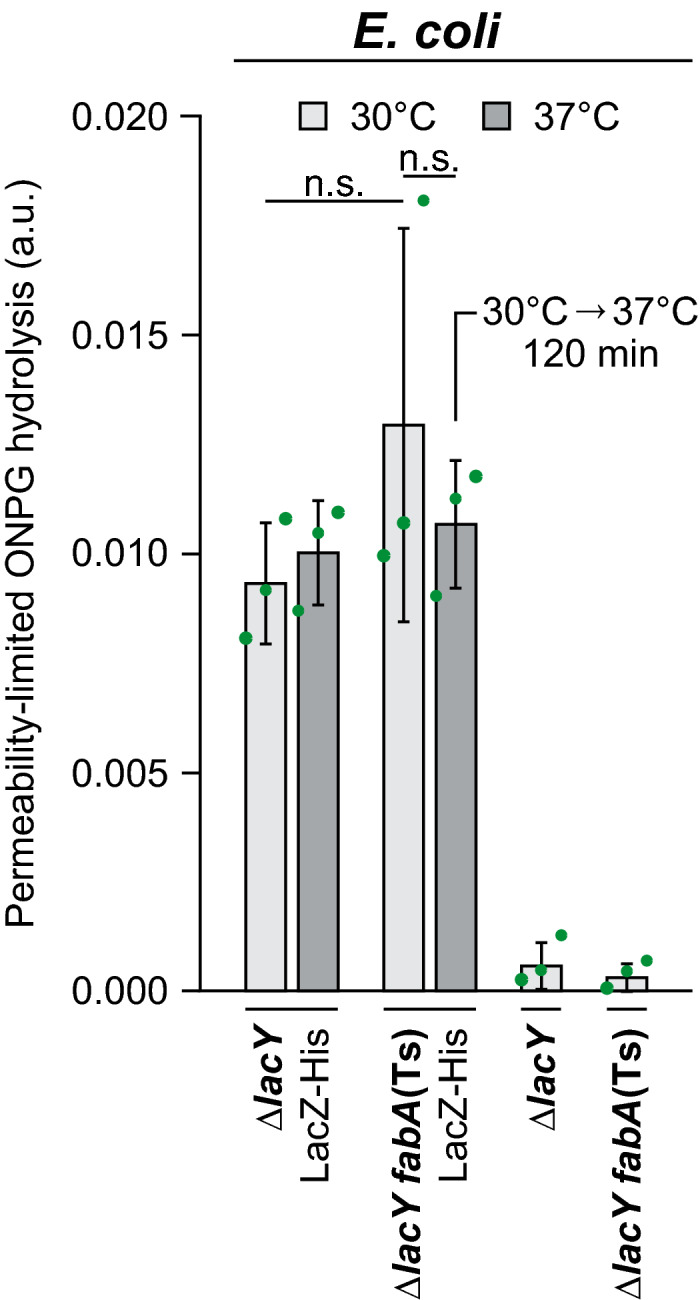
Very low membrane fluidity in *E. coli* does not trigger membrane permeabilisation for *ortho*‐nitrophenyl β‐D‐galactopyranoside (ONPG) The membrane permeability of ONPG was assessed in strains deficient for the active uptake system LacY simultaneously expressing *lacZ* from a plasmid‐encoded leaky *Ptac* promoter (without addition of the inducer IPTG). The graph depicts ONPG hydrolysis rates measured for intact cells upon incubation at 30 and 37°C. In case of the temperature‐sensitive *fabA*(Ts) strain, growth at the non‐permissive temperature of 37°C was limited to 120 min. As controls, the ONPG conversion rates were measured in strains lacking the *lacZ*‐expressing plasmid. Note the lack of significant difference in ONPG conversion rates upon strong depletion of unsaturated fatty acids (*fabA*(Ts) strain at 37°C for 120 min), implying the lack of detectable membrane permeabilisation due to phase separation. Data information: The graph depicts mean and SD of biological triplicates. The *P* values represent the results of unpaired, two‐sided *t*‐tests. Insignificant changes (*P* > 0.1) are indicated with n.s. Strains used: *E. coli* Y‐Mel.Δ*lacY*, UC1098.Δ*lacY*, Y‐Mel.Δ*lacY*/pTM30.*lacZ‐*His2, UC1098.Δ*lacY*/pTM30.*lacZ*‐His2. Source data are available online for this figure.

High DiSC_3_(5) fluorescence signals and, thus, high membrane potential levels were also observed both for *E. coli* WT and *fabA*(Ts) grown at the permissive temperature of 30°C (Fig 3C and D, and Appendix Fig S3D and E), whereas depletion of fluidity‐promoting UFA in *E. coli fabA*(Ts) triggered a gradual loss of membrane potential. However, compared to *B. subtilis* the loss was delayed and incomplete (Fig 3D). Again, the lack of Sytox Green staining revealed that the membranes were not impaired in their general diffusion barrier function (Fig 3C). To confirm this important finding, membrane permeability was also monitored by following LacZ‐dependent hydrolysis of the chromogenic substrate ONPG (*ortho*‐nitrophenyl β‐D‐galactopyranoside) in cells deficient for the uptake system LacY. While we were able to detect low level, LacZ‐dependent ONPG hydrolysis in a *lacY* deletion background, no difference was observed between cells with native fatty acid composition and cells strongly depleted for UFAs undergoing lipid phase separation. Hence, the results provide an independent control for the lack of membrane permeabilisation (Fig EV1).

In summary, while membrane depolarisation is observed due to too low membrane fluidity in both organisms, the core permeability function of the plasma membrane is not compromised even upon conditions unable to support growth. This is consistent with a more subtle effect of low fluidity on membrane‐associated biological processes maintaining ion homeostasis such as respiration.

### Consequences of low membrane fluidity on cell morphogenesis

In rod‐shaped bacteria, cell growth and morphogenesis are predominantly driven by two membrane‐associated multiprotein complexes, the elongasome responsible for envelope expansion and rod shape determination (Typas *et al*, 2012), and the divisome responsible for cytokinesis (Adams & Errington, 2009). The main scaffold proteins for these prominent cellular machineries are the tubulin homolog FtsZ (Adams & Errington, 2009) and the actin homolog MreB (Typas *et al*, 2012). To assess the functionality of these key cellular machineries, we determined the localisation of corresponding GFP fusions upon depletion of fluidity‐promoting fatty acids. Furthermore, by use of GFP‐fused DNA‐binding protein Hu (*B. subtilis*) (Köhler & Marahiel, 1997) or DNA staining with intercalating dye DAPI (*E. coli*), we analysed the cells for potential defects in chromosome replication and segregation.

**Figure 4:**
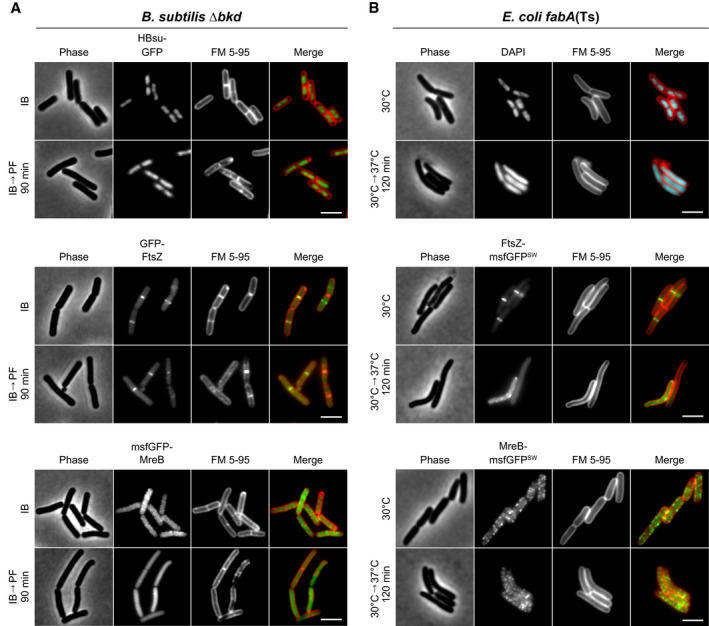
Consequences of low membrane fluidity on and cell morphogenesis Images of *B. subtilis* Δ*bkd* cells stained with membrane dye FM 5‐95 and expressing GFP fusions of DNA‐binding protein HBsu (top), cell division protein FtsZ (middle) or cell elongation protein MreB (bottom). Cells were grown with IB or depleted for precursors for 90 min (IB→PF). For further examples and additional time points, see Appendix Fig S4A.Images of *E. coli fabA*(Ts) cells stained with FM 5–95 for the outer membrane and with DNA‐intercalating dye DAPI (top) or expressing GFP sandwich (SW) fusions to the cell division protein FtsZ (middle) and the cell elongation protein MreB (bottom), respectively. Depicted are cells grown at 30°C or with a temperature shift to 37°C for 120 min. For a more detailed view on the influence of low membrane fluidity on membrane dissociation of RNase E as well as on the cell division machinery, see Figs EV2, EV3, EV4, EV5 and Appendix Fig S5. For further examples and additional time points, see Appendix Fig S4B. Data information: (A, B) The experiments are representative of biological triplicates. Scale bar, 3 µm. Strains used: (A) *B. subtilis* HS541, HS548, HS549; (B) *E. coli* UC1098, BHH500, BHH501.

**Figure EV2:**
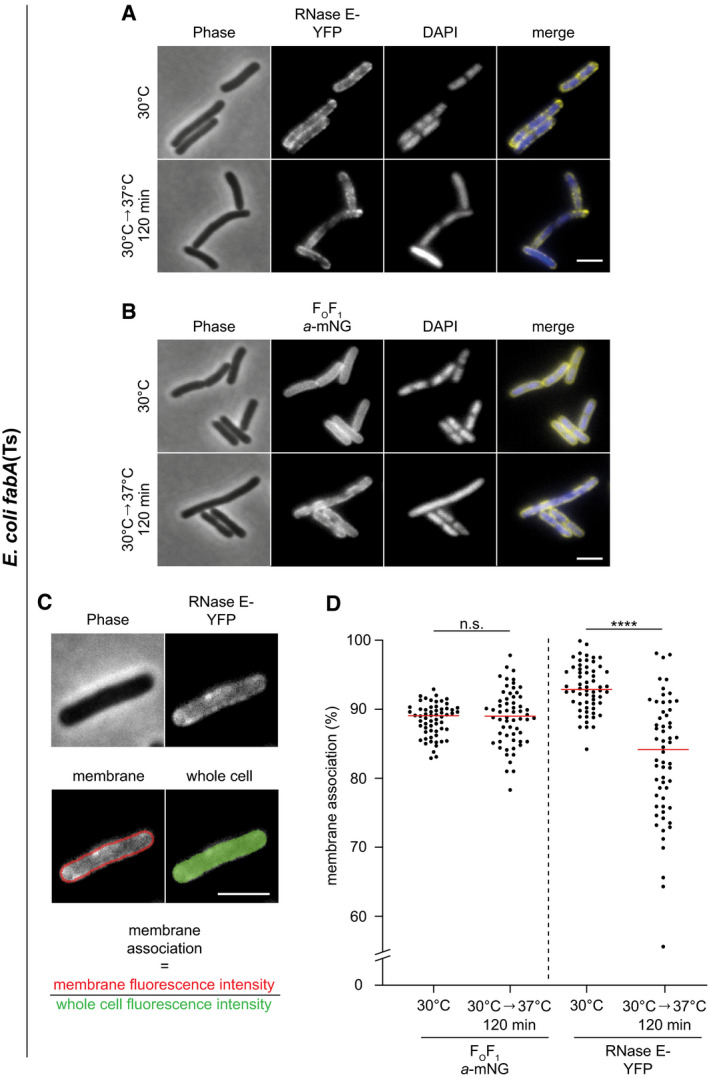
Very low membrane fluidity triggers partial dissociation of RNase E from the membrane in *E. coli fabA*(Ts) A, BPhase‐contrast and fluorescence images of *E. coli fabA*(Ts) strain expressing (A) RNase E‐YFP or (B) F_O_F_1_ *a*‐mNG. Cells were grown in LB at 30°C to an OD_600_ of 0.3, transferred to the non‐permissive temperature of 37°C for 120 min followed by labelling with DAPI and fluorescence microscopy. Note the increasing cytoplasmic localisation of RNase E‐YFP upon depletion of the membrane for UFA, which coincides with decondensation of the nucleoid (compare Fig 4B).CQuantification of membrane association of RNase E. The degree of membrane association was quantified by automated detection of cells using phase‐contrast images, defining a 3‐pixel wide band around the periphery of the cell, and measuring the relative membrane association as a ratio between the mean peripheral fluorescence signal and the mean fluorescence of the whole cell.DRelative membrane association of F_O_F_1_ *a*‐mNG and RNase E‐YFP at 30°C and 37°C for 120 min in individual cells (*n* = 60). Red lines indicate the median. Data information: (A–D) Experiments are representative of independent biological duplicates. (D) Red lines indicate the median. *P* values represent the results of unpaired, two‐sided *t*‐tests. Significance was assumed with ****P < 0.0001, n.s., not significant (A–C). Scale bar: 3 µm. Strains used: (A, C, D) *E. coli* UC1098/pVK207; (B, D) *E. coli* MG4. Source data are available online for this figure.

**Figure EV3:**
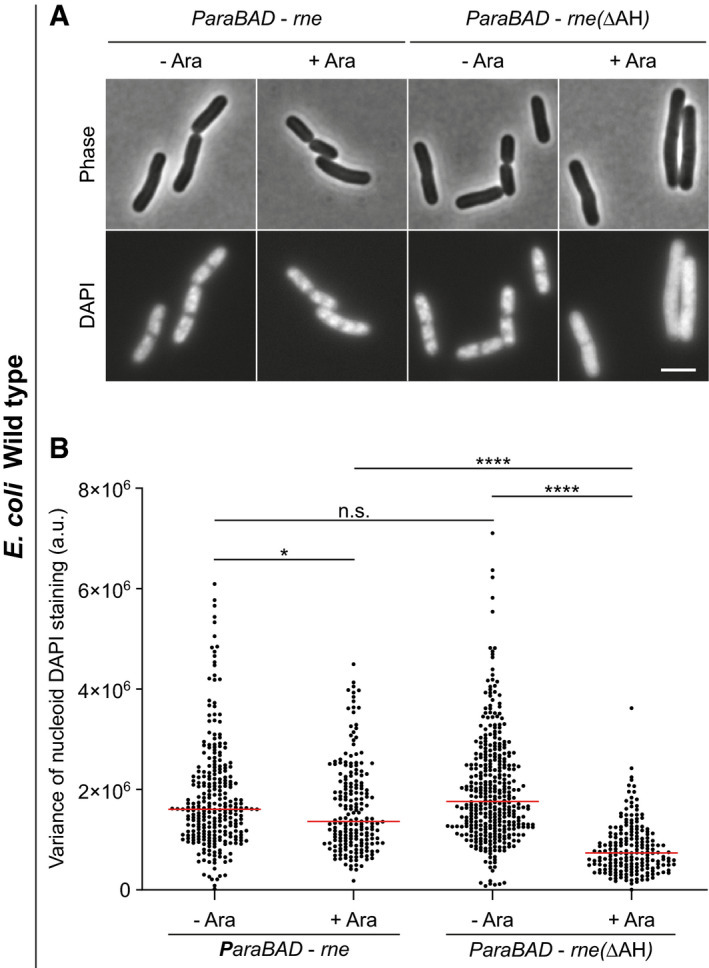
Expression of cytoplasmic RNase E is sufficient to trigger decondensation of the nucleoid Phase‐contrast and fluorescence images of *E. coli* WT cells expressing plasmid‐encoded full‐length membrane‐associated RNase E (Rne) and a corresponding construct encoding RNase E that lacks the membrane‐binding amphipathic helix (RneΔAH), respectively. The cells depicted were grown in LB medium at 37°C to an OD_600_ of 0.3 followed by induction of *rne* with 0.2% (w/v) arabinose (Ara) for 60 min, labelling with DAPI for 15 min and fluorescence microscopy. Note the decondensation of the nucleoid observed upon expression of cytoplasmically located RneΔAH, but not in the presence of the native membrane‐associated RNase E.Variance of nucleoid staining. The degree of nucleoid condensation was assessed by analysing the variance of DAPI fluorescence within the cell (*n* = 199–372). In this type of analysis, a more homogeneous fluorescence signal such as that caused by nucleoid decondensation results in a lower variance of the per pixel fluorescence intensity. Red lines indicate the median. Data information: (A, B) The experiments are representative of biological duplicates. (B) Red lines indicate the median. *P* values represent results of unpaired, two‐sided *t*‐tests. Significance was assumed with ****P < 0.0001, *P < 0.05, n.s., not significant. (A) Scale bar: 3 µm. Strains used: (A, B) *E. coli* MG1655/pJG130, MG1655/pJG131. Source data are available online for this figure.

**Figure EV4:**
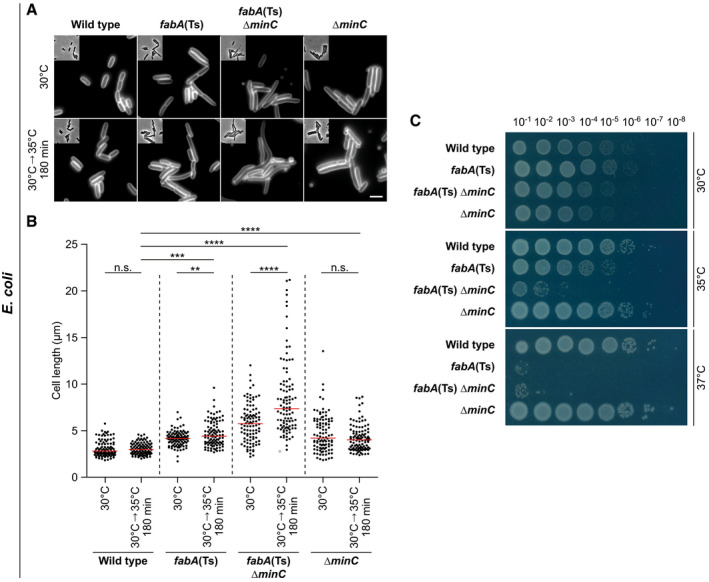
Destabilisation of *E. coli* divisome by deletion of the division regulator *minC* triggers hypersensitivity towards low membrane fluidity Images of *E. coli* WT, *fabA*(Ts), *fabA*(Ts) Δ*minC and* Δ*minC* cells grown either at the permissive temperature (30°C) or for 180 min at 35°C, which is non‐permissive for the *fabA*(Ts) Δ*minC* strain. Cells were stained with the outer membrane dye FM 5–95 prior to microscopy. Note the strong cell elongation of the *fabA*(Ts) Δ*minC* strain upon incubation at 35°C, which is indicative of a severe cell division defect.Quantification of cell length for cells (*n* = 100) depicted in panel A. Red lines indicate the median.Viability of the strains depicted in panel A upon incubation on agar plates in M9‐glucose minimal medium overnight at different temperatures. The serial dilutions and spot assays were carried out with pre‐cultures grown at 30°C to mid‐log growth phase. Note the temperature hypersensitivity and loss of viability of strain *fabA*(Ts) Δ*minC* at 35°C, which indicates that the cell division process has become the limiting factor in tolerance towards low membrane fluidity in this strain. The temperature sensitivity of the strains *fabA*(Ts) Δ*zapA* and *fabA*(Ts) Δ*zapB* supported this view (see Appendix Fig S5). Data information: (A–C) The experiments are representative of biological triplicates. (B) Red lines indicate the median, while the *P* values represent results of unpaired, two‐sided *t*‐tests. Significance was assumed with ****P < 0.0001, ***P < 0.001, **P < 0.01, n.s., not significant. (A) Scale bar: 3 µm. Strains used: (A–C) *E. coli* Y‐Mel, UC1098, UC1098.Δ*minC*, JW1165‐1. Source data are available online for this figure.

**Figure EV5:**
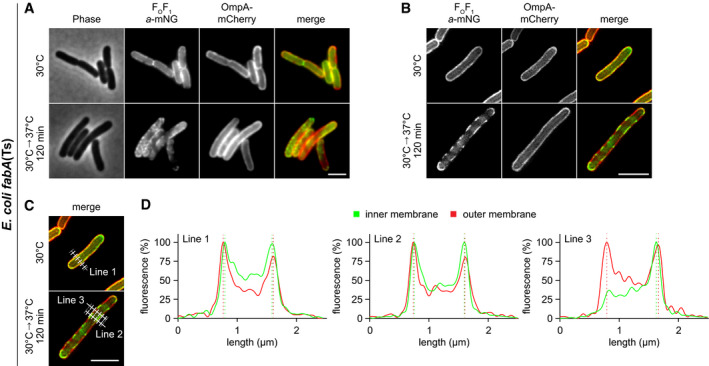
Protein segregation induced by very low membrane fluidity is limited to the inner cytoplasmic membrane in *E. coli* Membrane protein segregation was monitored in *fabA*(Ts) cells expressing as inner membrane marker F_O_F_1_ *a*‐mNG and as outer membrane marker OmpA‐mCherry. Widefield microscopy images depict phase‐contrast, fluorescence and overlay images for cells grown at permissive 30°C or at non‐permissive 37°C for 120 min in M9‐glucose minimal medium. Note the transition from disperse localisation into a segregated pattern in case of the inner membrane‐localised F_O_F_1_ *a*‐mNG at 37°C, while the pattern of the outer membrane‐localised OmpA‐mCherry remains homogeneous at both growth temperatures.Super‐resolution 2D‐SIM (structured illumination microscopy) images of cells expressing F_O_F_1_ *a*‐mNG and OmpA‐mCherry at both growth temperatures.Localisation and orientation of 5‐pixel wide lines used to analyse fluorescence intensity profiles depicted in panel D.Fluorescence intensity line scans across the cells imaged with 2D‐SIM microscopy. Note the small, but detectable outward shift between the inner membrane marker F_O_F_1_ *a*‐mNG and outer membrane marker OmpA‐mCherry. Data information: (A–D) Experiments are representative of biological triplicates (A–C). Scale bar: 3 µm. Strain used: (A–D) *E. coli* MG4/pGI10. Source data are available online for this figure.

In *B. subtilis*, depletion of BCFAs had no effect on nucleoid prevalence or morphology indicating the presence of largely functional DNA replication, segregation and compaction mechanisms (Fig 4A and Appendix Fig S4A). While no DNA‐free cells indicative for defects in DNA replication and segregation were observed in *E. coli* either, a clear de‐condensation of the nucleoid was evident at later stages of UFA depletion (Fig 4B and Appendix Fig S4B). Intriguingly, this process coincides with partial dissociation of RNA degradosome from the membrane as indicated by an increasingly cytoplasmic localisation of the key scaffold and membrane anchor protein RNase E in cells exhibiting very low fluidity (Fig EV2, EV3, EV4, EV5). These two processes appear to be causally linked since expression of a cytoplasmic variant of RNase E, which lacks an amphipathic helix essential for membrane binding, leads to a comparable decondensation of the nucleoid (Fig EV3A and B).

The cell division machinery, indicated by mid‐cell localisation of FtsZ, turned out to be robust towards changes in membrane fluidity in *B. subtilis*, with only a weakening of the fluorescent mid‐cell signal observed upon BCFA depletion (Fig 4A and Appendix Fig S4A). In contrast, a clear defect in divisome assembly was observed upon depletion of UFA in *E. coli* (Fig 4B and Appendix Fig S4B). To confirm that the *E. coli* cell division machinery indeed is stressed by low membrane fluidity, we combined the UFA depletion with a deletion of cell division regulator MinC (Hu *et al*, 1999). Indeed, in the absence of MinC, the *E. coli* cell division process became the growth‐limiting factor upon UFA depletion and even a slight shift in temperature from 30 to 35°C resulted in hypersensitivity towards low fluidity and loss of viability for strain *fabA*(Ts) Δ*minC* (Fig EV4, EV5). A clear increased sensitivity towards low membrane fluidity was also observed for strains deficient for the division proteins ZapA or ZapB (Appendix Fig S5) (Galli & Gerdes, 2010).

An inverse sensitivity was observed for the cell elongation machinery using localisation of MreB as proxy. In this case, depletion of BCFA triggered a complete disassembly of the MreB cytoskeleton in *B. subtilis* (Fig 4A and Appendix Fig S4A), whereas the *E. coli* counterpart was largely unaffected from UFA depletion in terms of membrane association and filament formation (Fig 4B and Appendix Fig S4B).

In conclusion, very low membrane fluidity incapable to support growth indeed affects membrane‐associated cellular machineries responsible for bacterial growth and division. It is important to note, however, that the changes in membrane fluidity required to disturb cell morphogenesis are rather extreme and go well beyond those observed upon normal changes in growth temperature.

### Consequences of low membrane fluidity on membrane homogeneity

**Figure 5:**
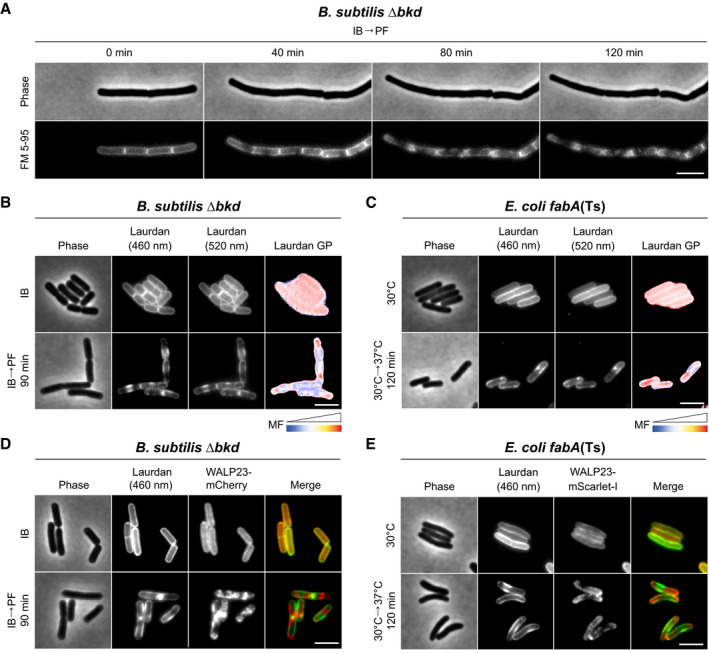
Consequences of low membrane fluidity on membrane homogeneity Time lapse images of *B. subtilis* Δ*bkd* cells stained with FM 5–95 and grown in PF medium. See Movie EV1 for full time lapse. Appendix Fig S6 shows the changes observed in relation to natural homeoviscous adaptation.Images of *B. subtilis* Δ*bkd* cells grown with IB or without precursor (IB→PF). Cells were stained with membrane fluidity‐sensitive dye Laurdan and imaged at 460 nm, 520 nm and as the corresponding colour‐coded Laurdan GP map.Images of *E. coli fabA*(Ts) cells grown at 30°C or shifted to non‐permissive 37°C for 120 min. Cells were stained and imaged as described in panel B. For a more pronounced view on domain formation associated with local differences in membrane fluidity, see Appendix Fig S7, showing *E. coli fabA*(Ts) grown in LB instead of M9 minimal medium with glucose/casamino acids.Images of *B. subtilis* Δ*bkd* cells grown and stained with Laurdan (imaged at 460 nm) as in panel B, but additionally expressing WALP23‐mCherry. For corresponding fluorescence intensity correlation between images, see Appendix Fig S8A. Co‐localisation of membrane dye FM 5‐95 and transmembrane peptide WALP23 is shown in Appendix Figs S9 and S8C.Images of *E. coli fabA*(Ts) cells grown and stained as in panel C, but additionally expressing WALP23‐mScarlet‐I. For corresponding fluorescence intensity correlation between images, see Appendix Fig S8B. Data information: (A–E) The experiments are representative of biological triplicates. Scale bar: 3 µm. MF, membrane fluidity. Strains used: (A, B) *B. subtilis* HS527; (C) *E. coli* UC1098; (D) *B. subtilis* HS547; (E) *E. coli* UC1098/pBH501.

In the microscopic experiments described above (Fig 4A and B), cells were stained with FM 5‐95. This hydrophobic fluorescent dye allows visualisation of the plasma membrane of *B. subtilis* (Sharp & Pogliano, 1999), or the outer membrane in the case of *E. coli* (Pilizota & Shaevitz, 2012). While no changes in FM 5–95 staining were observed in *E. coli* cells, the smooth staining observed in Δ*bkd* cells supplemented with IB transitioned into a distinctly irregular pattern upon BCFA depletion (Fig 4A and Appendix Fig S4A). This suggests that the more homogeneous membrane, present under normal growth conditions, segregates into areas with different local physicochemical properties upon low membrane fluidity. Intriguingly, the development of membrane irregularities coincides with growth arrest (Fig 5A and Movie EV1). It is worth emphasising, however, that the changes in lipid fatty acid composition needed to trigger this response are rather extreme compared to changes observed upon normal homeoviscous adaptation (Appendix Fig S6A and B).

*In vitro*, lipid mixtures of low fluidity undergo phase transition into a more tightly packed gel state. We speculated that the lipid de‐mixing observed in *B. subtilis* (Figs 4A and 5A) could therefore represent large‐scale lipid phase separation between gel and liquid‐disordered phases. Therefore, we analysed the local membrane fluidity of both *B. subtilis* and *E. coli* with the fluidity‐sensitive membrane dye Laurdan. Laurdan exhibits shifts in its fluorescence emission spectrum that depends on lipid packing‐linked penetration of H_2_O to the membrane interior and, thus, the immediate environment surrounding the fluorophore. The interconnected nature of lipid packing and membrane fluidity allows local membrane fluidity to be estimated as Laurdan generalised polarisation (GP) (Parasassi *et al*, 1990; Wenzel *et al*, 2018). Indeed, when *B. subtilis* and *E. coli* cells were depleted for fluidity‐promoting fatty acids, domain formation associated with both differential staining (Laurdan fluorescence intensity) and differences in local fluidity (Laurdan GP) was observed (Fig 5B and C, and Appendix Fig S7). To verify these findings with an independent dye‐free method, we used cells expressing helical transmembrane peptide WALP23, which preferentially accumulate in liquid‐disordered membrane areas (Schäfer *et al*, 2011; Scheinpflug *et al*, 2017b). Indeed, co‐labelling of cells with Laurdan clearly demonstrated that the observed lipid phase separation results in segregation of WALP23 in membrane areas of low Laurdan fluorescence (Fig 5D and E, and Appendix Fig S8A and B). At last, co‐staining with FM 5–95 demonstrated that FM 5–95 and WALP23 share the same preference for higher fluidity areas in de‐mixed membranes (Appendix Figs S8C and S9).

In conclusion, by exhibiting de‐mixing into distinct areas of high and low membrane fluidity, respectively, the observed *in vivo* domain formation shares the core characteristic of lipid phase separation between fluid and gel state membranes (Baumgart *et al*, 2007; Domański *et al*, 2012; Mostofian *et al*, 2019).

### Segregation of membrane proteins into fluid domains of phase‐separated plasma membranes

As indicated by WALP23 (Fig 5D and E), the observed lipid phase separation might have broader consequences on membrane protein localisation. To test this, we focused on *E. coli* for two reasons. Firstly, the membrane depolarisation caused by low membrane fluidity, which itself can affect membrane protein localisation (Strahl & Hamoen, 2010), is less extensive in *E. coli* (Fig 3). Secondly, *E. coli* does not exhibit delocalisation of MreB (Fig 4B and Appendix Fig S4B), which we have previously shown to induce membrane protein clustering (Strahl *et al*, 2014). As a model protein of choice, we focused on ATP synthase (F_O_F_1_), an abundant polytopic membrane protein complex (Junge & Nelson, 2015).

**Figure 6:**
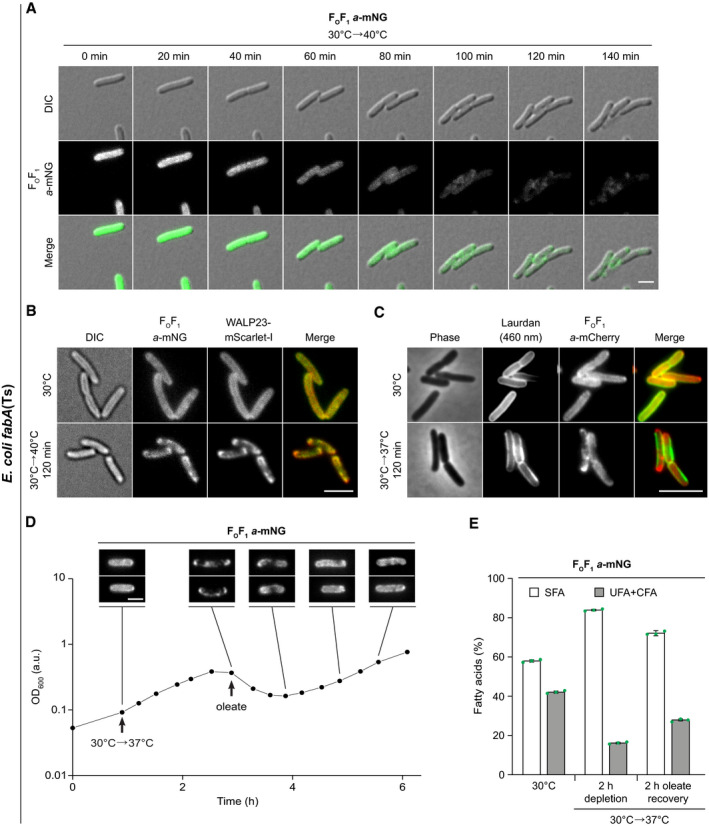
Partitioning of membrane proteins into fluid domains of phase‐separated plasma membranes Time lapse images of *E. coli fabA*(Ts) cells expressing F_O_F_1_ *a*‐mNG. For corresponding controls, see Appendix Fig S10. Cells were grown at 30°C and shifted from 30°C to non‐permissive 37°C. See Movie EV2 for full time lapse.Images of *E. coli fabA*(Ts) co‐expressing WALP23‐mScarlet‐I and F_O_F_1_ *a*‐mNG grown at permissive 30°C or shifted to non‐permissive 40°C. For fluorescence intensity correlations, see Appendix Fig S8D. For corresponding images of *E. coli fabA*(Ts) cells co‐expressing inner membrane marker F_O_F_1_ *a*‐mNG and outer membrane marker OmpA‐mCherry, see Fig EV5.Images of F_O_F_1_ *a*‐mCherry‐expressing *E. coli fabA*(Ts) grown at 30°C or shifted to non‐permissive 37°C and stained with Laurdan. For fluorescence intensity correlations, see Appendix Fig S8E.Growth behaviour of F_O_F_1_ *a*‐mNG expressing *E. coli fabA*(Ts) after shift from 30°C to non‐permissive 37°C and upon recovery through exogenous supplementation with UFA oleate (*cis*‐Δ9‐C18:1). The corresponding reversible segregation of F_O_F_1_ *a*‐mNG is shown above the growth curve. For further controls, see Appendix Fig S11A. For a comparable fluorescence time lapse analysis of mNG‐labelled glucose permease PtsG, see Appendix Fig S12.Fatty acid composition of *E. coli fabA*(Ts) cells (same cell batch as in D) upon growth at 30°C, upon depletion of UFA by incubation at 37°C for 120 min and upon recovery by oleate supplementation for additional 120 min. For detailed analyses, see Appendix Fig S11B–D. Data information: (A–D) The experiments are representative of biological triplicates. (E) The histogram depicts mean and SD of biological triplicates. DIC, differential interference contrast. Scale bar: (A, B, D) 2 µm; (C) 3 µm. Strains used: (A, D, E) *E. coli* MG4; (B), *E. coli* MG4/pBH501; (C), *E. coli* LF6.red. Source data are available online for this figure.

To visualise the localisation of F_O_F_1_, fluorescent protein was C‐terminally fused to membrane‐integral F_O_‐*a* yielding a stable and active enzyme (Appendix Fig S10A and B). Upon UFA depletion, F_O_F_1_ showed clear segregation behaviour (Fig 6A and Movie EV2). When co‐expressed, F_O_F_1_ and WALP23 showed clear co‐segregation into the fluid areas of phase‐separated membranes (Fig 6B and Appendix Fig S8D). This property was also confirmed by co‐staining with Laurdan showing an anti‐correlation of the fluorescent signals (Fig 6C and Appendix Fig S8E). In agreement with the smooth outer membrane staining using FM 5–95 (Fig 4B and Appendix Fig S4B), no segregation was observed for the major outer membrane protein OmpA. Co‐labelling of the inner and outer membrane with fluorescent proteins analysed by super resolution structured illumination microscopy demonstrated that the inner membrane marker F_O_F_1_ *a*‐mNG showed segregation into the fluid areas at non‐permissive 37°C, while the pattern of the outer membrane marker OmpA‐mCherry remains homogeneous, thus supporting the view that the outer membrane does not participate in the phase separation process (Fig EV5A–D).

Depletion of UFAs had no substantial influence on the DCCD‐sensitive ATPase activity of F_O_F_1_ (Appendix Fig S10B), thus arguing that high viscosity of the surrounding lipids does not significantly hinder F_O_F_1_ in its rotation‐based catalytic cycle (Junge & Nelson, 2015). In a wider context, this indicates that the remaining fluid phase, to which F_O_F_1_ partitions upon UFA depletion, can retain robust bioactive properties. Motivated by this finding, we analysed whether the lipid phase separation and the associated growth arrest is reversible. Indeed, when severely UFA‐depleted *fabA*(Ts) cells exhibiting both growth arrest and lipid phase separation were exogenously supplied with oleate (*cis*‐Δ9‐C18:1), incorporation of oleate into phospholipids, together with growth recovery and restoration of the dispersed distribution of F_O_F_1_ was observed (Fig 6D and E, and Appendix Fig S11A–D). Comparable experiments performed with fluorescently labelled glucose permease (PtsG‐mNG) confirmed that the observed protein segregation and its recovery (Appendix Fig S12A–C) is not unique for ATP synthase.

In summary, our results demonstrate that lipid phase separation occurring under conditions of low membrane fluidity has a profound effect on membrane protein distribution, triggering segregation of integral membrane proteins into the remaining liquid‐disordered phase areas.

### Restricted diffusion of membrane proteins in UFA‐depleted *E. coli* membranes

**Figure 7:**
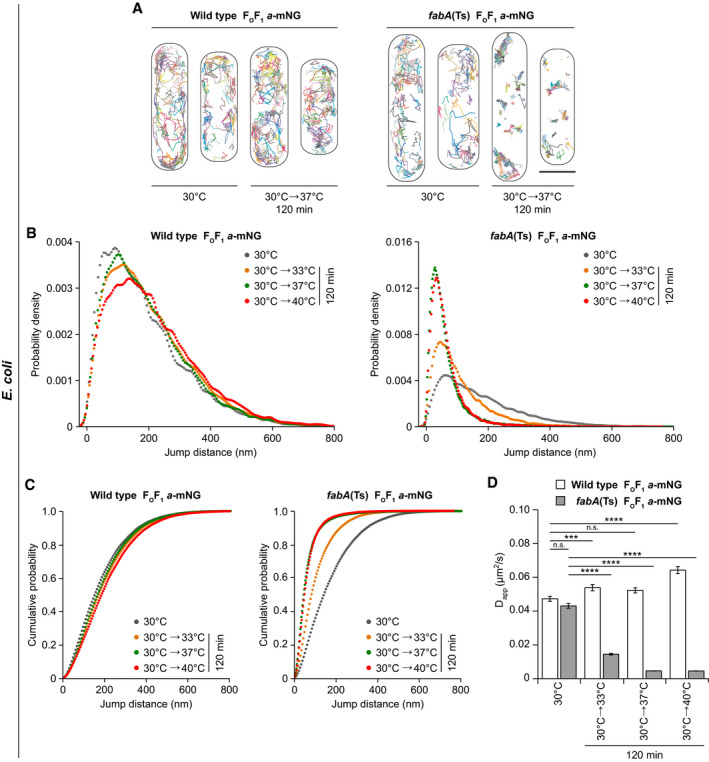
Restricted diffusion of membrane proteins in UFA‐depleted *E. coli* membranes Representative trajectory maps of individual F_O_F_1_ *a*‐mNG molecules in *E. coli* WT and *fabA*(Ts) cells grown at 30°C and upon shift to non‐permissive 37°C for 120 min. See Movie EV3 (WT) and Movie EV4 (*fabA*(Ts)) for accumulating trajectory maps of F_O_F_1_ *a*‐mNG molecules.Probability density plots of F_O_F_1_ *a*‐mNG jump distances in *E. coli* WT and *fabA*(Ts) cells.Cumulative probability plots of F_O_F_1_ *a*‐mNG jump distances in *E. coli* WT and *fabA*(Ts) cells. For an analysis of cell‐to‐cell heterogeneity of median jump distances of F_O_F_1_ *a*‐mNG, see Appendix Fig S13A.Apparent lateral diffusion coefficients (D_app_) of F_O_F_1_ *a*‐mNG analysed in panel B. An analysis of cell‐to‐cell heterogeneity of D_app_ is shown in Appendix Fig S13B. For a comparable single molecule tracking analysis of transmembrane peptide WALP23‐mNG, see Appendix Fig S14 and Appendix Table S3. A detailed analysis of F_O_F_1_ *a*‐mNG in an osmotically stabilised *fabB1*5(Ts) mutant (2% KCl) at non‐permissive 40°C is shown in Appendix Figs S15 and S16, Appendix Table S4. Data information: (A) Representative for 3–5 biological replicates. (B, C) Trajectories with ≥ 5 consecutive frames for each growth condition and strain were pooled from 3 to 5 biological replicates (*n* = 2,345–4,468). See Appendix Table S2 for detailed information on cell numbers and jump distances. (D) Median and SD from 3 to 5 biological replicates, together with *P* values of a two‐sided Wilcoxon rank sum test. Significance was assumed with ****P < 0.0001, ***P < 0.001, n.s., not significant. (A) Scale bar: 1µm. Strains used: (A–D), *E. coli* MG1, MG4. Source data are available online for this figure.

To analyse the consequences of UFA depletion on protein diffusion and, thus, membrane fluidity directly, we followed mNG‐labelled F_O_F_1_ (F_O_F_1_ *a*‐mNG) by *in vivo* single molecule tracking. Consistent with the lack of a specific localisation pattern, F_O_F_1_ *a*‐mNG complexes exhibited free diffusion within the plasma membrane plane of *E. coli* WT cells (Fig 7A and Movie EV3). The observed lateral mobilities and jump distances were largely independent of the growth temperature (Fig 7B and Appendix Fig S13A), as expected for cells with active homeoviscous adaptation mechanisms. F_O_F_1_ *a*‐mNG expressed in *fabA*(Ts) cells at 30°C also showed unrestricted lateral mobility comparable to WT. Under conditions of UFA depletion (33–40°C), however, a gradual, temperature‐dependent reduction of lateral displacement and median jump distances was observed (Fig 7A–C, Movie EV4, and Appendix Table S2). This is consistent with either significantly reduced diffusion or a local confinement of F_O_F_1_ caused by co‐occurring lipid phase separation (compare Fig 6A–C). Calculation of apparent lateral diffusion coefficients (D_app_) revealed that the lateral mobility was reduced up to 9‐fold (Fig 7D). Comparable results were obtained when the median jump distances and D_app_ were analysed for cell‐to‐cell heterogeneity (Appendix Fig S13A and B). The D_app_ of 0.0474 ± 0.0015 µm^2^/s determined here for F_O_F_1_‐*a*‐mNG at 30°C corresponds well with the values obtained previously for mEOS3.2‐F_O_F_1_ (0.042 ± 0.011 µm^2^/s at 22°C and 0.054 ± 0.014 µm^2^/s at 37°C, respectively) (Renz *et al*, 2015). These diffusion values are in also in agreement with recent FRAP (fluorescence recovery after photobleaching) and single molecule tracking data for cytoplasmic membrane proteins in *E. coli* ranging from 0.01 to 0.2 µm^2^/s (Leake *et al*, 2008; Kumar *et al*, 2010; Oswald *et al*, 2016).

WALP23‐mNG also exhibited rapid, unconfined diffusion when expressed in *fabA*(Ts) cells at 30°C. As expected due to its single transmembrane helix (Ramadurai *et al*, 2009; Lucena *et al*, 2018), the median jump distances and D_app_ of WALP23 (Appendix Fig S14A–D and Appendix Table S3) were higher than those observed for F_O_F_1_. Upon lipid phase separation caused by UFA depletion, WALP23 exhibited confined mobility comparable to that observed for F_O_F_1_, again supporting the notion that both proteins co‐segregate into the remaining fluid phase (Fig 6B and Appendix Fig S8D and E).

Due to the membrane phase separation resulting in protein‐free gel phase regions (Figs 5C and E, and 6B and C), membrane proteins become concentrated in the remaining fluid phase areas, while the membrane plane available for lateral diffusion is strongly reduced (Figs 5, 6, 7). Hence, it is not straightforward to distinguish whether the reduced diffusion dynamics are due to a change in the diffusion coefficient or due to a phase separation‐driven local confinement. While qualitative, the observed displacements appear to be significantly smaller than the remaining fluid phase membrane areas (compare Figs 5C and E, and 6B–D), thus somewhat arguing against confinement as the sole reason for the reduced diffusion dynamics. A reduction of the diffusion coefficient in protein‐overcrowded membrane areas would also be consistent with previous *in vitro* work demonstrating a linear decrease of both protein and lipid lateral mobility with increasing membrane protein concentrations (Ramadurai *et al*, 2009).

Osmotic stabilisation has been described for some UFA auxotrophic *E. coli* strains (Broekman & Steenbakkers, 1973, 1974; Akamatsu, 1974). Whereas osmotic stabilisation does not restore the viability of *fabA*(Ts) strain UC1098 in non‐permissive temperatures (Akamatsu, 1974), a strain carrying a *fabB15*(Ts) mutation is able to grow at non‐permissive 40°C as long as an osmotic stabiliser (*e.g*. 2% KCl) is present (Akamatsu, 1974) (Appendix Fig S15A). Fluorescence microscopy of *fabB15*(Ts) cells expressing F_O_F_1_ *a*‐mNG revealed that protein partitioning observed in the presence of KCl is detectable but less severe (Appendix Fig S15B), while single molecule tracking of F_O_F_1_ *a*‐mNG showed a lesser reduction of lateral diffusion compared to *fabA*(Ts) (Appendix Fig S16A and Appendix Table S4). Crucially, fatty acid analyses (Appendix Fig S16B–D) showed that depletion of UFA is significantly less pronounced in this strain upon osmotic stabilisation with KCl. Hence, osmotic stabilisation of this strain does not rescue the cells from low UFA content but rather acts by partially restoring UFA synthesis.

**Figure 8:**
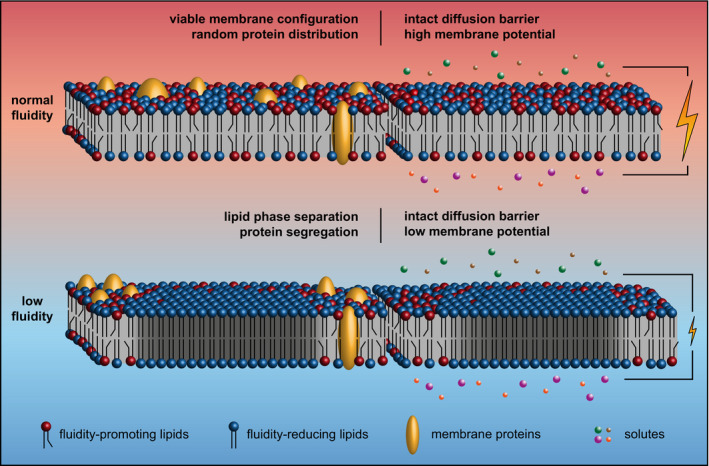
Low membrane fluidity triggers large‐scale gel‐liquid lipid phase separation *in vivo* The cartoon illustrates how too low membrane fluidity triggers large‐scale lipid phase separation associated with both segregation and confined diffusion of membrane‐integral proteins (left part). Too low membrane fluidity also results in dissipation of membrane potential likely due to a reduced electron transport chain activity (Budin *et al*, 2018). The core diffusion barrier function of the membrane, however, is maintained (right part).

In summary, depletion of UFA in *E. coli* results in a strong reduction of membrane fluidity that severely restricts lateral diffusion of membrane proteins (summarised in Fig 8). As a complementary, dye‐independent method, the tracking experiments also confirm the lipid phase separation phenomenon, resulting in integral membrane proteins segregated and confined into the remaining fluid membrane areas.

## Discussion

Consistent with early studies (Willecke & Pardee, 1971; Cronan & Gelmann, 1973; Kaneda, 1977; Boudreaux *et al*, 1981), the ability to maintain membrane fluidity through synthesis of fluidity‐promoting lipid species is indeed essential for the growth of both *E. coli* and *B. subtilis*. However, the magnitude of changes in composition and fluidity which the cells can tolerate is surprisingly large (summarised in Fig 8). While *B. subtilis* cells finely balance the ratio of *iso*‐ and *anteiso*‐BCFA in response to changes in temperature (Suutari & Laakso, 1992; Klein *et al*, 1999), even the massive changes in the *iso*/*anteiso*‐ratio obtained through precursor supplementation had no significant effect on growth behaviour. Similarly, changes in fatty acid composition and membrane fluidity needed to impair growth of *E. coli* are much more drastic than those observed as part of the normal lipid adaptation upon temperature shifts (Marr & Ingraham, 1962; Sinensky, 1974; Mansilla *et al*, 2004). Hence, while both *E. coli* and *B. subtilis* adapt their membrane composition and fluidity even upon subtle changes in temperature, the failure to do so is not associated with growth inhibitory consequences. This lack of clear phenotypes argues against maintaining membrane fluidity (homeoviscous adaptation) or membrane phase (homeophasic adaptation) as the physiological reason behind the tight temperature‐dependent regulation of membrane lipid composition in these model organisms. While it is possible that the optimal growth conditions applied here suppress phenotypes more evident upon stressful condition such as nutrient and O_2_ limitation, interspecies competition, or challenge with envelope‐targeting antimicrobial agents, it is important to emphasise that fluidity and phase are not the only parameters relevant for biological membranes. In fact, changes in temperature also induce substantial changes in lipid packing and membrane thickness (Szekely *et al*, 2011). While speculative, the temperature‐dependent lipid adaptation processes could have evolved to regulate membrane thickness rather than fluidity, thereby carefully maintaining appropriate hydrophobic shielding of integral membrane proteins in a changing environment.

In contrast to the smaller, physiologically more relevant changes discussed above, a more drastic reduction of membrane fluidity indeed has severe, growth inhibitory consequences. Whereas low membrane fluidity associated with lipid phase separation is accompanied by a substantial increase in membrane leakiness *in vitro* (Papahadjopoulos *et al*, 1973; Heimburg, 2007; Cordeiro, 2018), our results suggest that, at least in the context of the *in vivo* plasma membranes of *B. subtilis* and *E. coli*, such an effect does not play a significant role. Even the very low fluidity membranes incapable for supporting growth retain a robust diffusion barrier function. Rather than indicating ion leakage, the observed gradual and partial membrane depolarisation is fully consistent with previous reports, demonstrating that the membrane fluidity influences the electron transport chain (ETC) both in *E. coli* and mitochondria (Budin *et al*, 2018; Torres *et al*, 2018). While the enzyme complexes of the ETC maintain their function (as we also observed for F_O_F_1_), the diffusivity of ubiquinone is reduced in membranes of low fluidity and thus controls the electron transfer rate in the ETC (Budin *et al*, 2018). Therefore, maintaining robust ETC activity may well be one of the biological reasons why a fine, homeostatic balance of membrane fluidity is important.

In addition to changes in membrane potential, we also observed severe effects on the machineries responsible for cell morphogenesis. The reduction of membrane fluidity in *B. subtilis* is associated with rapid delocalisation of MreB indicating disturbance of lateral cell wall synthesis. Conversely, in *E. coli* both the cell division machinery and the nucleoid morphology were disturbed. However, both cell division and cell wall synthesis machineries are also influenced by membrane depolarisation (Strahl & Hamoen, 2010; Strahl *et al*, 2014). It therefore remains to be determined whether the observed changes are a direct consequence of low membrane fluidity, or secondarily caused by the gradual membrane depolarisation. It is worth highlighting though that the changes in membrane fluidity required to disturb cell morphogenesis are quite extreme and go well beyond those observed upon normal changes in growth temperature. Consequently, rather than as a sign for sensitivity, these findings provide an indication for the relative robustness of bacterial morphogenetic systems towards normally encountered changes in membrane fluidity.

The most striking phenomenon caused by severe reduction of membrane fluidity is the lipid de‐mixing associated with membrane protein segregation (summarised in Fig 8). Molecular dynamic simulations with membrane models composed of lipids with SFAs and BCFAs revealed increased ordering of the lipid bilayer when the SFA content was systematically increased. In the presence of approximately 20% SFAs (compared to 5–7% in *B. subtilis* WT cells), a sharp transition representing phase separation between liquid and gel phase was observed (Mostofian *et al*, 2019). These findings are fully consistent with the *in vivo* lipid de‐mixing we observe upon depletion of BCFAs in favour of SFAs accumulation in *B. subtilis*, albeit with a slightly higher SFA content needed. In *E. coli*, we observe *in vivo* lipid de‐mixing upon accumulation of SFA to a maximal content of 80% (compared to about 50% in WT (Zhu *et al*, 2009)), which again is consistent with previous *in vitro* studies regarding lipid phase separation of SFA/UFA mixtures (Letellier *et al*, 1977; Morein *et al*, 1996; Suárez‐Germà *et al*, 2011). For these reasons, we argue that the observed de‐mixing represents lipid phase separation between liquid‐disordered and gel state membranes, here occurring in intact, living cells. This phenomenon is likely comparable to that observed in eukaryotic endoplasmic reticulum, characterised by low cholesterol levels, upon metabolism of SFA C18:0 (Shen *et al*, 2017). While the phase separation phenomenon unarguably affects the plasma membranes both in Gram‐positive *B. subtilis* and Gram‐negative *E. coli*, we suggest that the process is limited to the plasma membrane and does not encompass the Gram‐negative outer membrane. This notion is based on an even distribution of a major outer membrane protein OmpA and smooth FM 5–95 outer membrane staining in cells with phase‐separated inner membranes.

The specificity of dyes to label different lipid phases is still a matter of debate. Small hydrophobic dyes may themselves alter the composition and ordering of coexisting phases, even when used in trace amounts (Veatch, 2007). However, we are convinced that (i) by using the combination of two chemically distinct fluorescent membrane dyes Laurdan and FM 5–95 exhibiting opposing phase preferences; (ii) by combining dye‐based approaches with localisation of WALP23 peptide previously shown to exhibit liquid‐disordered phase preference *in vivo*, *in vitro* and *in silico* (Ridder *et al*, 2004; Schäfer *et al*, 2011; Scheinpflug *et al*, 2017b); and (iii) by following the reversible phase separation through its consequences on lateral membrane protein diffusion, an approach completely independent of dyes but based on intrinsic chromosomal expression of proteins, we have exhausted the possibility that the observed phase separation is an artefact caused by the labelling techniques used.

Importantly, by demonstrating lipid liquid‐gel phase separation and the associated membrane protein segregation occurring in protein‐crowded, native membranes of living cells, our results are fully consistent with comparable phenomena observed in simplified *in vitro* and *in silico* model systems (Picas *et al*, 2010; Suárez‐Germà *et al*, 2011; Domański *et al*, 2012; Shaw *et al*, 2021), thus providing strong, complementary *in vivo* support for the general validity of the respective membrane models.

It is perhaps not surprising that the observed lipid phase separation coincides with growth arrest. Transmembrane segments of integral membrane proteins are embedded within the hydrophobic interior of lipid bilayers. Consequently, lipid bilayer thickness, which acutely changes with membrane fluidity and phase, is both important for membrane protein activity and drives partitioning of proteins between different phases (Lenaz, 1987; Lee, 2004; Lorent *et al*, 2017; Nickels *et al*, 2019). Peripheral membrane proteins, which establish membrane association through bilayer‐intercalating domains such as amphipathic helices, in turn rely on sufficiently low packing density/high fluidity for efficient membrane association (Drin & Antonny, 2010; Bigay & Antonny, 2012; Strahl & Errington, 2017). At last, the severe restriction of lateral diffusion caused by phase separation is likely interfering with localisation and activity of many membrane‐associated cellular processes relying on diffusion and capture mechanism (Rudner *et al*, 2002). In conclusion and as suggested earlier based on indirect evidence (Drobnis *et al*, 1993; Ghetler *et al*, 2005; Burns *et al*, 2017), we argue that it is indeed the lipid phase separation process and the formation of gel phase areas that determines the lower end of membrane fluidity capable of supporting viable cell functions.

## Materials and Methods

### Construction of *E. coli* strains

All *E. coli* strains used are listed in Appendix Table S1. *E. coli* strains MG1 or EB8.1 carrying a C‐terminal mNeonGreen (mNG) or mCherry fusion via a Gly‐Ser linker to the membrane‐integral F_O_‐*a* subunit, respectively, were generated using the phage λ Red recombinase to replace a chromosomal sequence (Datsenko & Wanner, 2000). Briefly, the kanamycin resistance cassette of strain EB4 (Δ*atpBE::*FRT‐*kan*‐FRT) was exchanged by the HindIII/AseI fragment of plasmids pBH189 (MG1) or pEB21.2 (EB8.1), followed by growth on M9 minimal medium with succinate (0.4% (w/v)) as sole carbon source for selection. As expected, both strains show a Succ^+^ Kan^S^ phenotype. In detail, EB4 cells transformed with temperature‐sensitive plasmid pKD46 encoding λ Red recombinase genes under control of the *ParaBAD* promoter were grown at 30°C to mid‐logarithmic phase in lysogenic broth (LB) composed of yeast extract (0.5% w/v), tryptone (1% w/v), NaCl (1% w/v) and supplemented with arabinose (0.2% w/v) for induction. Competent cells were prepared by wash with ice‐cold water for removal of salts and medium components, electroporated in the presence of corresponding DNA fragments, grown for 1 h in LB at 37°C for phenotypic expression and plated on selective solid medium.

For generation of *E. coli* strain UC1098. PtsG‐mNG, a 75 bp linker (encoding the amino acids EFTMVPAAPAPAAAAPAAAPTPASR) from plasmid pBLP2 (coding for a functional PtsG‐GFP fusion protein connected by the same linker region (Kosfeld & Jahreis, 2012)) and the open reading frame (ORF) encoding mNG (pNCS‐mNeonGreen) were inserted into the chromosomally encoded *ptsG* gene prior to its stop codon using λ Red mutagenesis (Datsenko & Wanner, 2000). Briefly, the kanamycin resistance cassette of strain UC1098.Δ*ptsG* (Δ*ptsG::*FRT‐*kan*‐FRT) (see below) was exchanged by a PCR product harbouring the *ptsG‐linker‐mNG* fusion gene flanked by upstream/downstream chromosomal regions of *ptsG* using growth on M9 minimal medium with glucose (0.4% (w/v)) for selection with *ptsG‐mNG*‐expressing colonies being significantly larger compared to those of precursor strain Δ*ptsG*. The PCR product was generated by a two‐step PCR overlap extension method. Firstly, four individual PCR products were generated using (i) oligonucleotides 1/2 with lysed cells of *E. coli* strain Y‐Mel as template, (ii) oligonucleotides 3/4 with pBLP2 as template, (iii) oligonucleotides 5/6 with pNCS‐mNeonGreen as template and (*iv*) oligonucleotides 7/8 again with lysed cells of Y‐Mel as template. Secondly, the four PCR products and oligonucleotides 1/8 were used for the second amplification step. Oligonucleotides used are listed in Appendix Table S5.

Several *E. coli* strains were obtained by P1 transduction (Thomason *et al*, 2007). In detail, P1 liquid lysate was generated by growing the donor strain to optical density at 600 nm (OD_600_) of 0.1 in LB (3 ml), adding CaCl_2_ (330 µl 50 mM) and P1 lysate (20 µl of ~10^−9^ phages/ml) and further growth with good aeration until lysis occurred. 5 drops of chloroform were added to lyse remaining cells, centrifuged twice to pellet debris, and the supernatant was stored with 50 µl of chloroform in the dark at 4°C. For transduction, an overnight culture of the recipient strain (200 µl) was mixed with CaCl_2_ (28 µl 50 mM) and P1 lysate (50 µl) of the donor strain and incubated for 20 min at 37°C. After addition of LB (0.7 ml) and Na_3_‐citrate (100 µl 1 M) and further incubation for 40 min, cells were plated on selective solid medium containing Na_3_‐citrate (20 mM).

Strains LF4 and AB1 were obtained using EB4 as donor, UC1098 and Cy288 as recipients, respectively, using LB with kanamycin (50 µg/ml) for selection leading to a Succ^+^ Kan^R^ phenotype. Subsequently, MG4 and LF6.red were generated by P1 transduction using MG1 and EB8.1 as respective donor strains, LF4 as recipient and M9 minimal medium with succinate (0.4% (w/v)) for selection. Strain BHH87 was generated in the same way as described for strain MG4. For generation of strains BHH100 and BHH101 by P1 transduction, MG1655.mreB‐msfGFP and KC555, respectively, were used as respective donor strains, UC1098 as recipient and LB with kanamycin (50 µg/ml) or chloramphenicol (12.5 µg/ml) for selection. For generation of strain UC1098.Δ*ptsG*, strain JW1087‐2 was used as donor, UC1098 as recipient and LB with kanamycin for selection. For generation of strains Y‐Mel.Δ*lacY* and UC1098.Δ*lacY*, strain JW0334‐1 was used as donor, Y‐Mel or UC1098 as recipients and LB medium with kanamycin for selection. For generation of strains UC1098.Δ*minC*, UC1098.Δ*zapA* and UC1098.Δ*zapB* (*yiiU* being renamed to *zapB*), strains JW1165‐1, JW2878‐1 and JW3899‐1, respectively, were used as donors, UC1098 as recipient and LB medium with kanamycin for selection. In all strains, the genes of interest were verified by colony PCR and DNA sequencing.

### Construction of *E. coli* plasmids

For construction of plasmids pBH189 and pEB21.2, a BamHI site (encoding a Gly‐Ser linker) and genes encoding mNG (pBH189) and mCherry (pEB21.2), respectively, were inserted into the *atp* operon of plasmid pBWU13 prior to the stop codon of *atpB* using a two‐step PCR overlap extension method. Firstly, three individual PCR products were generated using (i) oligonucleotides 9/10 with pSD166 as template, (ii) oligonucleotides 11/12 with pNCS‐mNeonGreen as template for pBH189 or with pQW58 as template for pEB21.2 and (iii) oligonucleotides 13/14 with pSTK3 as template. Secondly, the three PCR products and oligonucleotides 9/14 were used for the second amplification step. HindIII/AseI‐digested PCR products were cloned into correspondingly digested pBH4.

For construction of plasmids pBH500 and pBH501, a linker encoding SGSGSG, and the ORFs encoding mNeonGreen and mScarlet‐I, respectively, were fused with WALP23‐ORF by two‐step PCR. Briefly, for plasmid pBH500, two different PCR products were obtained using oligonucleotides 15/16 with pL030 as template and oligonucleotides 18/19 with pNCS‐mNeonGreen as template. For the second PCR step, oligonucleotides 15/19 were used. For plasmid pBH501, two PCR products were obtained using oligonucleotides 15/17 with pL030 as template and oligonucleotides 20/21 with synthesised mScarlet‐I‐encoding DNA as template. For the second PCR step, oligonucleotides 15/21 were used. In both cases, AvrII/SpeI‐digested PCR products were cloned into correspondingly digested pL030.

For construction of plasmids pJG130 and pJG131, plasmid pBAD322 was linearised with oligonucleotides 26 and 27. Full‐length *rne* was amplified using oligonucleotides 28/29, while inserts for construction of *rne*‐ΔAH were amplified using oligonucleotides 28/30 and 29/31. These fragments were fused using NEBuilder® HiFi DNA Assembly Cloning Kit (New England Biolabs).

All constructs were verified by DNA sequencing and are listed in Appendix Table S6. Oligonucleotides used are listed in Appendix Table S5.

### ***E***. ***coli* strains and growth conditions**

*E. coli* strains (Appendix Table S1) were grown in M9 minimal medium composed of Na_2_HPO_4_•2H_2_O (0.85% w/v), KH_2_PO_4_ (0.3% w/v), NaCl (0.3% w/v), NH_4_Cl (0.05% w/v), MgSO_4_•7H_2_O (0.25% w/v), CaCl_2_•2H_2_O (0.015% w/v) and supplemented with thiamine (0.01% w/v), casamino acids (0.1% w/v) and glucose (0.4% w/v) at 30°C, unless stated otherwise. For induction of the temperature‐sensitive phenotype *fabA*(Ts), pre‐cultures of *E. coli* strain UC1098 and its derivatives were diluted from an overnight culture grown at 30°C to OD_600_ of 0.025 and grown to OD_600_ of 0.5 at 30°C, and again diluted to OD_600_ of 0.025 in pre‐warmed, fresh medium for complete removal of cells in the stationary growth phase. At OD_600_ of 0.1–0.2, cells were transferred for 120 min to growth temperatures of 33, 37 or 40°C as indicated. WT strains were handled accordingly. For induction of the thermosensitive phenotype of *fabB15*(Ts), cells were grown essentially as described (Akamatsu, 1974). Precultures of *E. coli* strain BHH87 were diluted from an overnight culture, grown at 30°C in the presence or absence of 2% (w/v) KCl, to OD_600_ of 0.025 and grown at permissive 30°C to OD_600_ of 0.4–0.5 or grown at non‐permissive 40°C for 180 min, each with or without 2% KCl. The corresponding WT strain was handled accordingly. For recovery from UFA depletion and corresponding phase separation, UC1098 derivatives were subsequently supplemented with potassium oleate (100 µg/ml; Sigma‐Aldrich) dissolved in Brij®58 (0.1% w/v; Sigma‐Aldrich). Fluorescently labelled ATP synthase (F_O_F_1_ *a*‐mNG or F_O_F_1_ *a*‐mCherry; C‐terminal fusion to F_O_‐*a*), fluorescently labelled glucose permease (PtsG‐mNG; C‐terminal fusion) and msfGFP sandwich fusions of FtsZ and MreB were expressed from their own locus under control of their native promoter. Fluorescently labelled WALP23 (WALP23‐mScarlet‐I or WALP23‐mNG; amino acid sequence: AWW(LA)_8_LWWA) was expressed plasmid‐encoded under control of the *B. subtilis Pxyl* promoter *(*Appendix Table S6) resulting in constitutive expression in *E. coli*. Fluorescently labelled RNase E‐YFP (C‐terminal fusion) was expressed plasmid‐encoded under control of its own promoter. RNase E and ΔAH‐RNase E (RNase E lacking the membrane‐binding amphipathic helix) were expressed plasmid‐encoded under control of the *ParaBAD*‐inducible promoter.

### Construction of *B. subtilis* strains

All *B. subtilis* strains used are listed in Appendix Table S1. For construction of a *B. subtilis* strain expressing WALP23 fused to monomeric superfolder GFP (msfGFP), the plasmid pBH500 was linearised with oligonucleotides 22 and 23, msfGFP amplified using oligonucleotides 24 and 25 (Appendix Table S5) and the fragments fused using NEBuilder® HiFi DNA Assembly Cloning Kit (New England Biolabs). The resulting plasmid was transformed into *B. subtilis* 168, thus resulting in strain JG054. All other *B. subtilis* strains were constructed by transforming the respective recipient strains with chromosomal DNA from the donor strains or corresponding plasmid DNA. Transformations were carried out as described (Hamoen *et al*, 2002).

### *B*. *subtilis* strains and growth conditions

For strain construction, *B. subtilis* (Appendix Table S1) was grown either in LB (lysogeny broth), Nutrient Broth or Nutrient Agar (Oxoid). If necessary, these media were supplemented with either isobutyric acid (IB) (100 µM; Sigma‐Aldrich) or 2‐methylbutyric acid (MB) (100 µM; Sigma‐Aldrich). All other experiments were carried out with fortified Spizizen minimal medium composed of (NH_4_)_2_SO_4_ (0.2% w/v), K_2_HPO_4_ (1.4% w/v), KH_2_PO_4_ (0.6% w/v) Na_3_‐citrate•2H_2_O (0.1% w/v), MgSO_4_ (0.09% w/v), ferric ammonium citrate (1.1 µg/ml) supplemented with glucose (0.96% w/v), L‐tryptophan (20 µg/ml) and casamino acids (0.02% w/v). In our hands, the precursor isovaleric acid, which is the primer for the synthesis of *iso‐*C15:0 and *iso‐*C17:0, neither supported growth nor resulted in synthesis of the expected *iso‐*BCFAs, thus implying that this precursor cannot be supplied exogenously in the *B. subtilis* 168 strain background. All cultures were inoculated by 1:100 dilution of an LB overnight culture supplemented with the corresponding precursor. Depletion of BCFAs was carried out for cells initially grown in the presence of IB (100 µM), followed by washing, pelleting and resuspension in pre‐warmed, precursor‐free medium (PF). Unless stated otherwise, all experiments were carried out at 37°C. Fluorescently labelled WALP23 peptides (WALP23‐mCherry or WALP23‐msfGFP), msfGFP‐MreB and GFP‐FtsZ were expressed ectopically (*amyE* locus) under control of the *Pxyl* promoter and induced by xylose (1% w/v in case of WALP23; 0.3% w/v in case of GFP‐FtsZ).

### DPH anisotropy measurements

Steady‐state DPH fluorescent anisotropy measurements were carried out with 1,6‐Diphenyl‐1,3,5‐hexatriene (DPH)‐labelled cells using a BMG Clariostar multimode plate reader (BMG Labtech). For *B. subtilis*, cells taken from cultures at time points of interests were diluted to an OD_600_ of 0.25 in a pre‐warmed medium, followed by addition of DPH (Sigma Aldrich) dissolved in dimethyl formamide (DMF) to a final concentration of 10 µM DPH and 1% (v/v) DMF. Samples were shaken in dark at 37°C for 5 min, followed by a wash, resuspension in dye‐free medium to an OD_600_ of 0.5 and transfer to pre‐warmed, black, polystyrene 96‐well microtiter plates (Labsystems) for measurement. Following 1‐min incubation under shaking in the pre‐warmed plate reader to homogenise the sample, the fluorescence anisotropy was measured at 37°C using excitation wavelength of 360–10 nm, emission wavelength of 450–10 nm and a dichroic mirror set at 410 nm. The fluorescence anisotropy (A) was calculated with MARS Data Analysis software (BMG Labtech) using the equation (I_parallel_‐I_perpendicular_)/(I_parallel_+2xI_perpendicular_).

The corresponding measurements for *E. coli* were carried out using the same protocol with following modifications. Staining was carried out with cells grown in the presence of non‐growth inhibitory concentrations (30 µg/ml; Sigma‐Aldrich) of the outer membrane‐permeabilising agent Polymyxin B nonapeptide, which is required for good staining of *E. coli* with DPH. The measurements at 30 and 37°C without temperature shifts were carried out with all media, plastic ware and the plate reader pre‐warmed to the corresponding temperatures. The rapid temperature shift from 30 to 37°C was carried out with cells grown, stained and washed at 30°C, followed by final resuspension in buffer pre‐warmed to 37°C and measurement with microtiter plate as well as plate reader pre‐warmed to 37°C.

### Determination of fatty acid composition

The fatty acid composition of *E. coli* and *B. subtilis* was determined from 50 to 100 mg (wet weight) of bacterial cells grown as described in the main text. Fatty acids were extracted as methyl esters after saponification and methylation as described (Sasser, 1990). For saponification, cell pellets were mixed with 15% (w/v) NaOH in 50% (v/v) methanol (1 ml), incubated at 100°C for 5 min, vortexed and further incubated for 25 min. After cooling, acid methylation with 6 N HCl in 50% (v/v) methanol (2 ml) was performed for 10 min at 80°C followed by immediate cooling on ice. Methylated fatty acids were extracted by addition of hexane/methyl tert‐butyl ether in a 1:1 ratio (1.25 ml), followed by end‐over‐end incubation for 10 min. After phase separation by centrifugation, the lower phase was discarded. The organic phase was washed with 1.2% (w/v) NaOH (3 ml) by 5‐min end‐over‐end incubation and centrifugation. The upper phase of the phase‐separated sample was used for further analysis.

The fatty acid methyl esters (FAME) were separated and identified by gas chromatography‐mass spectrometry (GC‐MS) with a gas chromatograph (model 7890A; Agilent Technologies) equipped with a 5% phenylmethyl silicone capillary column and a mass spectrometer (model 5975C; Agilent Technologies). Helium was used as carrier gas, injection volume was 1 µl, injector temperature was 250°C, the column temperature was increased from 120 to 240°C at a rate of 5°C/min, and the GC‐MS line transfer temperature was 280°C. FAME were separated by their retention times and identified by their equivalent chain lengths and their mass spectra. Equivalent chain length values were calculated from linear interpolation of unknown peaks’ retention time between two saturated straight chain FAME of a standard.

For the analysis of the fatty acid composition of *B. subtilis* wild‐type cells grown at different temperatures (Appendix Fig S6), the cells were grown in LB medium and collected when the cultures reached an OD_600_ of approximately 0.5. The fatty acids were analysed as fatty acid methyl esters with GS‐MS as described above. However, these specific analyses were carried out by the Identification Service of the DSMZ, Braunschweig, Germany.

### Glycerophospholipid analysis by MALDI‐TOF mass spectrometry

Extraction of lipids from bacterial cells was performed according to Gidden *et al* (2009) as follows. 10^10^ cells (assuming that 1 ml of cell culture with OD_600_ of 1.0 contains 10^9^ cells (Neidhardt *et al*, 1990) were harvested, washed twice with cooled water and extracted with 450 µl of dichloromethane:ethanol:water 1:1:1 (v:v:v) overnight at 4°C. 1 μl of the lipid‐containing lower organic phase was spotted on a MALDI target plate (Prespotted AnchorChip 96 Set for Proteomics II; Bruker Daltonics/Eppendorf; washed peptide‐free with 2‐propanol) pre‐spotted with 1 µl of 9‐aminoacridine (10 mg/ml dissolved in acetone:water 9:1 (v/v)) and air‐dried. Mass spectra were obtained on an ultrafleXtreme MALDI‐TOF/TOF mass spectrometer equipped with a smartbeam™ solid‐state laser (Bruker Daltonics) operating in the negative ion mode. The laser was fired with a frequency of 500 Hz with 4 × 250 laser shots per spot. MS/MS spectra were obtained using the “LIFT” technique implemented in the mass spectrometer with an increased laser power. Samples from three independent biological replicates were measured per condition each as technical triplicates, compared with corresponding standard lipids PE (16:0) (18:1) and PG (16:0) (18:1) (Avanti polar lipids) in MS as well as MS/MS spectra and analysed with FlexAnalysis 3.4 (Bruker Daltonics).

### ONPG membrane permeability assay

For measuring passive ONPG membrane permeability in *E. coli* Δ*lacY* strains carrying plasmid‐encoded *lacZ*, overnight cultures were grown at 30°C in LB medium followed by 1:100 dilution in M9‐casamino acid medium and growth to an OD_600_ of 0.3. For maintaining the plasmid, media were supplemented with 50 µg/ml ampicillin. Cells at OD_600_ of 0.3 were transferred to clear 96‐well microtiter plates followed by addition of 2‐nitrophenyl β‐D‐galactopyranoside (ONPG) dissolved in PBS (Oxoid) to a final concentration of 2 mg/ml. LacZ‐driven hydrolysis of ONPG was monitored by measuring absorbance at 420 nm using a BMG Clariostar multimode plate reader, every 2 min for up to 30 min at 37°C with vigorous shaking in between. Meanwhile, the culture was transferred to 37°C for 120 min in a pre‐warmed water bath. Cells were then adjusted to OD_600_ 0.3 and ONPG measurements were repeated as above. The relative ONPG permeability was calculated as the rate of ONPG conversion, which is permeability‐limited in intact Δ*lacY* cells.

### Fluorescence microscopy

Regular wide‐field fluorescence microscopy was carried out with cells immobilised on Teflon‐coated multi‐spot microscope slides (Hendley‐Essex) with 1.2% (w/v) agarose/H_2_O (te Winkel *et al*, 2016). In brief, after the agarose solidified within 10 min at room temperature, 0.5 µl of cell culture was applied to the exposed agarose surface, air‐dried until the liquid‐drop was soaked in, covered with a coverslip and immediately used for microscopy. For staining with various fluorescent dyes, cells were incubated upon shaking at the growth temperature for 5 min with following concentrations: FM 5‐95 (2 µg/ml; Thermo Fisher Scientific), DiSC_3_(5) (2 µM; Sigma‐Aldrich), Sytox Green (50 ng/ml; Thermo Fisher Scientific) and DAPI (200 ng/ml; Sigma‐Aldrich). Membrane depolarisation of *B. subtilis* was achieved by 5‐min incubation with small cation specific channel‐forming antimicrobial peptide Gramicidin ABC (10 µM; gABC; Sigma‐Aldrich), and membrane permeabilisation by 5‐min incubation with pore‐forming lantibiotic Nisin (10 μM; Sigma‐Aldrich). In the case of *E. coli*, membrane depolarisation and permeabilisation were achieved by 15‐min incubation in the presence of pore‐forming antibiotic Polymyxin B (10 µg/ml; Sigma‐Aldrich), and nucleoid staining was achieved by 15‐min incubation with 500 ng/ml DAPI (Severn Biotech). Laurdan microscopy was carried out with cells stained with 100 µM Laurdan (Sigma‐Aldrich; dissolved in 1% (v/v) DMF) as described (Scheinpflug *et al*, 2017a; Wenzel *et al*, 2018). Staining of *E. coli* was carried out with Polymyxin B nonapeptide outer membrane‐permeabilised cells, as was done for DPH. The time lapse microscopy of *B. subtilis* was carried out with the fortified Spizizen minimal medium (with glucose, tryptophan and casamino acids) diluted to one tenth and supplemented with 1.4% (w/v) low‐melting point agarose. The slide preparation was carried out as described (de Jong *et al*, 2011).

The fluorescence microscopy of *B. subtilis* and *E. coli* cells stained with FM 5‐95, DiSC_3_(5), Sytox Green, Laurdan or expressing fluorescent protein fusions was performed at 37°C with Nikon Eclipse Ti equipped with either Sutter Instruments Lambda LS Xenon‐arc light source or CoolLed pE‐300white LED light source, CoolLed pE‐4000 LED light source, Photometrics Prime sCMOS camera, Photometrics BSI sCMOS camera and either Nikon Plan Fluor 100×/1.30 NA Oil Ph3, Nikon CFI Plan Apo VC 100×/1.40 NA or Nikon Plan Apo 100×/1.40 NA Oil Ph3 objectives. The used filter sets were Chroma 49000 (for DAPI and Laurdan 460 nm), Chroma 49002 (for GFP and Sytox Green), Chroma 49003 (for YFP), Chroma 49008 (for mCherry, mScarlet‐I and FM5‐95), Semrock Cy5‐4040C (for DiSC_3_(5)), a custom filter set consisting of Chroma AT350/50x excitation filter, Chroma T400lp beam splitter or Chroma ET525/50m (for Laurdan 520 nm). The microscopy images shown in Appendix Fig S7 were carried out with Applied Precision DeltaVision RT equipped with Photometrics Coolsnap HQ2 camera, Zeiss Plan‐APOCHROMAT 100× objective and the standard DeltaVision filters sets. Dual‐colour 2D‐SIM was carried out with Nikon N‐SIM equipped with Nikon CFI APO TIRF 100×/1.49 oil objective, 488 nm (Coherent Sapphire) and 561 nm (Cobolt Jive 100) solid‐state lasers and Andor Xion X3 EMCCD camera. The used filter sets were Chroma 49904 (for mNG) and 49909 (for mCherry). Image capture and reconstruction of high‐resolution 2D‐SIM images were performed with NIS elements 5.11 (Nikon). All images were analysed using Fiji (Schindelin *et al*, 2012). Laurdan GP maps were calculated and generated using the ImageJ‐macro as described (Wenzel *et al*, 2018). The localisation correlation analysis was carried out with the Fiji plugin Coloc 2, using a 3‐pixel wide line following the cell periphery as a region of interest.

For *E. coli fabA*(Ts), time lapse microscopy and widefield fluorescence microscopy microscope slides were coated with a thin film of 1% (w/v) agarose dissolved in M9 minimal media supplemented with glucose/casamino acids. Cells (1 or 3 µl) were immobilised and imaged with a DeltaVision Elite microscopy system (Applied Precision, GE Healthcare) equipped with an inverted microscope (IX‐71, Olympus), a 100× oil immersion objective (UAPON 100x TIRF, Olympus) or an extended apochromat phase‐contrast objective (UPLXAPO100XOPH, Olympus), solid‐state illumination system (Insight SSI, Applied Precision), a sCMOS camera (pco.edge 4.2, PCO) and acquisition software (softWoRx 5.5, Applied Precision). *Z*‐Stacks of 300 nm in 5 optical slices were acquired of cells growing on the microscope slide with the microscope tempered to the corresponding temperature and imaged for 2.5 h with 5‐min intervals using an exposure time per frame of 50 ms. Fluorescence of mNeonGreen and mScarlet‐I was excited using a polychromic beamsplitter (405 nm/488 nm/590 nm/650 nm) as well as either a GFP/FITC bandpass filter (461–489 nm) or a mCherry/Alexa594 bandpass filter (562–588 nm). Fluorescence detection was achieved using a GFP/FITC bandpass emission filter (501–559 nm) for mNG and a mCherry/Alexa594 bandpass emission filter (602–648 nm) for mScarlet‐I.

Single molecule imaging of *E. coli* cells expressing F_O_F_1_ *a*‐mNG or WALP23‐mNG was performed using a total internal reflection fluorescence (TIRF) microscopy system equipped with an inverted microscope (IX‐83, Olympus), a motorised four‐line TIRF condenser (cellTIRF, Olympus), an 150× oil immersion objective (UAPON 150×/1.45 NA TIRF, Olympus), an EMCCD camera (iXON Ultra 897, Andor) and the acquisition software CellSens 2.3 (Olympus). Fluorescence of mNG was excited by a 488 nm laser diode (LuxX 488‐200, Omicron) using a TIRF pentaband polychroic mirror (zt405/488/561/640/730rpc, Chroma). Fluorescence detection and efficient TIRF laser blocking were achieved by a pentabandpass emission filter (BrightLine HC 440/521/607/694/809, Semrock) and an additional single bandpass emission filter (BrightLine HC 525/35, Semrock). All images were analysed using Fiji (Schindelin *et al*, 2012).

### Single molecule tracking

Single molecule imaging of *E. coli* cells expressing F_O_F_1_ *a*‐mNG was performed using a total internal reflection fluorescence (TIRF) microscope. Cells were pre‐bleached for 2.5 s at 10% laser intensity (approx. 1.5 µW/µm^2^) to obtain single molecule fluorescence level. Subsequently, single emitter signals were imaged at 30 frames per second for 1,200 frames (40 s) with 5% of laser intensity. All experiments were carried out at room temperature for comparability. Tracking of single molecules and data analysis were carried out with well‐established localisation and tracking algorithms, implemented in a software package called “SLIMfast” (kindly provided by C.P. Richter (Osnabrück)) written in Matlab (Richter *et al*, 2017; Appelhans *et al*, 2018). Localisation precision was typically about 20–25 nm. Between 900 and 1,000 frames per image series were used for further step length and diffusion constant analysis. Step length analysis is based on trajectories exhibiting at least five sequential frames (excluding deflation loops and frame gaps). Typically, the population of all trajectories (step size of ≥ 1) is approximately twice as high as those taken into account (step size of ≥ 5).

Analysis of lateral mobility was performed via cumulative probability plots with jump distances of pooled trajectories from 3 to 5 separately grown cell batches and via boxplots to determine the median of all trajectories present within individual cells. Apparent two‐dimensional diffusion coefficients *D_app_* were estimated by the mean‐squared displacement (MSD) $<{\left(\Delta r\left(\tau \right)\right)}^{2}>=4D_{\mathrm{app}}\tau$ considering a linear free diffusion model. Here, τ=Δt,2Δt,…,nΔt is the lag time defined by multiples of time interval Δ*t* of the image series. For all trajectories with at least ≥5 five sequential frames, MSD was averaged and the diffusion coefficient calculated by the slope of a linear fit based on the first four data points of the MSD. For the determination of standard error, the statistical resampling method of bootstrapping was used, evaluating data sets with N data points 1,000 times (Bradley, 1981).

### Membrane vesicles and DCCD‐sensitive ATPase activity

Inverted membrane vesicles were prepared as previously described (Brandt *et al*, 2013) using 850 ml of cell culture grown in M9 minimal medium with 0.4% (w/v) glucose and 0.1% (w/v) casamino acids. After harvest, cell pellets were resuspended in 25 ml 50 mM Tris–HCl, pH 7.5, 10 mM MgCl_2_, 10% (v/v) glycerol and disrupted in the presence of 10 µg/ml DNaseI (Sigma) with a constant cell disruptor system (Daventry) at 4°C and 1.35 kbar. For removal of cell debris, lysates were centrifuged at 35,000 *g* for 30 min at 4°C. After ultracentrifugation of the supernatant at 250,000 *g* at 4°C for 60 min, membranes were resuspended with a marten paint brush in a small aliquot (0.3–0.5 ml) of the same buffer and stored in liquid nitrogen.

ATPase activities of inverted membrane vesicles were determined using an automated continuous assay enabling a direct recording of the substrate turnover (Arnold *et al*, 1976). For inhibition of ATPase activities with 80 µM N,*N*′‐dicyclohexylcarbodiimide (DCCD; Sigma‐Aldrich; stock solution 40 mM in ethanol), membranes were incubated in 1 ml of 50 mM Tris–HCl, pH 8.0 for 20 min at 37°C prior to measurement (Deckers‐Hebestreit & Altendorf, 1992).

### SDS–PAGE and Western blot

Protein concentrations were determined with the BCA assay as recommended by the supplier (Pierce). Proteins (20 µg/lane) were separated by SDS–PAGE using 10% Tris‐Tricine gels (10% T, 3% C) (Schägger & von Jagow, 1987) with PageRuler^TM^ prestained protein ladder (Fermentas) as standard. For immunoblotting, separated proteins were transferred to nitrocellulose membranes (0.45 μm) via wet blotting in carbonate buffer (10 mM NaHCO_3_, 3 mM Na_2_CO_3_, pH 8.9 with NaOH, 20% (v/v) methanol) for 45 min at 1.8 A with cooling (Hilbers *et al*, 2013). Membranes were blocked with 5% (w/v) skimmed milk powder in TBS buffer (50 mM Tris–HCl pH 7.4, 0.9% (w/v) NaCl), decorated with monoclonal mouse antibodies specific for F_O_‐*a* (GDH 14‐5C6 (Jäger *et al*, 1998)) or mNeonGreen (32F6, ChromoTek) and secondary IRDye^™^800DX‐labelled goat‐anti‐mouse IgG (H + L) (LI‐COR Biosciences), and detected using a two‐channel Odyssey infrared imaging System (LI‐COR Biosciences).

### Quantification and statistical analysis

Statistical analysis was performed using a two‐sided Wilcoxon rank sum test (MATLAB) or an unpaired two‐sided *t*‐test (GraphPad Prism or Microsoft Excel). Error bars represent SD from three independent biological replicates, unless stated otherwise. Significance was assumed with ****P < 0.0001, ***P < 0.001, **P < 0.01, *P < 0.05, n.s., not significant.

## Author contributions

GD‐H and HS designed research; GD‐H and HS coordinated the collaborative research; MG, AL, JWG, JAB, BH, ZB and HS performed the experiments; MG, AL, JWG, JAB, RK, SW, GD‐H and HS analysed data; HS, GD‐H and MG wrote the paper.

## Supporting information



Appendix

Expanded View Figures PDF

Movie EV1

Movie EV2

Movie EV3

Movie EV4

Source Data for Expanded View and Appendix

Source Data for Figure 1

Source Data for Figure 2

Source Data for Figure 3

Source Data for Figure 6

Source Data for Figure 7

## Data Availability

Additional figures and tables can be found in the Appendix. Correspondence and requests for material should be addressed to H.S. or G.D.‐H. Source data are available online for individual graphs. This study includes no data deposited in external repositories.

## References

[embj2021109800-bib-0001] Adams DW , Errington J (2009) Bacterial cell division: assembly, maintenance and disassembly of the Z ring. Nat Rev Microbiol 7: 642–653 19680248 10.1038/nrmicro2198

[embj2021109800-bib-0002] Akamatsu Y (1974) Osmotic stabilization of unsaturated fatty acid auxotrophs of *Escherichia coli* . J Biochem 76: 553–561 4612031 10.1093/oxfordjournals.jbchem.a130599

[embj2021109800-bib-0003] Andersen OS , Koeppe 2nd RE (2007) Bilayer thickness and membrane protein function: an energetic perspective. Annu Rev Biophys Biomol Struct 36: 107–130 17263662 10.1146/annurev.biophys.36.040306.132643

[embj2021109800-bib-0004] Appelhans T , Beinlich FRM , Richter CP , Kurre R , Busch KB (2018) Multi‐color localisation microscopy of single membrane proteins in organelles of live mammalian cells. J Vis Exp 136: e57690 10.3791/57690 PMC6102026 30010642

[embj2021109800-bib-0005] Arnold A , Wolf HU , Ackermann BP , Bader H (1976) An automated continuous assay of membrane‐bound and soluble ATPases and related enzymes. Anal Biochem 71: 209–213 179440 10.1016/0003-2697(76)90029-4

[embj2021109800-bib-0006] Ballweg S , Sezgin E , Doktorova M , Covino R , Reinhard J , Wunnicke D , Hänelt I , Levental I , Hummer G , Ernst R (2020) Regulation of lipid saturation without sensing membrane fluidity. Nat Commun 11: 756 32029718 10.1038/s41467-020-14528-1 PMC7005026

[embj2021109800-bib-0007] Baumgart T , Hammond AT , Sengupta P , Hess ST , Holowka DA , Baird BA , Webb WW (2007) Large‐scale fluid/fluid phase separation of proteins and lipids in giant plasma membrane vesicles. Proc Natl Acad Sci USA 104: 3165–3170 17360623 10.1073/pnas.0611357104 PMC1805587

[embj2021109800-bib-0008] Bigay J , Antonny B (2012) Curvature, lipid packing, and electrostatics of membrane organelles: defining cellular territories in determining specificity. Dev Cell 23: 886–895 23153485 10.1016/j.devcel.2012.10.009

[embj2021109800-bib-0009] Boudreaux DP , Eisenstadt E , Iijima T , Freese E (1981) Biochemical and genetic characterization of an auxotroph of *Bacillus subtilis* altered in the acyl‐CoA:acyl‐carrier‐protein transacylase. Eur J Biochem 115: 175–181 6785086 10.1111/j.1432-1033.1981.tb06214.x

[embj2021109800-bib-0010] Bradley E (1981) Nonparametric estimates of standard error: the jackknife, the bootstrap and other methods. Biometrika 68: 589–599

[embj2021109800-bib-0011] Brandt K , Maiwald S , Herkenhoff‐Hesselmann B , Gnirß K , Greie JC , Dunn SD , Deckers‐Hebestreit G (2013) Individual interactions of the *b* subunits within the stator of the *Escherichia coli* ATP synthase. J Biol Chem 288: 24465–24479 23846684 10.1074/jbc.M113.465633 PMC3750146

[embj2021109800-bib-0012] Broekman JH , Steenbakkers JF (1973) Growth in high osmotic medium of an unsaturated fatty acid auxotroph of *Escherichia coli* K‐12. J Bacteriol 116: 285–289 4583215 10.1128/jb.116.1.285-289.1973 PMC246420

[embj2021109800-bib-0013] Broekman JH , Steenbakkers JF (1974) Effect of the osmotic pressure of the growth medium on *fabB* mutants of *Escherichia coli* . J Bacteriol 117: 971–977 4591962 10.1128/jb.117.3.971-977.1974 PMC246574

[embj2021109800-bib-0014] Budin I , de Rond T , Chen Y , Chan LJG , Petzold CJ , Keasling JD (2018) Viscous control of cellular respiration by membrane lipid composition. Science 362: 1186–1189 30361388 10.1126/science.aat7925

[embj2021109800-bib-0015] Burns M , Wisser K , Wu J , Levental I , Veatch SL (2017) Miscibility transition temperature scales with growth temperature in a zebrafish cell line. Biophys J 113: 1212–1222 28552311 10.1016/j.bpj.2017.04.052 PMC5607031

[embj2021109800-bib-0016] Chapman D (1975) Phase transitions and fluidity characteristics of lipids and cell membranes. Q Rev Biophys 8: 185–235 1103214 10.1017/s0033583500001797

[embj2021109800-bib-0017] Chen IA , Walde P (2010) From self‐assembled vesicles to protocells. Cold Spring Harb Perspect Biol 2: a002170 20519344 10.1101/cshperspect.a002170 PMC2890201

[embj2021109800-bib-0018] Cordeiro RM (2018) Molecular structure and permeability at the interface between phase‐separated membrane domains. J Phys Chem B 122: 6954–6965 29767519 10.1021/acs.jpcb.8b03406

[embj2021109800-bib-0019] Cronan Jr JE , Gelmann EP (1973) An estimate of the minimum amount of unsaturated fatty acid required for growth of *Escherichia coli* . J Biol Chem 248: 1188–1195 4568811

[embj2021109800-bib-0020] Datsenko KA , Wanner BL (2000) One‐step inactivation of chromosomal genes in *Escherichia coli* K‐12 using PCR products. Proc Natl Acad Sci USA 97: 6640–6645 10829079 10.1073/pnas.120163297 PMC18686

[embj2021109800-bib-0021] Debarbouille M , Gardan R , Arnaud M , Rapoport G (1999) Role of BkdR, a transcriptional activator of the SigL‐dependent isoleucine and valine degradation pathway in *Bacillus subtilis* . J Bacteriol 181: 2059–2066 10094682 10.1128/jb.181.7.2059-2066.1999 PMC93617

[embj2021109800-bib-0022] Deckers‐Hebestreit G , Altendorf K (1992) Influence of subunit‐specific antibodies on the activity of the F_O_ complex of the ATP synthase of *Escherichia coli*. II. Effects of subunit *c*‐specific polyclonal antibodies. J Biol Chem 267: 12370–12374 1376323

[embj2021109800-bib-0023] Diomandé SE , Nguyen‐The C , Guinebretière MH , Broussolle V , Brillard J (2015) Role of fatty acids in *Bacillus* environmental adaptation. Front Microbiol 6: 813 26300876 10.3389/fmicb.2015.00813 PMC4525379

[embj2021109800-bib-0024] Domański J , Marrink SJ , Schäfer LV (2012) Transmembrane helices can induce domain formation in crowded model membranes. Biochim Biophys Acta 1818: 984–994 21884678 10.1016/j.bbamem.2011.08.021

[embj2021109800-bib-0025] Drin G , Antonny B (2010) Amphipathic helices and membrane curvature. FEBS Lett 584: 1840–1847 19837069 10.1016/j.febslet.2009.10.022

[embj2021109800-bib-0026] Drobnis EZ , Crowe LM , Berger T , Anchordoguy TJ , Overstreet JW , Crowe JH (1993) Cold shock damage is due to lipid phase transitions in cell membranes: a demonstration using sperm as a model. J Exp Zool 265: 432–437 8463792 10.1002/jez.1402650413

[embj2021109800-bib-0027] Elson EL , Fried E , Dolbow JE , Genin GM (2010) Phase separation in biological membranes: integration of theory and experiment. Annu Rev Biophys 39: 207–226 20192775 10.1146/annurev.biophys.093008.131238 PMC3694198

[embj2021109800-bib-0028] Ernst R , Ballweg S , Levental I (2018) Cellular mechanisms of physicochemical membrane homeostasis. Curr Op Cell Biol 53: 44–51 29787971 10.1016/j.ceb.2018.04.013 PMC6131038

[embj2021109800-bib-0029] Ernst R , Ejsing CS , Antonny B (2016) Homeoviscous adaptation and the regulation of membrane lipids. J Mol Biol 428: 4776–4791 27534816 10.1016/j.jmb.2016.08.013

[embj2021109800-bib-0030] Galli E , Gerdes K (2010) Spatial resolution of two bacterial cell division proteins: ZapA recruits ZapB to the inner face of the Z‐ring. Mol Microbiol 76: 1514–1526 20487275 10.1111/j.1365-2958.2010.07183.x

[embj2021109800-bib-0031] Ghetler Y , Yavin S , Shalgi R , Arav A (2005) The effect of chilling on membrane lipid phase transition in human oocytes and zygotes. Hum Reprod 20: 3385–3389 16055458 10.1093/humrep/dei236

[embj2021109800-bib-0032] Gidden J , Denson J , Liyanage R , Ivey DM , Lay JO (2009) Lipid compositions in *Escherichia coli* and *Bacillus subtilis* during growth as determined by MALDI‐TOF and TOF/TOF mass spectrometry. Int J Mass Spectrom 283: 178–184 20161304 10.1016/j.ijms.2009.03.005 PMC2699300

[embj2021109800-bib-0033] Hamoen LW , Smits WK , de Jong A , Holsappel S , Kuipers OP (2002) Improving the predictive value of the competence transcription factor (ComK) binding site in *Bacillus subtilis* using a genomic approach. Nucl Acids Res 30: 5517–5528 12490720 10.1093/nar/gkf698 PMC140081

[embj2021109800-bib-0034] Harayama T , Riezman H (2018) Understanding the diversity of membrane lipid composition. Nat Rev Mol Cell Biol 19: 281–296 29410529 10.1038/nrm.2017.138

[embj2021109800-bib-0035] Hazel JR (1995) Thermal adaptation in biological membranes: Is homeoviscous adaptation the explanation? Annu Rev Physiol 57: 19–42 7778864 10.1146/annurev.ph.57.030195.000315

[embj2021109800-bib-0036] Heberle FA , Feigenson GW (2011) Phase separation in lipid membranes. Cold Spring Harb Perspect Biol 3: a004630 21441593 10.1101/cshperspect.a004630 PMC3062215

[embj2021109800-bib-0037] Heimburg T (2007) Thermal biophysics of membranes. Weinheim, Germany: Wiley‐VCH Verlag

[embj2021109800-bib-0038] Hilbers F , Eggers R , Pradela K , Friedrich K , Herkenhoff‐Hesselmann B , Becker E , Deckers‐Hebestreit G (2013) Subunit δ is the key player for assembly of the H^+^‐translocating unit of *Escherichia coli* F_O_F_1_ ATP synthase. J Biol Chem 288: 25880–25894 23864656 10.1074/jbc.M113.484675 PMC3764793

[embj2021109800-bib-0039] Hu Z , Mukherjee A , Pichoff S , Lutkenhaus J (1999) The MinC component of the division site selection system in *Escherichia coli* interacts with FtsZ to prevent polymerization. Proc Natl Acad Sci USA 96: 14819–14824 10611296 10.1073/pnas.96.26.14819 PMC24731

[embj2021109800-bib-0040] Jäger H , Birkenhäger R , Stalz WD , Altendorf K , Deckers‐Hebestreit G (1998) Topology of subunit *a* of the *Escherichia coli* ATP synthase. Eur J Biochem 251: 122–132 9492276 10.1046/j.1432-1327.1998.2510122.x

[embj2021109800-bib-0041] de Jong IG , Beilharz K , Kuipers OP , Veening JW (2011) Live cell imaging of *Bacillus subtilis* and *Streptococcus pneumoniae* using automated time‐lapse microscopy. J Vis Exp 53: e3145 10.3791/3145 PMC3197447 21841760

[embj2021109800-bib-0042] Junge W , Nelson N (2015) ATP synthase. Annu Rev Biochem 84: 631–657 25839341 10.1146/annurev-biochem-060614-034124

[embj2021109800-bib-0043] Kaneda T (1977) Fatty acids of the genus *Bacillus*: an example of branched‐chain preference. Bacteriol Rev 41: 391–418 329832 10.1128/br.41.2.391-418.1977 PMC414006

[embj2021109800-bib-0044] Klein W , Weber MHW , Marahiel MA (1999) Cold shock response of *Bacillus subtilis*: Isoleucine‐dependent switch in the fatty acid branching pattern for membrane adaptation to low temperatures. J Bacteriol 181: 5341–5349 10464205 10.1128/jb.181.17.5341-5349.1999 PMC94040

[embj2021109800-bib-0045] Köhler P , Marahiel MA (1997) Association of the histone‐like protein HBsu with the nucleoid of *Bacillus subtilis* . J Bacteriol 179: 2060–2064 9068655 10.1128/jb.179.6.2060-2064.1997 PMC178933

[embj2021109800-bib-0046] Kosfeld A , Jahreis K (2012) Characterization of the interaction between the small regulatory peptide SgrT and the EIICBGlc of the glucose‐phosphotransferase system of *E. coli* K‐12. Metabolites 2: 756–774 24957761 10.3390/metabo2040756 PMC3901232

[embj2021109800-bib-0047] Kumar M , Mommer MS , Sourjik V (2010) Mobility of cytoplasmic, membrane, and DNA‐binding proteins in *Escherichia coli* . Biophys J 98: 552–559 20159151 10.1016/j.bpj.2009.11.002 PMC2820653

[embj2021109800-bib-0048] Leake MC , Greene NP , Godun RM , Granjon T , Buchanan G , Chen S , Berry RM , Palmer T , Berks BC (2008) Variable stoichiometry of the TatA component of the twin‐arginine protein transport system observed by *in vivo* single‐molecule imaging. Proc Natl Acad Sci USA 105: 15376–15381 18832162 10.1073/pnas.0806338105 PMC2563114

[embj2021109800-bib-0049] Lee AG (2004) How lipids affect the activities of integral membrane proteins. Biochim Biophys Acta 1666: 62–87 15519309 10.1016/j.bbamem.2004.05.012

[embj2021109800-bib-0050] Lenaz G (1987) Lipid fluidity and membrane protein dynamics. Biosci Rep 7: 823–837 3329533 10.1007/BF01119473

[embj2021109800-bib-0051] Lentz BR (1993) Use of fluorescent probes to monitor molecular order and motions within liposome bilayers. Chem Phys Lipids 64: 99–116 8242843 10.1016/0009-3084(93)90060-g

[embj2021109800-bib-0052] Letellier L , Moudden H , Shechter E (1977) Lipid and protein segregation in *Escherichia coli* membrane: Morphological and structural study of different cytoplasmic membrane fractions. Proc Natl Acad Sci USA 74: 452–456 322126 10.1073/pnas.74.2.452 PMC392307

[embj2021109800-bib-0053] Levental KR , Malmberg E , Symons JL , Fan YY , Chapkin RS , Ernst R , Levental I (2020) Lipidomic and biophysical homeostasis of mammalian membranes counteracts dietary lipid perturbations to maintain cellular fitness. Nat Commun 11: 1339 32165635 10.1038/s41467-020-15203-1 PMC7067841

[embj2021109800-bib-0054] Lewis RNAH , Sykes BD , McElhaney RN (1987) Thermotropic phase behavior of model membranes composed of phosphatidylcholines containing *dl*‐methyl anteisobranched fatty acids. 1. Differential scanning calorimetric and ^31^P NMR spectroscopic studies. Biochemistry 26: 4036–4044 3651434 10.1021/bi00387a044

[embj2021109800-bib-0055] Lingwood D , Simons K (2010) Lipid rafts as a membrane‐organizing principle. Science 327: 46–50 20044567 10.1126/science.1174621

[embj2021109800-bib-0056] Lorent JH , Diaz‐Rohrer B , Lin X , Spring K , Gorfe AA , Levental KR , Levental I (2017) Structural determinants and functional consequences of protein affinity for membrane rafts. Nat Commun 8: 1219 29089556 10.1038/s41467-017-01328-3 PMC5663905

[embj2021109800-bib-0057] Lucena D , Mauri M , Schmidt F , Eckhardt B , Graumann PL (2018) Microdomain formation is a general property of bacterial membrane proteins and induces heterogeneity of diffusion patterns. BMC Biol 16: 97 30173665 10.1186/s12915-018-0561-0 PMC6120080

[embj2021109800-bib-0058] Mansilla MC , Cybulski LE , Albanesi D , de Mendoza D (2004) Control of membrane lipid fluidity by molecular thermosensors. J Bacteriol 186: 6681–6688 15466018 10.1128/JB.186.20.6681-6688.2004 PMC522199

[embj2021109800-bib-0059] Marr AG , Ingraham JL (1962) Effect of temperature on the composition of fatty acids in *Escherichia coli* . J Bacteriol 84: 1260–1267 16561982 10.1128/jb.84.6.1260-1267.1962 PMC278056

[embj2021109800-bib-0060] Morein S , Andersson AS , Rilfors A , Lindblom G (1996) Wild‐type *Escherichia coli* regulate the membrane lipid composition in a “window” between gel and non‐lamellar structures. J Biol Chem 271: 6801–6809 8636103 10.1074/jbc.271.12.6801

[embj2021109800-bib-0061] Mostofian B , Zhuang T , Cheng X , Nickels JD (2019) Branched‐chain fatty acid content modulates structure, fluidity, and phase in model microbial cell membranes. J Phys Chem B 123: 5814–5821 31251616 10.1021/acs.jpcb.9b04326

[embj2021109800-bib-0062] Neidhardt FC , Ingraham JL , Schaechter M (1990) Physiology of the bacterial cell. A molecular approach. Sunderland, MA: Sinauer Associates

[embj2021109800-bib-0063] Nguyen C , Haushalter RW , Lee DJ , Markwick PRL , Bruegger J , Caldara‐Festin G , Finzel K , Jackson DR , Ishikawa F , O’Dowd B *et al* (2014) Trapping the dynamic acyl carrier protein in fatty acid biosynthesis. Nature 505: 427–431 24362570 10.1038/nature12810 PMC4437705

[embj2021109800-bib-0064] Nickels JD , Chatterjee S , Mostofian B , Stanley CB , Ohl M , Zolnierczuk P , Schulz R , Myles DAA , Standaert RF , Elkins JG *et al* (2017) *Bacillus subtilis* lipid extract, a branched‐chain fatty acid model membrane. J Phys Chem Lett 8: 4214–4217 28825491 10.1021/acs.jpclett.7b01877

[embj2021109800-bib-0065] Nickels JD , Hogg J , Cordner D , Katsaras J (2019) Lipid rafts in bacteria: structure and function. In Health consequences of microbial interactions with hydrocarbons, oil, and lipids, Goldfine H (ed.), pp 1–30. Cham, Switzerland: Springer Nature

[embj2021109800-bib-0066] Nicolson GL (2014) The fluid‐mosaic model of membrane structure: Still relevant to understanding the structure, function and dynamics of biological membranes after more than 40 years. Biochim Biophys Acta 1838: 1451–1466 24189436 10.1016/j.bbamem.2013.10.019

[embj2021109800-bib-0067] Oswald F , Varadarajan A , Lill H , Peterman EJG , Bollen YEM (2016) MreB‐dependent organization of the *E. coli* cytoplasmic membrane controls membrane protein diffusion. Biophys J 110: 1139–1149 26958890 10.1016/j.bpj.2016.01.010 PMC4788719

[embj2021109800-bib-0068] Papahadjopoulos D , Jacobson K , Nir S , Isac T (1973) Phase transitions in phospholipid vesicles. Fluorescence polarization and permeability measurements concerning the effect of temperature and cholesterol. Biochim Biophys Acta 311: 330–348 4729825 10.1016/0005-2736(73)90314-3

[embj2021109800-bib-0069] Parasassi T , De Stasio G , d'Ubaldo A , Gratton E (1990) Phase fluctuation in phospholipid membranes revealed by Laurdan fluorescence. Biophys J 57: 1179–1186 2393703 10.1016/S0006-3495(90)82637-0 PMC1280828

[embj2021109800-bib-0070] Parsons JB , Rock CO (2013) Bacterial lipids: metabolism and membrane homeostasis. Prog Lipid Res 52: 249–276 23500459 10.1016/j.plipres.2013.02.002 PMC3665635

[embj2021109800-bib-0071] Picas L , Carreterro‐Genevrier A , Montero MT , Vázquez‐Ibar JL , Seantier B , Milhiet PE , Hernández‐Borrell J (2010) Preferential insertion of lactose permease in phospholipid domains: AFM observations. Biochim Biophys Acta 1798: 1014–1019 20096263 10.1016/j.bbamem.2010.01.008

[embj2021109800-bib-0072] Pilizota T , Shaevitz JW (2012) Fast, multiphase volume adaptation to hyperosmotic shock by *Escherichia coli* . PLoS One 7: e35205 22514721 10.1371/journal.pone.0035205 PMC3325977

[embj2021109800-bib-0073] Ramadurai S , Holt A , Krasnikov V , van den Bogaart G , Killian JA , Poolman B (2009) Lateral diffusion of membrane proteins. J Am Chem Soc 131: 12650–12656 19673517 10.1021/ja902853g

[embj2021109800-bib-0074] Razin S (1967) The cell membrane of mycoplasma. Ann NY Acad Sci 143: 115–129 4228218 10.1111/j.1749-6632.1967.tb27651.x

[embj2021109800-bib-0075] Renz A , Renz M , Klütsch D , Deckers‐Hebestreit G , Börsch M (2015) 3D‐localisation microscopy and tracking of F_O_F_1_‐ATP synthases in living bacteria. Proc SPIE 9331: 93310D

[embj2021109800-bib-0076] Richter D , Moraga I , Winkelmann H , Birkholz O , Wilmes S , Schulte M , Kraich M , Kenneweg H , Beutel O , Selenschik P *et al* (2017) Ligand‐induced type II interleukin‐4 receptor dimers are sustained by rapid re‐association within plasma membrane microcompartments. Nat Commun 8: 15976 28706306 10.1038/ncomms15976 PMC5519985

[embj2021109800-bib-0077] Ridder ANJA , Spelbrink REJ , Demmers JAA , Rijkers DTS , Liskamp RMJ , Brunner J , Heck AJR , de Kruijff B , Killian JA (2004) Photo‐crosslinking analysis of preferential interactions between a transmembrane peptide and matching lipids. Biochemistry 43: 4482–4489 15078094 10.1021/bi049899d

[embj2021109800-bib-0078] Rock CO , Tsay JT , Heath R , Jackowski S (1996) Increased unsaturated fatty acid production associated with a suppressor of the *fabA6*(Ts) mutation in *Escherichia coli* . J Bacteriol 178: 5382–5387 8808925 10.1128/jb.178.18.5382-5387.1996 PMC178354

[embj2021109800-bib-0079] Rossignol M , Thomas P , Grignon C (1982) Proton permeability of liposomes from natural phospholipid mixtures. Biochim Biophys Acta 684: 195–199 7055561 10.1016/0005-2736(82)90005-0

[embj2021109800-bib-0080] Roth BL , Poot M , Yue ST , Millard PJ (1997) Bacterial viability and antibiotic susceptibility testing with SYTOX green nucleic acid stain. Appl Environ Microbiol 63: 2421–2431 9172364 10.1128/aem.63.6.2421-2431.1997 PMC168536

[embj2021109800-bib-0081] Rudner DZ , Pan Q , Losick RM (2002) Evidence that subcellular localization of a bacterial membrane protein is achieved by diffusion and capture. Proc Natl Acad Sci USA 99: 8701–8706 12060714 10.1073/pnas.132235899 PMC124362

[embj2021109800-bib-0082] Sáenz JP , Grosser D , Bradley AS , Lagny TJ , Lavrynenko O , Broda M , Simons K (2015) Hopanoids as functional analogues of cholesterol in bacterial membranes. Proc Natl Acad Sci USA 112: 11971–11976 26351677 10.1073/pnas.1515607112 PMC4586864

[embj2021109800-bib-0083] Sasser M (1990) Identification of bacteria through fatty acid analysis. In Methods in phyto‐bacteriology 595, Klement Z , Rudolph K , Sands DC (eds.), Hungary: Akadémiai Kiadó Budapest

[embj2021109800-bib-0084] Schäfer LV , de Jong DH , Holt A , Rzepiela AJ , de Vries AH , Poolman B , Killian JA , Marrink SJ (2011) Lipid packing drives the segregation of transmembrane helices into disordered lipid domains in model membranes. Proc Natl Acad Sci USA 108: 1343–1348 21205902 10.1073/pnas.1009362108 PMC3029762

[embj2021109800-bib-0085] Schägger H , von Jagow G (1987) Tricine‐sodium dodecyl sulfate polyacrylamide gel electrophoresis for the separation of proteins in the range of 1 to 100 kDa. Anal Biochem 166: 368–379 Proc Natl Acad Sci USA 97: 6640–6645 2449095 10.1016/0003-2697(87)90587-2

[embj2021109800-bib-0086] Scheinpflug K , Krylova O , Strahl H (2017a) Measurement of cell membrane fluidity by Laurdan GP: fluorescence spectroscopy and microscopy. Methods Mol Biol 1520: 159–174 27873252 10.1007/978-1-4939-6634-9_10

[embj2021109800-bib-0087] Scheinpflug K , Wenzel M , Krylova O , Bandow JE , Dathe M , Strahl H (2017b) Antimicrobial peptide cWFW kills by combining lipid phase separation with autolysis. Sci Rep 7: 44332 28276520 10.1038/srep44332 PMC5343580

[embj2021109800-bib-0088] Schindelin J , Arganda‐Carreras I , Frise E , Kaynig V , Longair M , Pietzsch T , Preibisch S , Rueden C , Saalfeld S , Schmid B *et al* (2012) Fiji: an open‐source platform for biological‐image analysis. Nat Methods 9: 676–682 22743772 10.1038/nmeth.2019 PMC3855844

[embj2021109800-bib-0089] Schmid F (2017) Physical mechanisms of micro‐ and nanodomain formation in multicomponent lipid membranes. Biochim Biophys Acta 1859: 509–528 10.1016/j.bbamem.2016.10.021 27823927

[embj2021109800-bib-0090] Sharp MD , Pogliano K (1999) An *in vivo* membrane fusion assay implicates SpoIIIE in the final stages of engulfment during *Bacillus subtilis* sporulation. Proc Natl Acad Sci USA 96: 14553–14558 10588743 10.1073/pnas.96.25.14553 PMC24474

[embj2021109800-bib-0091] Shaw TR , Ghosh S , Veatch SL (2021) Critical phenomena in plasma membrane organization and function. Annu Rev Phys Chem 72: 51–72 33710910 10.1146/annurev-physchem-090419-115951 PMC8170621

[embj2021109800-bib-0092] Shelby SA , Castello‐Serrano I , Wisser KC , Levental I , Veatch SL (2021) Membrane phase separation drives organization at B cell receptor clusters. *bioRxiv* 10.1101/2021.05.12.443834 [PREPRINT] PMC10771812 36997644

[embj2021109800-bib-0093] Shen Y , Zhao Z , Zhang L , Shi L , Shahriar S , Chan RB , Di Paolo G , Min W (2017) Metabolic activity induces membrane phase separation in endoplasmic reticulum. Proc Natl Acad Sci USA 114: 13394–13399 29196526 10.1073/pnas.1712555114 PMC5754785

[embj2021109800-bib-0094] Sinensky M (1974) Homeoviscous adaptation ‐ a homeostatic process that regulates the viscosity of membrane lipids in *Escherichia coli* . Proc Natl Acad Sci USA 71: 522–525 4360948 10.1073/pnas.71.2.522 PMC388039

[embj2021109800-bib-0095] Strahl H , Burmann F , Hamoen LW (2014) The actin homologue MreB organizes the bacterial cell membrane. Nat Commun 5: 3442 24603761 10.1038/ncomms4442 PMC3955808

[embj2021109800-bib-0096] Strahl H , Errington J (2017) Bacterial membranes: structure, domains, and function. Annu Rev Microbiol 71: 519–538 28697671 10.1146/annurev-micro-102215-095630

[embj2021109800-bib-0097] Strahl H , Hamoen LW (2010) Membrane potential is important for bacterial cell division. Proc Natl Acad Sci USA 107: 12281–12286 20566861 10.1073/pnas.1005485107 PMC2901462

[embj2021109800-bib-0098] Suárez‐Germà C , Montero MT , Ignés‐Mullol J , Hernández‐Borrell J , Domènech Ò (2011) Acyl chain differences in phosphatidylethanolamine determine domain formation and LacY distribution in biomimetic model membranes. J Phys Chem B 115: 12778–12784 21962215 10.1021/jp206369k

[embj2021109800-bib-0099] Suutari M , Laakso S (1992) Unsaturated and branched chain‐fatty acids in temperature adaptation of *Bacillus subtilis* and *Bacillus megaterium* . Biochim Biophys Acta 1126: 119–124 1627613 10.1016/0005-2760(92)90281-y

[embj2021109800-bib-0100] Szekely P , Dvir T , Asor R , Resh R , Steiner A , Szekely O , Ginsburg A , Mosenkis J , Guralnick V , Dan Y *et al* (2011) Effect of temperature on the structure of charged membranes. J Phys Chem B 115: 14501–14506 21988313 10.1021/jp207566n

[embj2021109800-bib-0101] Thomason LC , Costantino N , Court DL (2007) *E. coli* genome manipulation by P1 transduction. Curr Protoc Mol Biol Chapter 1, Unit 1 17 10.1002/0471142727.mb0117s79 18265391

[embj2021109800-bib-0102] Torres MJ , Kew KA , Ryan TE , Pennington ER , Lin C‐T , Buddo KA , Fix AM , Smith CA , Gilliam LA , Karvinen S *et al* (2018) 17β‐Estradiol directly lowers mitochondrial membrane microviscosity and improves bioenergetic function in skeletal muscle. Cell Metab 27: 167–179 29103922 10.1016/j.cmet.2017.10.003 PMC5762397

[embj2021109800-bib-0103] Toulmay A , Prinz WA (2013) Direct imaging reveals stable, micrometer‐scale lipid domains that segregate proteins in live cells. J Cell Biol 202: 35–44 23836928 10.1083/jcb.201301039 PMC3704982

[embj2021109800-bib-0104] Typas A , Banzhaf M , Gross CA , Vollmer W (2012) From the regulation of peptidoglycan synthesis to bacterial growth and morphology. Nat Rev Microbiol 10: 123–136 10.1038/nrmicro2677 PMC5433867 22203377

[embj2021109800-bib-0105] Valentine DL (2007) Adaptations to energy stress dictate the ecology and evolution of the Archaea. Nat Rev Microbiol 5: 316–323 17334387 10.1038/nrmicro1619

[embj2021109800-bib-0106] Veatch SL (2007) From small fluctuations to large‐scale phase separation: Lateral organization in model membranes containing cholesterol. Semin Cell Dev Biol 18: 573–582 17942350 10.1016/j.semcdb.2007.08.016

[embj2021109800-bib-0107] van de Vossenberg JLCM , Driessen AJM , da Costa MS , Konings WN (1999) Homeostasis of the membrane proton permeability in *Bacillus subtilis* grown at different temperatures. Biochim Biophys Acta 1419: 97–104 10366675 10.1016/s0005-2736(99)00063-2

[embj2021109800-bib-0108] Wenzel M , Vischer NOE , Strahl H , Hamoen LW (2018) Assessing membrane fluidity and visualizing fluid membrane domains in bacteria using fluorescent membrane dyes. Bio‐protocol 8: e3063 34532528 10.21769/BioProtoc.3063 PMC8342135

[embj2021109800-bib-0109] Willecke K , Pardee AB (1971) Fatty acid‐requiring mutant of *Bacillus subtilis* defective in branched chain α‐keto acid dehydrogenase. J Biol Chem 246: 5264–5272 4999353

[embj2021109800-bib-0110] te Winkel JD , Gray DA , Seistrup KH , Hamoen LW , Strahl H (2016) Analysis of antimicrobial‐triggered membrane depolarisation using voltage sensitive dyes. Front Cell Dev Biol 4: 29 27148531 10.3389/fcell.2016.00029 PMC4829611

[embj2021109800-bib-0111] Zhu K , Zhang YM , Rock CO (2009) Transcriptional regulation of membrane lipid homeostasis in *Escherichia coli* . J Biol Chem 284: 34880–34888 19854834 10.1074/jbc.M109.068239 PMC2787350

