# Supplementary material for: Low membrane fluidity triggers lipid phase separation and protein segregation in living bacteria
Source: EMBO J. 2022 Jan 17;41(5):e109800. doi: 10.15252/embj.2021109800 (PMC8886542; doi:10.15252/embj.2021109800)
Supplement: Supplementary file 1 — Appendix [file EMBJ-41-e109800-s005.pdf]

Appendix

Low membrane fluidity triggers lipid phase separation and protein segregation in living bacteria

Marvin Gohrbandt[1], André Lipski[2], James W. Grimshaw[3], Jessica A. Buttress[3], Zunera Baig[3], Brigitte Herkenhoff[1], Stefan Walter[1], Rainer Kurre[4], Gabriele Deckers-Hebestreit[1,*] and Henrik Strahl[3,**]

1 Mikrobiologie, Fachbereich Biologie/Chemie, Universität Osnabrück, D-49069 Osnabrück, Germany
2 Lebensmittelmikrobiologie und -hygiene, Institut für Ernährungs- und Lebensmittelwissenschaften, Rheinische Friedrich-Wilhelms-Universität Bonn, D-53115 Bonn, Germany
3 Centre for Bacterial Cell Biology, Biosciences Institute, Faculty of Medical Sciences, Newcastle University, Newcastle upon Tyne, NE2 4AX, United Kingdom
4 Center of Cellular Nanoanalytics, Integrated Bioimaging Facility, Universität Osnabrück, D-49069 Osnabrück, Germany

*Corresponding author. Phone: +49 5419692809; E-mail: deckers-hebestreit@biologie.uni-osnabrueck.de

**Corresponding author. Phone: +44 1912083240; E-mail: h.strahl@ncl.ac.uk

Table of Contents

Figure S1 …… 2
Figure S2 …… 4
Figure S3 …… 6
Figure S4 …… 8
Figure S5 …… 10
Figure S6 …… 11
Figure S7 …… 13
Figure S8 …… 14
Figure S9 …… 16
Figure S10 …… 17
Figure S11 …… 18
Figure S12 …… 19
Figure S13 …… 20
Figure S14 …… 21
Figure S15 …… 23
Figure S16 …… 24
Table S1 …… 26
Table S2 …… 28
Table S3 …… 29
Table S4 …… 30
Table S5 …… 31
Table S6 …… 32
Appendix References …… 33

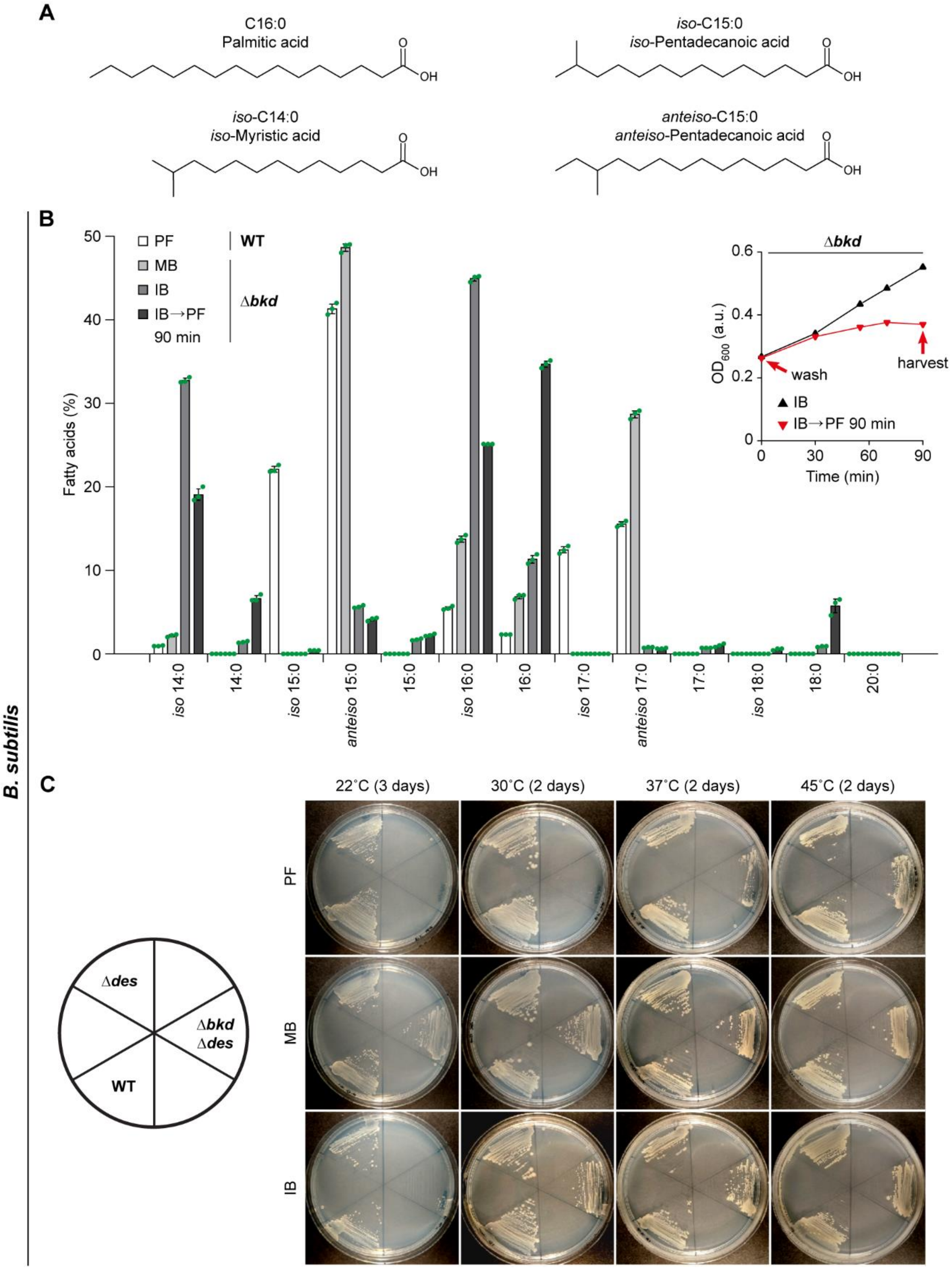

Figure S1. Depletion of branched chain fatty acids in *B. subtilis*.

A Structures of fatty acids typical for lipids in the plasma membrane of *B. subtilis* comprising BCFAs as fluidity-promoting and SFAs as fluidity-reducing fatty acids. Whereas IB is the precursor for

iso-fatty acids with even numbers of C atoms such as *iso*-C14:0 or *iso*-C16:0, MB is used for the synthesis of *anteiso*-C15:0 and *anteiso*-C17:0.

B Detailed fatty acid composition of *B. subtilis* wild type cells grown in the absence of fatty acid precursors, and cells of the fatty acid precursor auxotroph strain Δ*bkd* grown in the presence of MB, IB or grown precursor-free (PF) for 90 min (IB→PF). The determination was carried out through GC-MS of fatty acid methyl esters. The insert depicts the corresponding growth behaviour of the Δ*bkd* strain upon pre-culturing in the presence of IB, followed by wash and resuspension in either IB-supplemented or precursor-free (IB→PF 90 min) medium. The time point of cell harvest for the lipid analyses is indicated.

C Temperature-dependent growth behaviour of *B. subtilis* wild type cells, a strain deficient for the lipid desaturase Des (Δ*des*) and the fatty acid precursor auxotroph *B. subtilis* strain carrying deletions of both *des* and the *bkd* operon (Δ*bkd* Δ*des;* named "Δ*bkd*" for simplicity throughout the text) on solid growth medium supplemented either with MB, IB or grown precursor-free. Plates were incubated at temperatures of 45°C, 37°C and 30°C for 2 days, and 22°C for 3 days.

Data information: (B) The histogram depicts means and SD of biological triplicates for each strain and temperature condition. (C) Experiments are representative of three independent repeats. (B) *B. subtilis* 168, HS527; (C) *B. subtilis* 168, KS20, HS527.

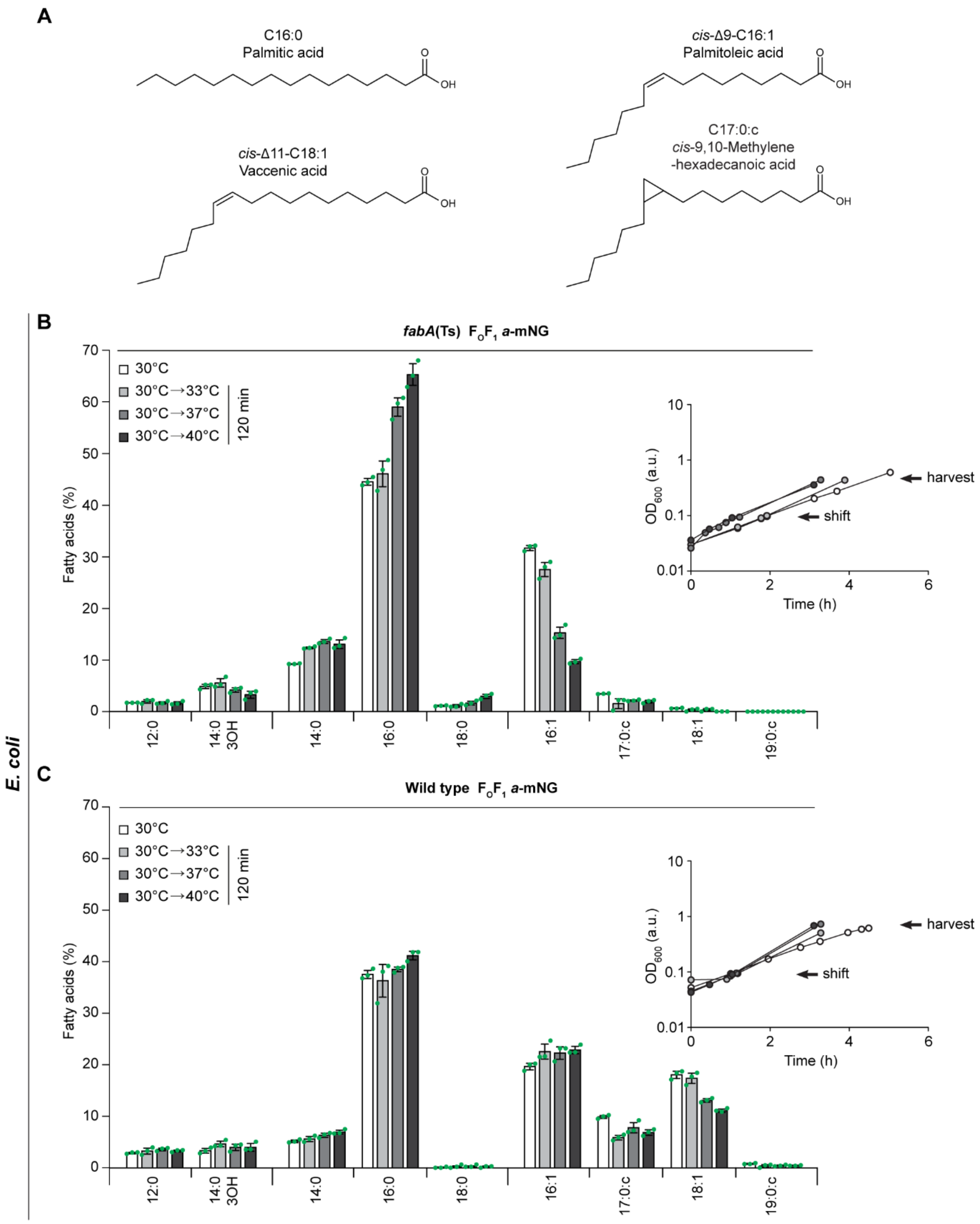

Figure S2. Depletion of unsaturated fatty acids in *E. coli.*

A Structures of fatty acids typical for lipids in the plasma membrane of *E. coli* comprising UFAs as fluidity-promoting and SFAs as fluidity-reducing fatty acids. Phospholipids contain as main fatty acids C16:0 and C16:1, while the amount of C18:1 is decreased at higher temperatures. During oxidative or acid stress as well as in the stationary growth phase, UFAs are converted into cyclopropane fatty acids (CFA) such as C17:0:c or C19:0:c to retain the *cis* double-bond configuration.

B Detailed fatty acid composition of the temperature-sensitive *E. coli* strain *fabA*(Ts) (*fabF fabA*(Ts); named "*fabA*(Ts)" for simplicity throughout the text) grown at 30°C, or grown at 30°C followed by a shift to 33°C, 37°C or 40°C for 120 min.

C Detailed fatty acid composition of *E. coli* wild type cells grown as described in panel B for *fabA*(Ts).

B, C Inserts show the corresponding growth curves. The time point of the temperature shift as well as the cell harvest for the lipid analyses is indicated. Fatty acids C12:0 and C14:0-3OH were not included into the summarised fatty acid profiles shown in Fig 1E due to their sole presence in lipopolysaccharide molecules. The determination was carried out through GC-MS of fatty acid methyl esters.

Data information: (B, C) The histograms depict means and SD of biological triplicates for each strain and temperature condition. Strains used: (B) *E. coli* MG4; (C) *E. coli* MG1, (strains Y-Mel and UC1098, respectively, additionally encoding fluorescent ATP synthase (F_OF_1 *a*-mNG)).

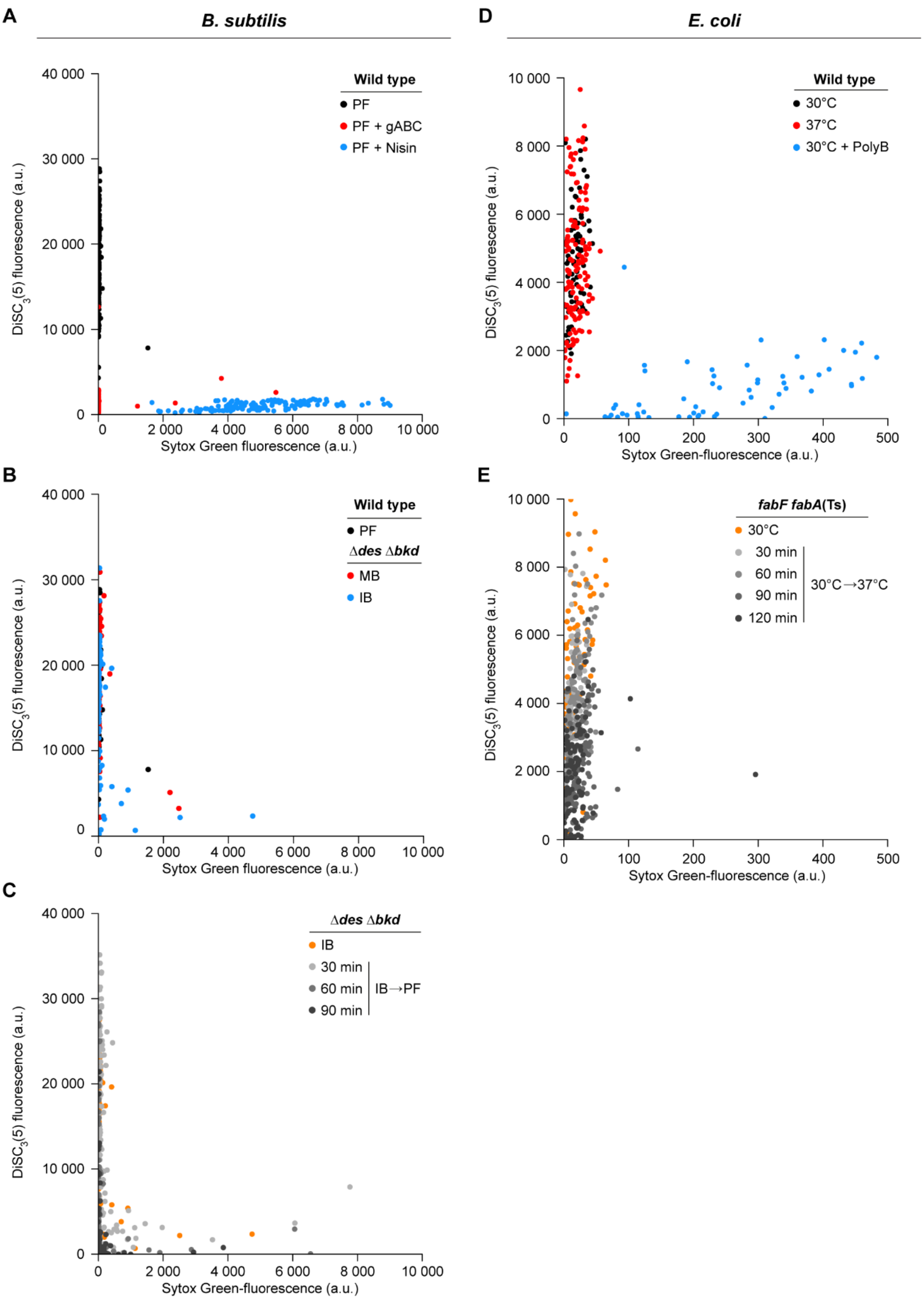

Figure S3. Cross-correlation between membrane depolarisation and membrane permeabilisation.

A Correlation between membrane depolarisation and permeabilisation in *B. subtilis* wild type cells co-stained with the membrane potential-sensitive dye DISC3(5) and the membrane permeability indicator Sytox Green, respectively. Depicted are the quantified cellular fluorescence signals for

untreated wild type cells grown in the absence of fatty acid precursors, and the same cells incubated with gramicidin ABC (gABC), a membrane-depolarising but not permeabilising antimicrobial peptide, or with permeabilising, pore-forming lantibiotic nisin.

B Correlation between membrane depolarisation and permeabilisation in *B. subtilis* fatty acid precursor auxotroph strain Δ*bkd* grown in the presence of fatty acid precursor MB or IB and co-labelled with $DiSC_3(5)$ and Sytox Green.

C Correlation between membrane depolarisation and permeabilisation in *B. subtilis* Δ*bkd* grown in IB, washed by centrifugation, further incubated in the absence of fatty acid precursors for the time points indicated and co-labelled with $DiSC_3(5)$ and Sytox Green.

D Correlation between membrane depolarisation and permeabilisation in *E. coli* wild type cells grown at 30°C or 37°C and co-labelled with $DiSC_3(5)$ and Sytox Green. As a control, wild type cells grown at 30°C were incubated in the presence of the pore-forming antibiotic Polymyxin B.

E Correlation between membrane depolarisation and permeabilisation in *E. coli fabA*(Ts) cells grown at 30°C and transferred to the non-permissive temperature of 37°C for the time points indicated, and co-stained with $DiSC_3(5)$ and Sytox Green.

Data information: (A-E) Data shown here represent the same dataset (n= 100-142) used for generating the graphs shown in Fig 3B and D, but were additionally analysed for the Sytox Green fluorescent signal and its correlation with corresponding $DiSC_3(5)$ fluorescence signals. Strains used: (A-C) *B. subtilis* 168, HS527; (D, E) *E. coli* Y-Mel, UC1098.

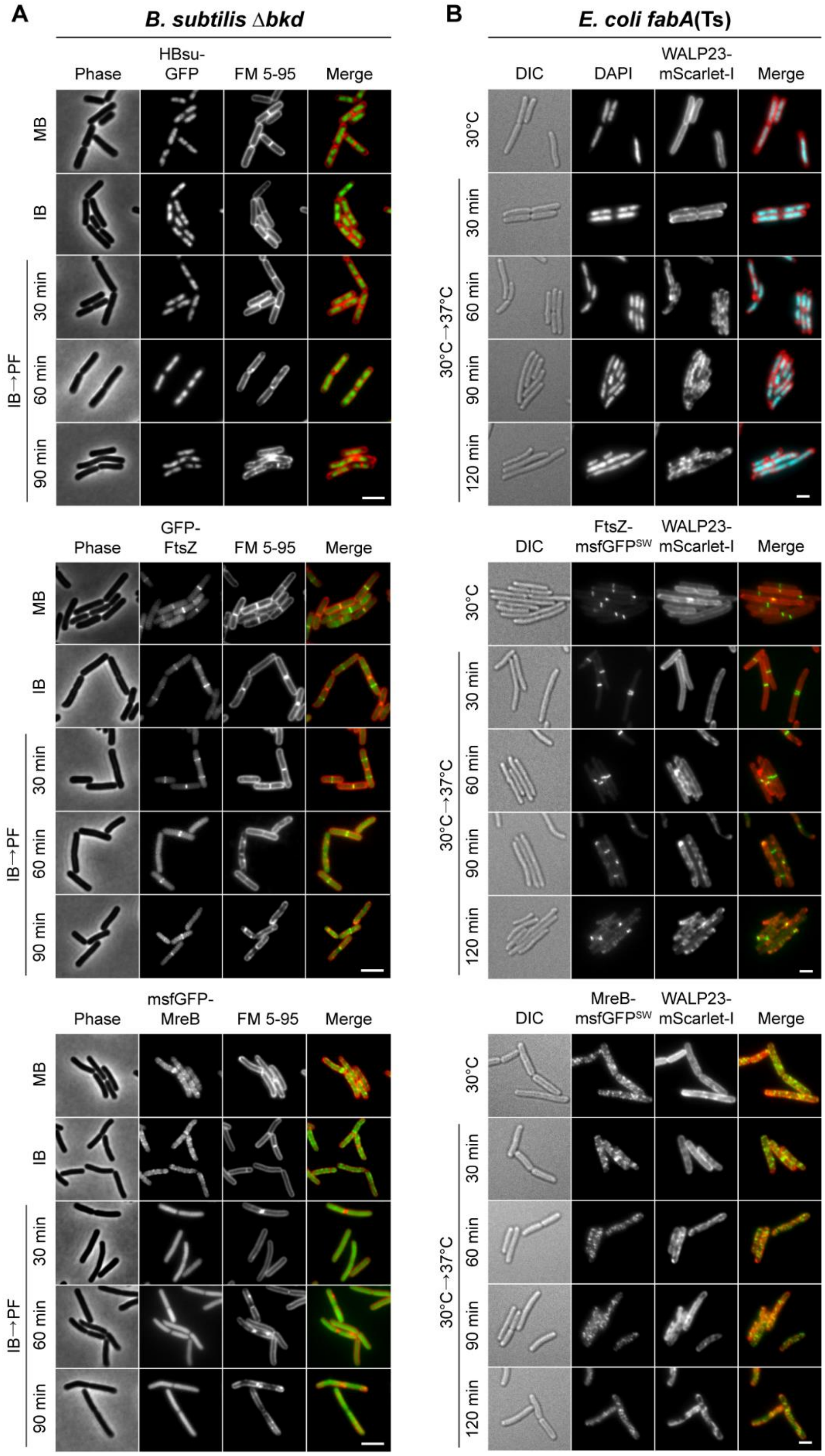

Figure S4. Consequences of low membrane fluidity on cell morphogenesis.

A Detailed phase contrast and fluorescence images of *B. subtilis* fatty acid precursor auxotroph strain Δ*bkd* stained with the membrane dye FM 5-95 and expressing GFP fusions to the sequence-unspecific DNA-binding protein HBsu (top), cell division protein FtsZ (middle) or cell

elongation protein MreB (bottom). Depicted are cells grown in the presence of the precursor IB or MB, and cells grown with IB, washed followed by precursor-free incubation (IB→PF) for the time points indicated.

B Detailed differential interference contrast (DIC) and fluorescence images of *E. coli fabA*(Ts) cells, incubated with dye FM 5-95 (staining the outer membrane), were labelled in addition with DAPI for DNA (top), or expressed as chromosomally encoded GFP fusions to FtsZ (middle) and MreB (bottom), respectively. Depicted are cells grown at the permissive growth temperature (30°C) and upon growth shift to the non-permissive temperature (37°C) for the time points indicated.

A, B The panels provide additional examples and time points for the images shown in Fig 4.

Data information: Experiments are representative of biological triplicates. (A) Scale bar 3 µm. (B) Scale bar 2 µm. Strains used: (A) *B. subtilis* HS541, HS548, HS549; (B) *E. coli* UC1098, BHH100, BHH101.

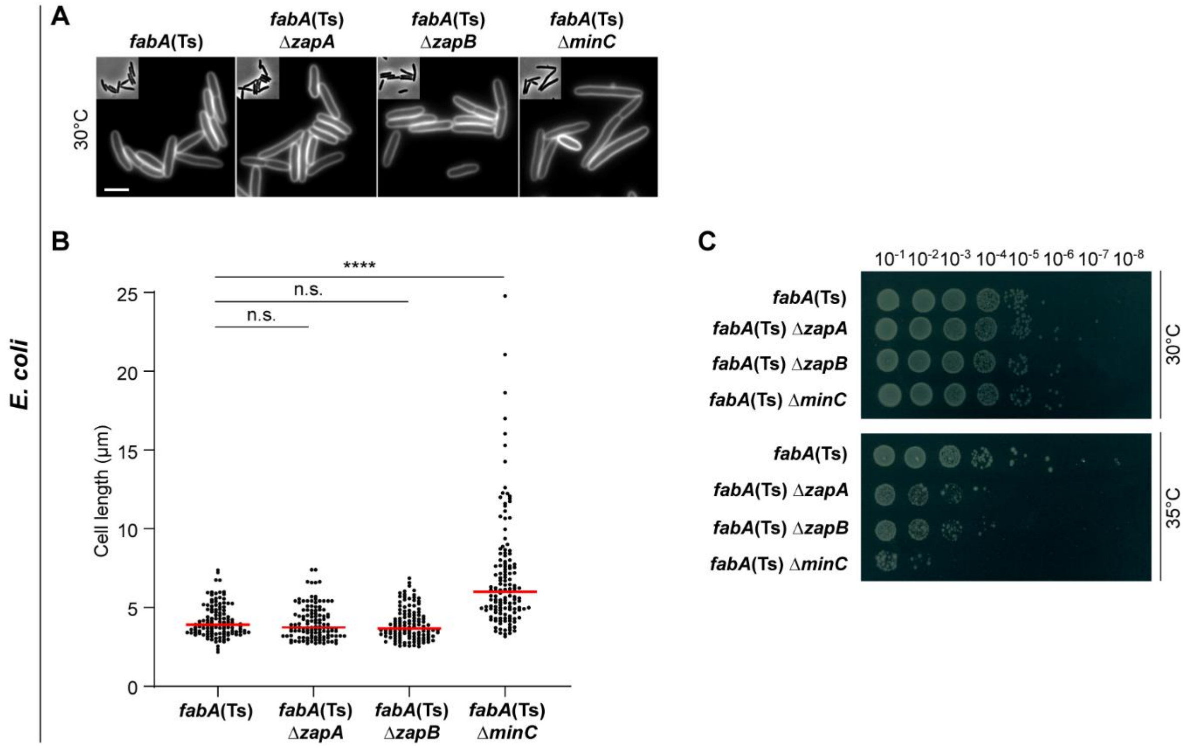

Figure S5. Destabilisation of the *E. coli* divisome by deletion of the non-essential division genes *zapA or zapB* triggers increased sensitivity towards low membrane fluidity.

A Images of *E. coli* cells carrying *fabA*(Ts), and *fabA*(Ts) combined with Δ*zapA,* Δ*zapB* and Δ*minC*, grown at the permissive temperature (30°C). Cells were stained with the outer membrane dye FM 5-95 prior to microscopy. Note the clear cell elongation of the *fabA*(Ts) Δ*minC* strain whilst Δ*zapA* and Δ*zapB* does not significantly increase the cell length of already slightly elongated *fabA(Ts)* cells.

B Quantification of cell length for cells (n=119-136) depicted in panel A. Red lines indicate the median cell length.

C Viability of the strains depicted in panel A upon incubation on agar plates in M9-glucose minimal medium overnight at different temperatures. The serial dilutions and spot assays were carried out with pre-cultures grown at 30°C to mid-log growth phase. Note the temperature hypersensitivity of strain *fabA*(Ts) Δ*minC*, and the increased sensitivity of the *fabA*(Ts) Δ*zapA* and *fabA*(Ts) Δ*zapB* strains at 35°C.

Data information: (A-C) The experiments are representative of biological triplicates. (B) Red lines indicate the median, while the P values represent results of unpaired, two-sided t-tests. Significance was assumed with **** $p < 0.0001$, *** $p < 0.001$, ** $p < 0.01$, * $p < 0.05$, n.s., not significant. (A) Scale bar: 3 µm. Strains used: (A-C) *E. coli* UC1098, UC1098.Δ*zapA,* UC1098.Δ*zapB,* UC1098.Δ*minC.*

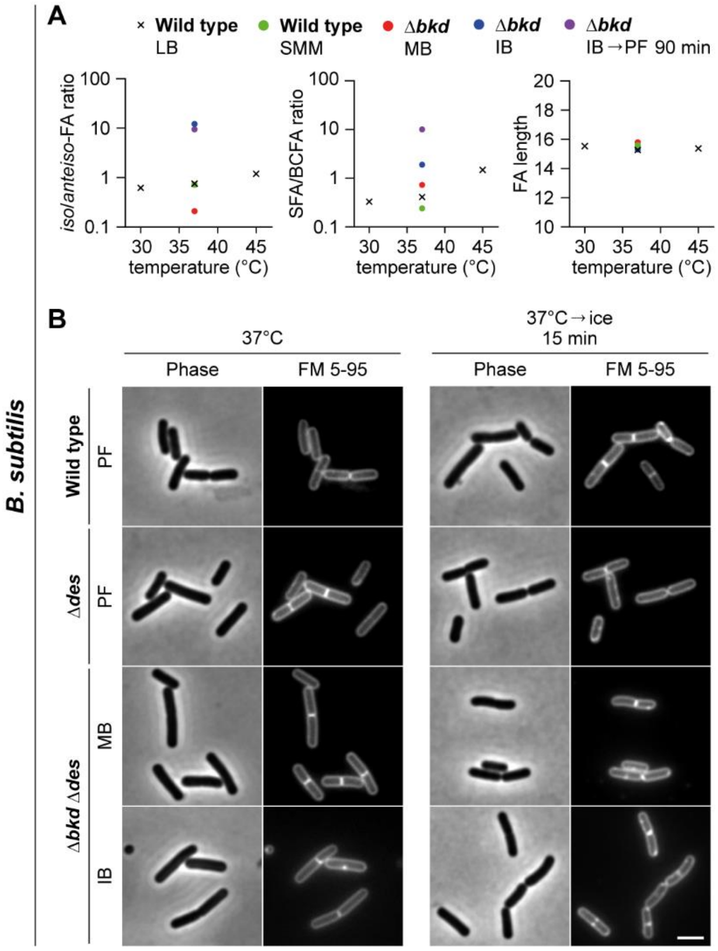

Figure S6. Changes in *B. subtilis* fatty acid profile required to induce lipid phase separation are rather extreme.

A Comparison of changes in fatty acid (FA) composition observed for *B. subtilis* fatty acid precursor-auxotrophic Δ*bkd* strain upon changes in precursor availability, and *B. subtilis* wild type strain upon normal homeoviscous adaptation to different growth temperatures. Depicted are changes in the *iso/anteiso*-FA ratio (left panel), which is the main mechanism of membrane temperature adaptation in *B. subtilis*, changes in the ratio between SFA and BCFA (middle panel), and changes in the fatty acid chain length (right panel). The profiles were obtained for WT cells grown in LB medium or Spizizen minimal medium (SMM) at 30°C, 37°C and 45°C, respectively, and for the Δ*bkd* strain upon supplementation with 2-methyl butyric acid (MB), isobutyric acid (IB) or grown precursor-free (PF) for 90 min. Note that this is the same dataset as shown in Fig 1 and Appendix Fig S1 for Δ*bkd.* While the temperature-dependent adaptation of *B. subtilis* fatty acid profile is observed both in terms of *iso/anteiso*-FA and SFA/BCFA ratio, the changes are minor compared to the more substantial changes induced by fatty acid precursor availability. Also note that, despite the large changes in the fatty acid types, the homeostasis of the fatty acid chain length remains unaffected.

B Consistent with the large shifts in both *iso/anteiso*-FA and SFA/BCFA, required to induce large scale *in vivo* lipid phase separation, cold shock carried out by incubation on ice for 15 min is only capable for inducing minor membrane irregularities (compare with Fig 4A). The images depict

phase contrast and fluorescent FM 5-95-stained wild type and Δ*des* cells, together with Δ*bkd* Δ*des* cells supplemented with MB or IB. The cells were grown at 37°C (left panels) followed by incubation on ice for 15 min (right panels).

Data information: (A, B) The experiments are representative of biological duplicates. (B) Scale bar: 3 µm. Strains used: (A) *B. subtilis* 168, HS526; (B) *B. subtilis* 168, KS20, HS527.

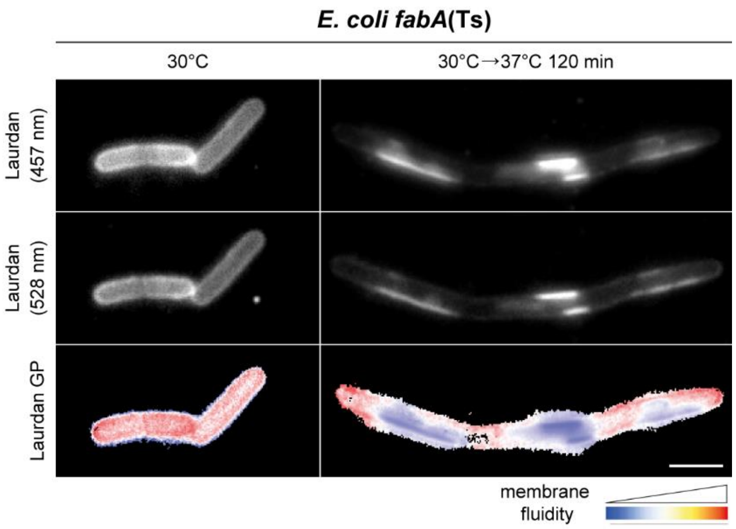

Figure S7. Consequences of low membrane fluidity on membrane homogeneity.

Fluorescence images of *E. coli fabA*(Ts) cells grown in LB medium at permissive 30°C or shifted to the non-permissive temperature of 37°C for 120 min. Cells were stained with membrane fluidity-sensitive dye Laurdan and imaged at 457 nm, 528 nm and as the corresponding colour-coded Laurdan GP map.

Data information: The experiment is representative of biological triplicates. Scale bar, 3 µm. Strain used: *E. coli* UC1098.

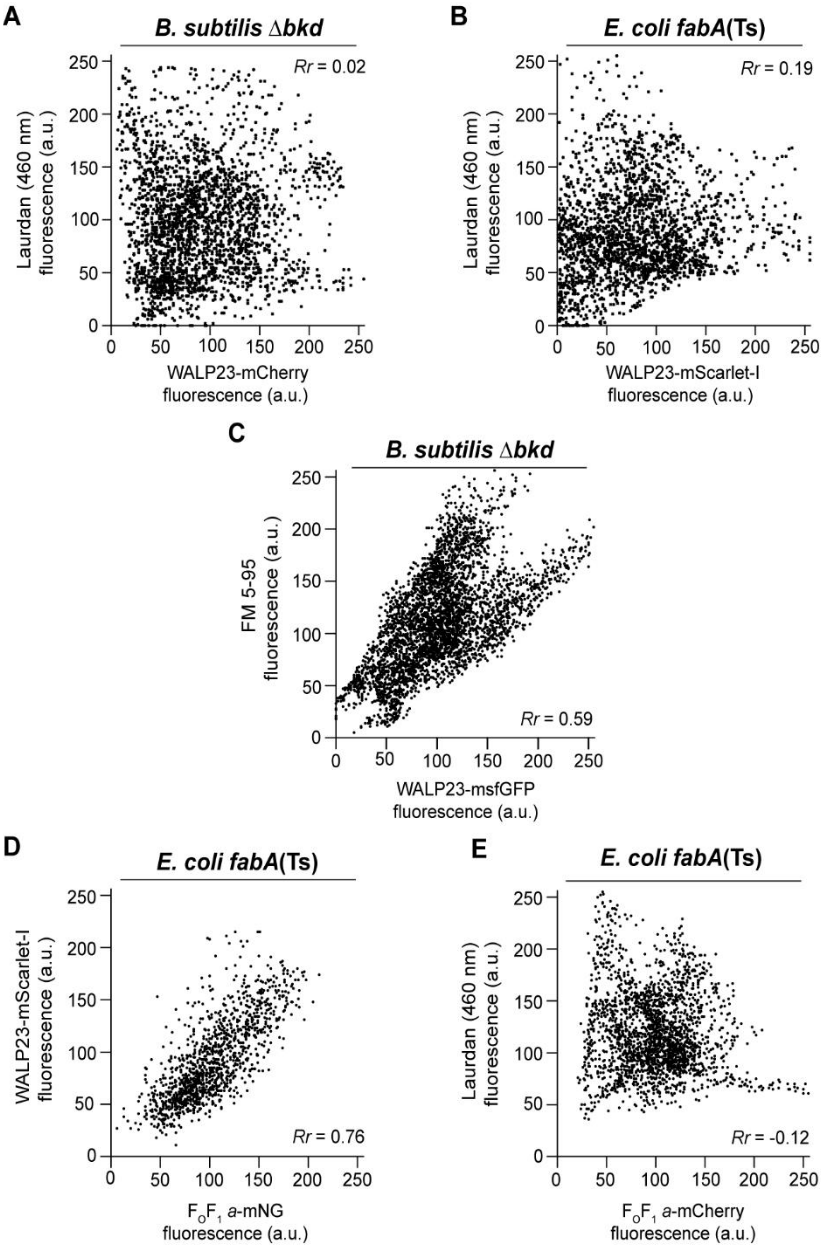

Figure S8. Co-localisation correlation of the fluorescent dyes and marker proteins used in phase-separated membranes of *B. subtilis* and *E. coli.*

A Pixel-by-pixel intensity correlation between WALP23-mCherry and Laurdan (460 nm) fluorescent signals in *B. subtilis* Δ*bkd* cells grown for 90 min in the absence of fatty acid precursor (IB→PF) as shown in Fig 5D. The low Pearson's correlation coefficient (*Rr*) indicates lack of co-localisation.

B Pixel-by-pixel intensity correlation between WALP23-mScarlet-I and Laurdan (460 nm) fluorescent signals in *E. coli fabA*(Ts) cells grown at 30°C and shifted for 120 min to the non-permissive temperature of 37°C as shown in Fig 5E. The low Pearson's correlation coefficient (*Rr*) again indicates lack of co-localisation.

C Pixel-by-pixel intensity correlation between WALP23-msfGFP and FM 5-95 fluorescent signals in *B. subtilis* Δ*bkd* cells grown for 90 min in the absence of fatty acid precursor (IB→PF) as shown in Appendix Fig S9. The high *Rr* value indicates significant co-localisation.

D Pixel-by-pixel intensity correlation between WALP23-mScarlet-I and F_oF_1 *a*-mNG in *E. coli fabA*(Ts) cells grown at 30°C and shifted for 120 min to the non-permissive temperature of 40°C as shown in Fig 6B. The high *Rr* value indicates significant co-localisation.

E Pixel-by-pixel intensity correlation between F_OF_1 *a*-mCherry and Laurdan (460 nm) fluorescent signals in *E. coli fabA*(Ts) cells grown at 30°C and shifted for 120 min to the non-permissive temperature of 37°C as shown in Fig 6C. The low *Rr* value indicates lack of co-localisation.

Data information: (A-E) Experiments are representative for three independent repeats. Strains used: (A) *B. subtilis* HS547; (B) *E. coli* UC1098/pBH501; (C) *B. subtilis* HS552; (D) *E. coli* MG4/pBH501; (E) *E. coli* LF6.red.

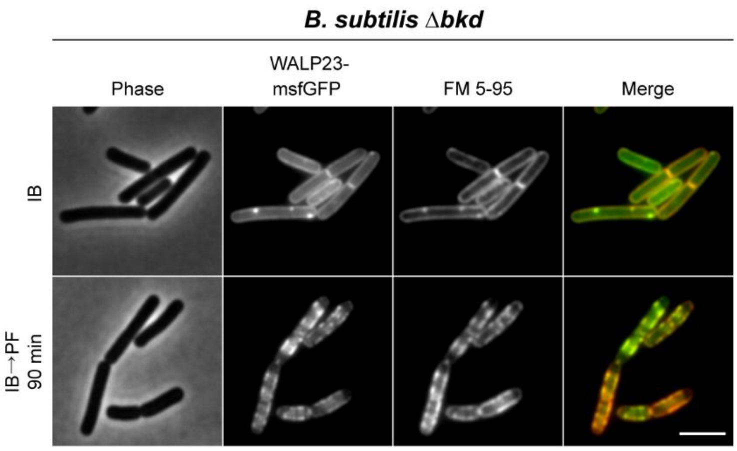

Figure S9. Co-localisation of membrane dye FM 5-95 and transmembrane marker peptide WALP23 in phase-separated membranes of *B. subtilis.*

Phase contrast and fluorescence images of fatty acid precursor auxotroph *B. subtilis* strain Δ*bkd* grown in the presence of IB and in the absence of precursor for 90 min (IB→PF). Depicted are cells expressing WALP23-msfGFP, which were labelled with fluorescent dye FM 5-95. For fluorescence intensity correlations, see Appendix Fig S8C.

Data information: The experiment is representative for three independent repeats. Strain used: *B. subtilis* HS552.

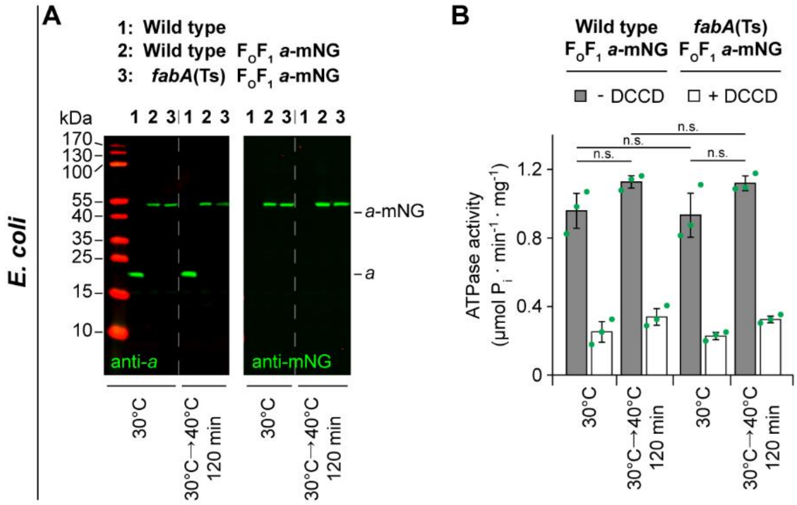

Figure S10. Stability and activity of mNG-labelled F_OF_1 ATP synthase in *E. coli.*

A Stability of F_OF_1 *a*-mNG tested by Western blotting with antibodies specific for membrane-integral subunit F_O-*a* (GDH 14-5C6) or mNG (32F6) using inverted membrane vesicles prepared from *E. coli* wild type and *fabA*(Ts) cells, respectively, both chromosomally expressing F_OF_1 *a*-mNG. Cells were grown either at 30°C or grown at 30°C followed by transfer to 40°C for 120 min.

B Functionality of F_OF_1 *a*-mNG verified by ATPase activities measured in inverted membrane vesicles in the absence or presence of F_OF_1 inhibitor *N, N*'-dicyclohexylcarbodiimide (DCCD).

Data information: (A) The experiment is representative for technical triplicates. (B) The diagram depicts mean and SD of technical triplicates for each strain and condition. P values represent the results of unpaired, two-sided t-tests. Insignificant changes (p>0.1) are indicated with n.s., not significant. (A) *E. coli* Y-Mel, MG1, MG4; (B) *E. coli* MG1, MG4.

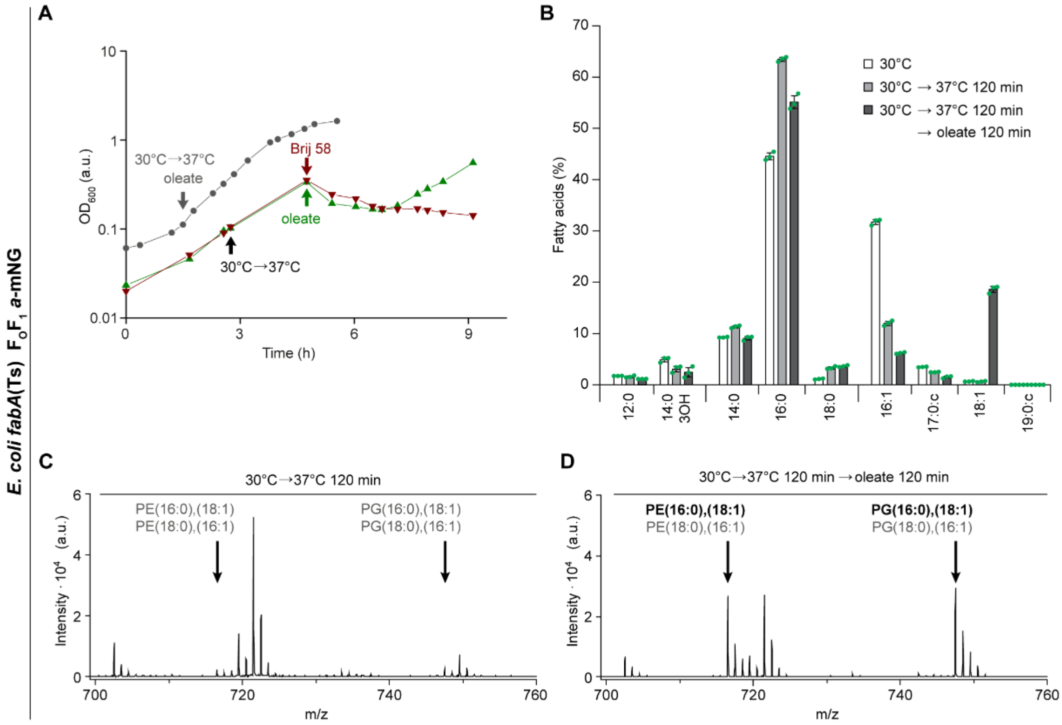

Figure S11. Recovery of phase-separated plasma membranes of *E. coli fabA*(Ts) by lipid-incorporation of exogenous UFA oleate (*cis*-Δ9-C18:1).

A Growth curves depicting the growth inhibition of *E. coli fabA*(Ts) encoding F_OF_1 *a*-mNG upon transfer to the non-permissive temperature of 37°C, which can be rescued by addition of UFA oleate dissolved in Brij 58 (green triangles), but not by Brij 58 alone (dark red triangles). Supplementing the medium with oleate immediately upon shift to the non-permissive temperature prevents growth arrest (gray circles).

B Detailed fatty acid profiles of F_OF_1 *a*-mNG-expressing *E. coli fabA*(Ts) upon growth at the permissive temperature of 30°C, upon depletion of UFA by a shift to non-permissive 37°C for 120 min and upon recovery by subsequent supplementation with oleate for additional 120 min at 37°C.

C MALDI-TOF MS (matrix-assisted laser desorption ionization – time of flight mass spectrometry) spectra of lipids extracted from *fabA*(Ts) cells grown at 30°C and shifted to non-permissive 37°C for 120 min.

D MALDI-TOF MS spectra of lipids extracted from *fabA*(Ts) cells grown at 30°C, shifted to 37°C for 120 min and subsequently supplemented with oleate for 120 min at 37°C. The intensities of *m/z*-values 716.5 and 747.5, only present in minute amounts after the 120 min-shift to 37°C (panel C), showed a strong increase after growth in the presence of oleate for 120 min. Both masses were identified by standard lipids as well as fragmentation of the mother ions (MALDI-TOF/TOF analyses) to be mainly PE(16:0),(18:1) and PG(16:0),(18:1), respectively, confirming the incorporation of oleate into phospholipids of *E. coli fabA*(Ts).

Data information: (A-D) Results shown are representative for biological triplicates. (B) The histogram depicts mean and SD of biological triplicates. Strain used: (A-D) *E. coli* MG4.

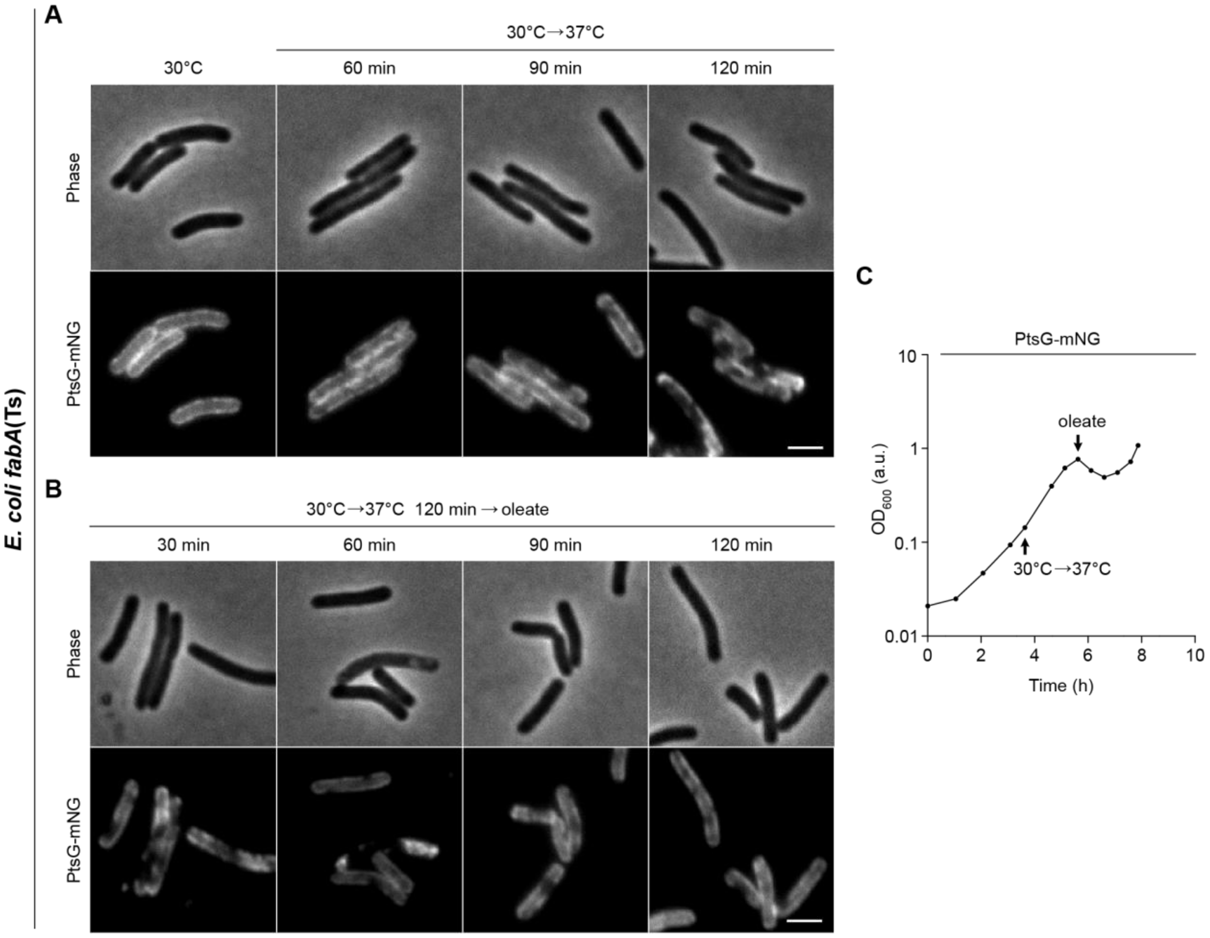

Figure S12. Segregation of glucose permease (PtsG) into the fluid phase of phase-separated plasma membranes of *E. coli fabA*(Ts) and its re-localisation in the presence of UFA oleate.

A Phase and fluorescence images of *E. coli fabA*(Ts) cells chromosomally expressing mNG-labelled glucose permease PtsG. Depicted are cells grown at 30°C, and those shifted to the non-permissive temperature 37°C for the time points indicated, leading to PtsG-mNG partitioning within the membrane.

B Phase and fluorescence images of the same strain as depicted in panel A, but imaged after supplementation of the growth medium with oleate at 37°C for further 120 min, leading to a reversion of the protein segregation observed in panel A.

C Corresponding growth curve of *E. coli* strain *fabA*(Ts) chromosomally expressing *ptsG-mNG* in M9-glucose minimal medium with the temperature shift (30°C→37°C) and the supplementation with oleate as indicated.

Data information: (A-C) Results shown are representative for biological triplicates. (A, B) Scale bar: 2 µm. Strain used: (A-C) *E. coli* UC1098.PtsG-mNG.

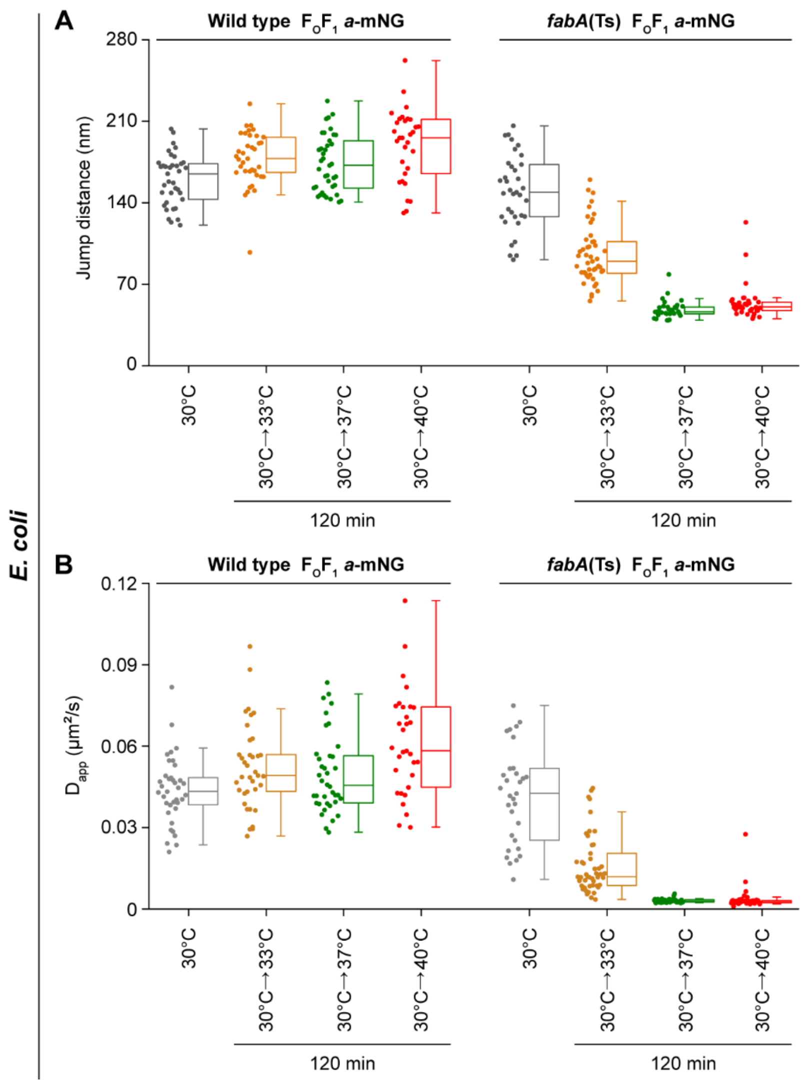

Figure S13. Reduced lateral mobility of F_OF_1 *a*-mNG after UFA depletion in *E. coli fabA*(Ts).

A Analysis of the cell-to-cell heterogeneity of F_OF_1 *a*-mNG median jump distances. The box plot depicts the distribution of median jump distances of all trajectories determined for individual cells of *E. coli* wild type and *fabA*(Ts) at the growth temperatures indicated.

B Analysis of the cell-to-cell heterogeneity of F_OF_1 *a*-mNG apparent diffusion coefficients (D_{app}) The box plot depicts the D_{app} calculated for all trajectories determined for individual cells of *E. coli* wild type and *fabA*(Ts) at the growth temperatures indicated.

Data information: (A) The analysis was carried out with cells (n=31-47) from 3-5 biological replicates for each condition and strain, using 76±28 trajectories with ≥5 consecutive frames per cell. The interquartile range defines 50% of the data including the corresponding median, while the whiskers represent the borders of 25% and 75%, respectively, of the cells. (B) The D_{app} of all trajectories within individual cells was calculated using the same data set as in panel A. The interquartile range and the whiskers were defined as in panel A. (A-C) For the analyses presented the same dataset as in Fig 7 was used. For detailed information on cell numbers and jump distances, see Appendix Table S2. Strains used: (A, B) *E. coli* MG1, MG4.

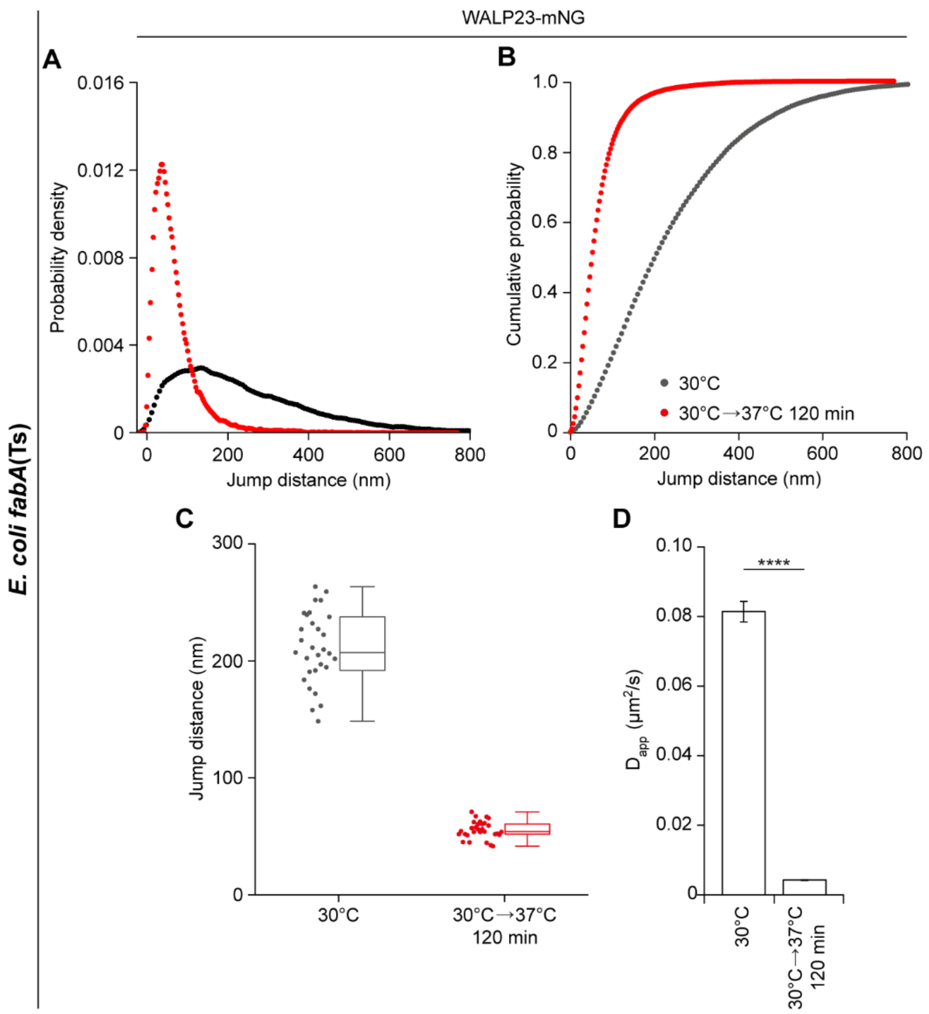

Figure S14. Reduced lateral mobility of transmembrane peptide WALP23 upon UFA depletion in *E. coli fabA*(Ts).

A Probability density plots of WALP23-mNG jump distances in *fabA*(Ts) cells grown at permissive 30°C or shifted to the non-permissive temperature of 37°C for 120 min.

B Cumulative probability plot of jump distances of WALP23-mNG in *fabA*(Ts) cells grown at permissive 30°C or shifted to the non-permissive temperature of 37°C for 120 min.

C Analysis of the cell-to-cell heterogeneity of WALP23-mNG median jump distances. The box plot depicts the distribution of median jump distances all trajectories determined for individual *E. coli* wild type and *fabA*(Ts) cells grown at the temperatures indicated.

D Corresponding apparent diffusion coefficients (D_{app}) of WALP23-mNG calculated from trajectories analysed in panel A.

Data information: (A; B) Trajectories with ≥5 consecutive frames, respectively, were pooled from biological triplicates and used for analysis (n=3305 (30°C); n=2801 (30°C→37°C)). (C) The analysis was carried out with cells (n=30) from biological triplicates for each condition. The interquartile range defines 50% of the data including the corresponding median, while the whiskers represent the borders of 25% and 75%, respectively, of the cells. See Appendix Table S3 for detailed information on cell

numbers and jump distances. (D) The histogram depicts mean and SD from biological triplicates, together with P values of a two-sided Wilcoxon rank sum test. Significance was assumed with **** $p < 0.0001$, *** $p < 0.001$, ** $p < 0.01$, * $p < 0.05$, n.s., not significant. Strain used: (A-D) *E. coli* UC1098/pBH500.

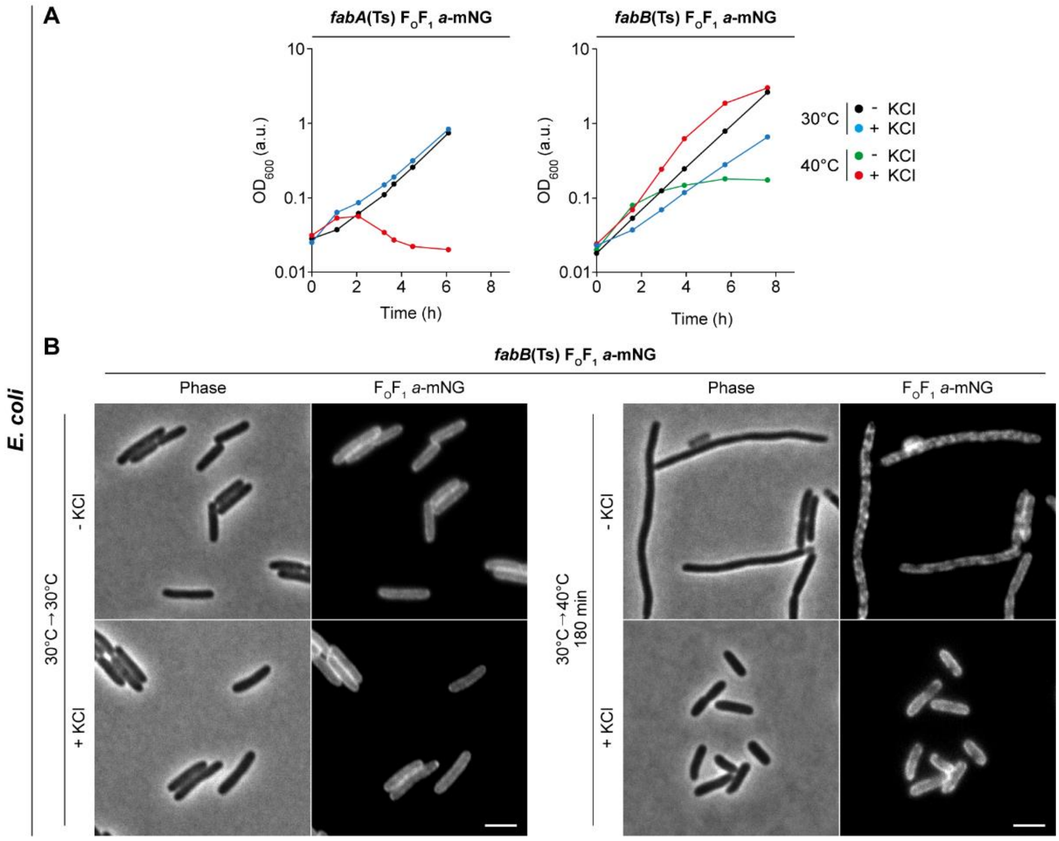

Figure S15. Osmotic stabilisation of *E. coli fabB15*(Ts) enables cell growth at non-permissive temperature and prevents partially the partitioning of F_OF_1 *a*-mNG.

A Comparison of temperature-dependent growth behaviour of *E. coli* strains *fabA*(Ts) (left panel) and *fabB15*(Ts) (*fabF fabB15*(Ts); named "*fabB15*(Ts)" for simplicity throughout the text) (right panel) using KCl for osmotic stabilisation. Cells were grown in M9-glucose minimal medium in the absence or presence of 2% (w/v) KCl using for inoculation cultures grown overnight correspondingly at 30°C. Both strains grew comparably at 30°C. At non-permissive 40°C, *fabB15*(Ts) was able to grow in the presence of 2% KCl, while *fabA*(Ts) could not and cells started to lyse after 120 min. The inability of the *fabA*(Ts) strain UC1098 to retain growth through osmotic stabilisation is a well-known observation. From 17 tested UFA auxotrophic *E. coli* strains only 11 could maintain cell growth by osmotic stabilisation (Akamatsu, 1974; Broekman & Steenbakkers, 1973). This demonstrates that growth at non-permissive temperature in a high-osmotic medium is not a common characteristic of mutants impaired in the biosynthesis of unsaturated fatty acids.

B Phase and fluorescence images of *E. coli fabB15*(Ts) cells chromosomally expressing F_OF_1 *a*-mNG. At 30°C cells exhibited a homogeneous distribution of F_OF_1 *a*-mNG independent of KCl supplementation. Cells shifted to 40°C for 180 min without KCl supplementation developed significant partitioning of F_OF_1 *a*-mNG, which was largely surpassed by KCl. Also note the clear increase in cell length of *fabB15*(Ts) observed without KCl supplementation at 40°C indicating severe cell division defects.

Data information: (A, B) Experiments are representative of three independent repeats. (B) Scale bar: 2 µm. Strains used: (A) *E. coli* MG1, BHH87; (B) *E. coli* BHH87.

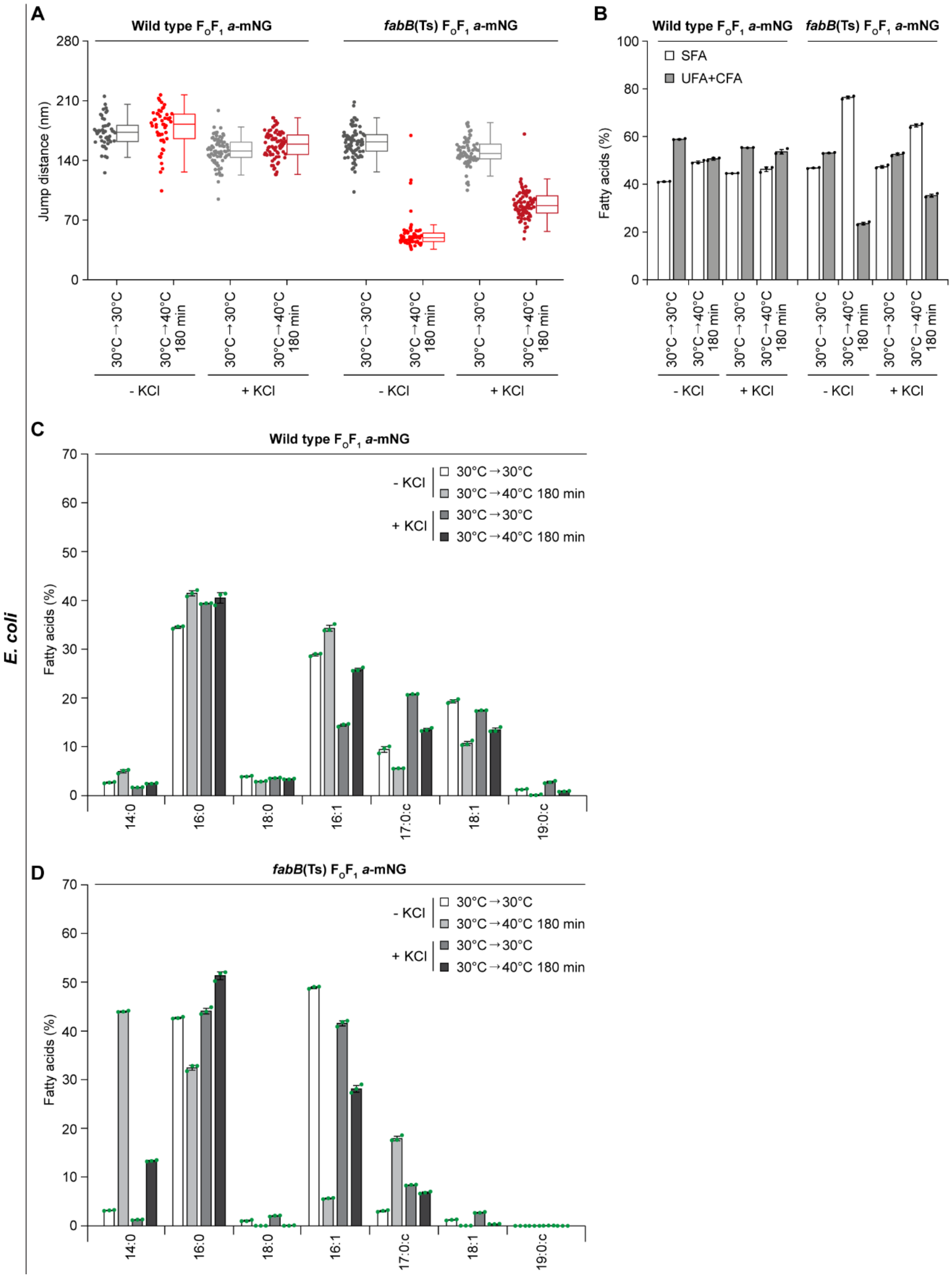

Figure S16. Osmotic stabilisation of lateral diffusion of F_OF_1 *a*-mNG in UFA-depleted membranes of *E. coli fabB15*(Ts).

A Lateral mobility of F_OF_1 *a*-mNG upon osmotic KCl stabilisation. Distribution of median jump distances of F_OF_1 *a*-mNG in different cells. The box plot depicts the distribution of all trajectories

determined for *E. coli* WT and *fabB15*(Ts) cells at growth temperatures and KCl supplementation as indicated. Supplementation of M9-glucose minimal medium with 2% (w/v) KCl resulted in a slight decrease in lateral mobility of F_OF_1 *a*-mNG in WT cells grown at 30°C or grown at 40°C for 180 min. The same was observed for *fabB15*(Ts) cells grown at permissive 30°C. Cells of *fabB15*(Ts) grown at non-permissive 40°C for 180 min in the presence of 2% KCl maintained intermediate lateral diffusion rates for F_OF_1 *a*-mNG (app. 60% compared to *fabB15*(Ts) cells grown at 30°C without KCl), whereas at 40°C for 180 min without KCl supplementation, a more substantial reduction of lateral displacement was observed, comparable to that monitored for *fabA*(Ts) (see Fig 7 and Appendix Fig S13).

B Fatty acid composition (SFA versus UFA/CFA ratio) of *E. coli* WT and *fabB15*(Ts) cells. The same cell cultures as for imaging in panel A were analysed. *E. coli* WT cells at both temperatures and *fabB15*(Ts) cells grown at 30°C were essentially unaffected in their fatty acid composition from osmotic changes in the medium. Cells of *fabB15*(Ts) grown at 40°C for 180 min showed a strong decrease in UFA in the absence of additional KCl, comparable to *fabA*(Ts) (see Fig 1E), while in the presence of KCl the amount of UFA+CFA remained at intermediate levels.

C, D Detailed fatty acid composition of *E. coli* WT cells (C) and *E. coli fabB15*(Ts) (D) grown as described in panel A. Note the high content of C14:0 in the absence of KCl at 40°C, which may be an adaptive response to fluidise the membrane upon low UFA conditions; an observation also described for a different *fabB* strain (Budin *et al*, 2018). In high-osmotic medium, the higher content of UFA present may be due to an osmotic stabilisation of the temperature-sensitive enzyme carrying the substitution A329V located within the interior of each monomer of the homodimeric enzyme (Olsen *et al*, 1999). Fatty acid compositions were calculated from lipid species analyses determined by MALDI-TOF/TOF MS.

Data information: (A) The analysis was carried out for n=47-88 cells from biological triplicates for each condition and strain, using 100$\pm$25 trajectories with $\geq$5 consecutive frames per cell. The interquartile range defines 50% of the data including the corresponding median, while the whiskers represent the borders of 25% and 75%, respectively. See Appendix Table S4 for detailed information on cell numbers and jump distances. (B-D) Graphs depict mean and SD of biological triplicates. Strains used: (A, B) *E. coli* MG1, BHH87; (C) *E. coli* MG1; (D) *E. coli* BHH87.

Table S1. Strains

Strain	Relevant Genotype	Induction	Source/Reference
B. subtilis 168	*trpC2*	-	(Barbe *et al*, 2009)
B. subtilis HB5134	*des::spc*	-	(Hachmann *et al*, 2009)
B. subtilis LC5	*des::kan*	-	(Altabe et al, 2003)
B. subtilis KS20	*des::spc*	-	This work
B. subtilis RM113	*spoVD::cat Pxyl-murE sepFT11M bkd::ery*	-	(Mercier *et al*, 2012)
B. subtilis HS526	*bkd::ery*	-	This work
B. subtilis HS527	*bkd::ery des::spc*	-	This work
B. subtilis HS550	*bkd::ery des::kan*	-	This work
B. subtilis JWV042	*amyE::cat Phbs-hbs-gfp*	-	(Strahl & Hamoen, 2010)
B. subtilis HS541	*amyE::cat Phbs-hbs-gfp bkd:ery des::spc*	-	This work
B. subtilis 2020	*amyE::spc Pxyl-gfp-ftsZ*	-	(Stokes *et al*, 2005)
B. subtilis HS548	*amyE::spc Pxyl-gfp-ftsZ bkd::ery des::kan*	0.3% Xyl	This work
B. subtilis KS69	*amyE::spec Pxyl-msfGFP-MreB*	-	(Scheinpflug *et al*, 2017)
B. subtilis HS549	*amyE::spec Pxyl-msfGFP-MreB bkd::ery des::kan*	1% Xyl	This work
B. subtilis HS547	*amyE::spec Pxyl-WALP23-mCherry bkd::ery des::kan*	1% Xyl	This work
B. subtilis JG054	*amyE::spec Pxyl-WALP23-msfGFP*	-	This work
B. subtilis HS552	*amyE::spec Pxyl-WALP23-msfGFP bkd::ery des::kan*	1% Xyl	This work
E. coli Y-Mel	λ^+ F$^+$ *mel-1 supF58* (wild type)	-	(Rickenberg & Lester, 1955)
E. coli MG1655	λ^+ F$^-$ *ilvG- rfb-50 rph-1* (wild type)	-	(Blattner *et al*, 1997)
E. coli AB1	F$^-$ *fhuA22 fabB15*(Ts) *fabF200 zcf-229*::Tn*10 gyrA220*(Nal^R) *rpsL146*(Sm^R) Δ*atpIBE*::FRT-*kan*-FRT (Kan^R)	-	This work
E. coli BHH87	F$^-$ *fhuA22 fabB15*(Ts) *fabF200 zcf-229*::Tn*10 gyrA220*(Nal^R) *rpsL146*(Sm) *atpB-mNeonGreen*	-	This work
E. coli BHH100	F$^+$ Sm^R *fabA*(Ts) *fabF* Crc$^-$ *ftsZ*^{55}-*msfGFP*-^{56}*ftsZ* (Cm^R)	-	This work
E. coli BHH101	F$^+$ Sm^R *fabA*(Ts) *fabF* Crc$^-$ *mreB*^{226}-*msfGFP*-^{227}*mreB csrD-mreB::*FRT-*kan*-FRT (Kan^R)	-	This work
E. coli CY288	F- *fhuA22 fabB15*(Ts) *fabF200 zcf-229*::Tn*10 gyrA220*(Nal^R) *rpsL146*(Sm^R)		(Garwin *et al*, 1980)
E. coli EB4	λ^+ F$^+$ *mel-1 supF58* Δ*atpI'BE'::kan*	-	(Renz *et al*, 2015)
E. coli EB8.1	λ^+ F$^+$ *mel-1 supF58 atpB-mCherry*	-	This work
E. coli JW0334-1	λ^- F$^-$ *rph-1 hsdR514* Δ*lacY784::*FRT-*kan*-FRT Δ*(araD-araB)567* Δ*lacZ4787*(::*rrnB-3*) Δ*(rhaD-rhaB)568* (Kan^R)	-	(Baba *et al*, 2006)
E. coli JW1087-2	λ^- F$^-$ *rph-1 hsdR514* Δ*ptsG763::*FRT-*kan*-FRT Δ*(araD-araB)567* Δ*lacZ4787*(::*rrnB-3*) Δ*(rhaD-rhaB)568* (Kan^R)	-	(Baba *et al*, 2006)
E. coli JW1165-1	λ^- F$^-$ *rph-1 hsdR514 ΔminC765::*FRT-*kan*-FRT Δ(*araD-araB*)567 Δ*lacZ4787*(::*rrnB-3*) Δ(*rhaD-rhaB*)568 (Kan^R)	-	(Baba *et al*, 2006)
E. coli JW2878-1	λ^- F$^-$ *rph-1 hsdR514 ΔzapA761::*FRT-*kan*-FRT Δ(*araD-araB*)567 Δ*lacZ4787*(::*rrnB-3*) Δ(*rhaD-rhaB*)568 (Kan^R)		(Baba *et al*, 2006)
E. coli JW3899-1	λ^- F$^-$ *rph-1 hsdR514 ΔzapB787::*FRT-*kan*-FRT Δ(*araD-araB*)567 Δ*lacZ4787*(::*rrnB-3*) Δ(*rhaD-rhaB*)568 (Kan^R)		(Baba *et al*, 2006)
E. coli KC555	λ^- F$^-$ *rph-1 ftsZ*^{55}-*msfGFP*-^{56}*ftsZ* (Cm^R)	-	(Yang *et al*, 2017)

E. coli LF4	F^+ Sm^R *fabA*(Ts) *fabF* Crc^- Δ*atpIBE::FRT-kan-FRT* (Kan^R)	-	This work
E. coli LF6.red	F^+ Sm^R *fabA*(Ts) *fabF* Crc^- *atpB-mCherry*	-	This work
E. coli MG1	λ^+ F^+ *mel-1 supF58 atpB-mNeonGreen*	-	This work
E. coli MG4	F^+ Sm^R *fabA*(Ts) *fabF* Crc^- *atpB-mNeonGreen*	-	This work
E. coli MG1655 mreB-msfGFP	λ^- F^- *rph-1 mreB*^{226}*-msfGFP-*^{227}*mreB csrD-mreB*::FRT-*kan*-FRT (Kan^R)	-	(Ursell *et al*, 2014)
E. coli UC1098	F^+ Sm^R *fabA*(Ts) *fabF* Crc^-	-	(Cronan *et al*, 1972)
E. coli UC1098.PtsG-mNG	F^+ Sm^R *fabA*(Ts) *fabF* Crc^- *ptsG-mNeonGreen*	-	This work
E. coli UC1098.Δ*ptsG*	F^+ Sm^R *fabA*(Ts) *fabF* Crc^- Δ*ptsG763::FRT-kan-FRT* (Kan^R)	-	This work
E. coli UC1098.Δ*lacY*	F^+ Sm^R *fabA*(Ts) *fabF* Δ*lacY784*::FRT-*kan*-FRT (Kan^R)	-	This work
E. coli UC1098.Δ*minC*	F^+ Sm^R *fabA(Ts) fabF* Crc^- Δ*minC765*::FRT-*kan*-FRT (Kan^R)	-	This work
E. coli UC1098.Δ*zapA*	F^+ Sm^R *fabA(Ts) fabF* Crc^- Δ*zapA761*::FRT-*kan*-FRT (Kan^R)		This work
E. coli UC1098.ΔzapB	F^+ Sm^R *fabA(Ts) fabF* Crc^- Δ*zapB787*::FRT-*kan*-FRT (Kan^R)		This work
E. coli Y-Mel.Δ*lacY*	λ^+ F^+ *mel-1 supF58 ΔlacY784::*FRT-*kan*-FRT (Kan^R)	-	This work

(Xyl, xylose)

Table S2. Detailed information on the lateral mobility of F_OF_1 *a*-mNG in *E. coli* WT cells and upon UFA depletion *in E. coli fabA*(Ts) cells analysed by single molecule tracking.

Strain / plasmid		30°C	30 → 33°C 120min	30 → 37°C 120min	30 → 40°C 120min
WT F_OF_1 *a*-mNG (MG1)	Cells	37 (8/6/15/8)	37 (13/10/14)	38 (13/8/10/7)	31 (7/11/13)
	T	2633	2970	2803	2345
	n/cell	74±18	78±23	72±22	75±21
fabA(Ts) F_OF_1 *a*-mNG (MG4)	Cells	31 (8/6/8/9)	47 (13/10/9/8/7)	33 (11/9/13)	38 (12/7/19)
	T	2588	4468	3088	2466
	n/cell	75±34	91±31	84±35	62±22

The number of cells analysed for jump distances in Fig 7 and Appendix Fig S13 is stated as sum and per replicate in brackets. T: sum of trajectories, n/cell: median count of trajectories per cell with SD.

Table S3. Detailed information on lateral mobility of transmembrane peptide WALP23-mNG upon UFA depletion in *E. coli fabA*(Ts) analysed by single molecule tracking.

Strain / plasmid		30°C	30 → 37°C 120min
fabA(Ts) WALP23-mNG (UC1098/pBH500)	Cells	30 (10/10/10)	30 (10/10/10)
	T	3305	2801
	n/cell	106±56	79±48

The number of cells analysed for jump distances in Appendix Fig S14 is stated as sum and per replicate in brackets. T: sum of trajectories, n/cell: median count of trajectories per cell with SD.

Table S4. Detailed information on osmotic stabilisation of lateral diffusion of F_OF_1 *a*-mNG in *E. coli* WT cells and upon UFA depletion in *E. coli fabB15*(Ts) analysed by single molecule tracking.

**Strain / plasmid**		**30°C - KCl**	**40°C - KCl**	**30°C + KCl**	**40°C + KCl**
WT F_OF_1 *a*-mNG (MG1)	Cells	47 (17/16/14)	50 (13/17/20)	76 (27/25/24)	75 (26/30/19)
	T	4860	7079	7490	7095
	n/cell	100±28	144±39	98±23	93±23
fabB(Ts) F_OF_1 *a*-mNG (BHH87)	Cells	81 (27/31/23)	60 (20/22/18)	73 (25/28/23)	88 (23/34/22)
	T	9334	5545	7542	5755
	n/cell	114±33	87±46	101±34	65±20

The number of cells analysed for jump distances in Appendix Fig S16 is stated as sum and per replicate in brackets. T: sum of trajectories, n/cell: median count of trajectories per cell with SD.

Table S5. Oligonucleotides

Oligo	Sequence (5‘ → 3‘)
1	GCA ACT TCT CCA ATG ATC TGA AG
2	GCA TAA CCG ATA CCG ACG ATC GG
3	CCG ATC GTC GGT ATC GGT TAT GCG
4	*ATC CTC CTC GCC CTT GCT CAC CAT* TCT AGA TGC CGG AGT TGG AGC CG
5	CGG CTC CAA CTC CGG CAT CTA GA*A TGG TGA GCA AGG GCG AGG AGG AT*
6	GTC TCC CCA ACG TCT TAC GGA TTA *CTT GTA CAG CTC GTC CAT GCC*
7	*GGC ATG GAC GAG CTG TAC AAG* TAA TCC GTA AGA CGT TGG GGA GAC
8	GTC AAT AAC CTG TTC GAC AAA ACC
9	GAA TTC TCA TGT TTG ACA GC
10	*GTT ATC CTC CTC GCC CTT GCT CAC CAT* GGA TCC ATG ATC TTC AGA CGC
11	GCG TCT GAA GAT CAT GGA TCC *ATG GTG AGC AAG GGC GAG GAG GAT AAC*
12	CGT AGT AGT GTT GGT AAA *TTA CTT GTA CAG CTC GTC CAT GCC C*
13	*CGG CAT GGA CGA GCT GTA CAA G*TA ATT TAC CAA CAC TAC TAC G
14	CGC TGG CCT TTG CAA GGT CAA GGT CCT TAT GTG CTC ATT CTG CGG
15	GGA GAT TCC TAG GAT GGC TTG GTG G
16	*GTT ATC CTC CTC GCC CTT GCT CAC CAT* TCC TGA GCC GCT TCC TGA CGC C
17	*GAT TAC GGC CTC TCC TTT GGA GAC CAT* TCC TGA GCC GCT TCC TGA CGC C
18	GGC GTC AGG AAG CGG CTC AGG A*AT GGT GAG CAA GGG CGA GGA GGA TAA C*
19	GCT CTA GAA CTA GTG GAT CTG AAG TCT GGA CAT *TTA CTT GTA CAG CTC GTC CAT GCC C*
20	GGC GTC AGG AAG CGG CTC AGG A*AT GGT CTC CAA AGG AGA GGC CGT AAT C*
21	GCT CTA GAA CTA GTG GAT CTG AAG TCT GGA CAT *TTA TTT ATA CAG CTC ATC CAT ACC AC*
22	CAT TCC TGA GCC GCT TC
23	GAC TTC AGA TCC ACT AGT TCT AGA G
24	CTA GAA CTA GTG GAT CTG AAG TC
25	AGC GGC TCA GGA ATG AGC AAA GGA GAA GAA CTT TTC
26	GTA CCC GGG GAT CCT C
27	CAT GGT GAA TTC CTC CTG C
28	GCA GGA GGA ATT CAC CAT GAA AAG AAT GTT AAT CAA CGC AAC
29	AGG ATC CCC GGG TAC TTA CTC AAC AGG TTG CGG AC
30	TTC TTC ACC ACC GCT TGC AGG AGC TGC TGG TG
31	AGC GGT GGT GAA GAA ACC AAA C

Deoxynucleotides corresponding to the genes encoding fluorescent proteins are in italics; restriction sites are underlined.

Table S6. Plasmids

Plasmid	Relevant Genotype	Induction	Source
pBH4	*ori*-ColE1 *bla* Δ*rop* (Ap^R) (pBR322) *Patp3-atpBEFHAGDC*	-	This work
pBH189	*ori*-ColE1 *bla* Δ*rop* (Ap^R) (pBR322) *Patp3-atpBEFHAGDC atpB-mNG*	-	This work
pBH500	*ori*-ColE1 *bla spc* (Ap^R Sp^R) (pSG1154) *amyE*::*Pxyl-WALP23-mNeonGreen*	-	This work
pBH501	*ori*-ColE1 *bla spc* (Ap^R Sp^R) (pSG1154) *amyE::Pxyl-WALP23-mScarlet-I*	-	This work
pBLP2	*ori*-ColE1 *bla* (Ap^R) (pBAD24) *araC ParaBAD-ptsG-GFP*	-	(Kosfeld & Jahreis, 2012)
pBWU13	*ori*-ColE1 *bla* (Ap^R) (pBR322) *Patp3-atpBEFHAGDC*	-	(Moriyama *et al*, 1991)
pEB21.2	*ori*-ColE1 *bla* (Ap^R) (pBR322) *Patp3-atpBEFHAGDC atpB-mCherry*	-	This work
pGI10	*ori*-ColE1 *bla* (Ap^R) *Ptrc-ompA-(LEDPPAEF)-mCherry*	0.1 mM IPTG	(Verhoeven *et al*, 2013)
pJG130	*ori*-ColE1 *bla* (Ap^R) (pBAD322) *ParaBAD-rne*	0.2% Ara	This work
pJG131	*ori*-ColE1 *bla* (Ap^R) (pBAD322) *ParaBAD-rne(*ΔAH*)*	0.2% Ara	This work
pKD4	*ori* ColE1 *bla FRT-kan-FRT* (Ap^R, Kan^R) (pANTSγ)	-	(Datsenko & Wanner, 2000)
pKD46	*ori-R101 repA101*(Ts) *bla* (Ap^R) *araC ParaBAD-gam-beta-exo*	0.2% Ara	(Datsenko & Wanner, 2000)
pL030	*ori*-ColE1 *bla spc* (Ap^R Sp^R) (pSG1154) *amyE::Pxyl-WALP23-mCherry*	-	S. Lee
pNCS-mNeonGreen	*ori*-ColE1 *bla* (Ap^R) (pNCS) *T7-tag His*$_6$*-mNG* (constitutive)	-	(Shaner *et al*, 2013)
pQW58	*ori*-ColE1 *bla* (Ap^R) *lacI*^{q} *Plac-mCherry*		(Galli & Gerdes, 2010)
pSD166	*ori*-p15A *bla* (Ap^R) (pACYC177) *Patp3-atpBEFHAGDC atpB-EGFP*	-	(Düser *et al*, 2008)
pSG1154	*ori*-ColE1 *bla spc amyE*'-'*amyE* (Ap^R $Spec^R$)	-	(Lewis & Marston, 1999)
pSTK3	*ori*-ColE1 *bla* (Ap^R) (pBR322) *Patp3-atpBEFHAGDC atpF(C21A)*	-	(Brandt *et al*, 2013)
pTM30.LacZ-His2	*ori*-ColE1 *bla* (Ap^R) *Ptac-OlacZ-lacZ-His2*	-	K. Jahreis
pVK207	*ori*-pSC101 *spc* (Sp^R) *Prne-rne-yfp*	-	(Khemici *et al*, 2008)

IPTG, isopropyl-β-D-thiogalactopyranoside; Ara, arabinose

References

Akamatsu Y (1974) Osmotic stabilization of unsaturated fatty acid auxotrophs of *Escherichia coli*. *J Biochem* 76: 553–61

Altabe SG, Aguilar P, Caballero GM, de Mendoza D (2003) The *Bacillus subtilis* acyl lipid desaturase is a Δ5 desaturase. *J Bacteriol* 185: 3228–3231

Baba T, Ara T, Hasegawa M, Takai Y, Okumura Y, Baba M, Datsenko KA, Tomita M, Wanner BL, Mori H (2006) Construction of *Escherichia coli* K-12 in-frame, single-gene knockout mutants: The KEIO collection. *Mol Syst Biol* 2: 2006.0008

Barbe V, Cruveiller S, Kunst F, Lenoble P, Meurice G, Sekowska A, Vallenet D, Wang T, Moszer I, Médigue C, Danchin A (2009) From a consortium sequence to a unified sequence: the *Bacillus subtilis* 168 reference genome a decade later. *Microbiology* 155: 1758–1775

Blattner FR, Plunkett III G, Bloch CA, Perna NT, Burland V, Riley M, Collado-Vides J, Glasner JD, Rode CK, Mayhew GF *et al* (1997) The complete genome sequence of *Escherichia coli* K-12. *Science* 277: 1453–1462

Brandt K, Maiwald S, Herkenhoff-Hesselmann B, Gnirß K, Greie JC, Dunn SD, Deckers-Hebestreit G (2013) Individual interactions of the *b* subunits within the stator of the *Escherichia coli* ATP synthase. *J Biol Chem* 288: 24465–24479

Broekman JH, Steenbakkers JF (1973) Growth in high osmotic medium of an unsaturated fatty acid auxotroph of *Escherichia coli* K-12. *J Bacteriol* 116: 285–289

Budin I, de Rond T, Chen Y, Chan LJG, Petzold CJ, Keasling JD (2018) Viscous control of cellular respiration by membrane lipid composition. *Science* 362: 1186–1189

Cronan Jr JE, Silbert DF, Wulff DL (1972) Mapping of the *fabA* locus for unsaturated fatty acid biosynthesis in *Escherichia coli*. *J Bacteriol* 112: 206–211

Datsenko KA, Wanner BL (2000) One-step inactivation of chromosomal genes in *Escherichia coli* K-12 using PCR products. *Proc Natl Acad Sci USA* 97: 6640–6645

Düser MG, Bi Y, Zarrabi N, Dunn SD, Börsch M (2008) The proton-translocating *a* subunit of F_OF_1-ATP synthase is allocated asymmetrically to the peripheral stalk. *J Biol Chem* 283: 33602–33610

Galli E, Gerdes K (2010) Spatial resolution of two bacterial cell division proteins: ZapA recruits ZapB to the inner face of the Z-ring. *Mol Microbiol* 76: 1514–1526

Garwin JL, Klages AL, Cronan Jr JE (1980) Structural, enzymatic, and genetic studies of b-ketoacyl-carrier protein synthases I and II of *Escherichia coli. J Biol Chem* 255: 11949–11056

Hachmann AB, Angert ER, Helmann JD (2009) Genetic analysis of factors affecting susceptibility of *Bacillus subtilis* to daptomycin. *Antimicrob Agents Chemother* 53: 1598–1609

Khemici V, Poljak L, Luisi BF, Carpousis AJ (2008) The RNase E of *Escherichia coli* is a membrane-binding protein. *Mol Microbiol* 70: 799–813

Kosfeld A, Jahreis K (2012) Characterization of the interaction between the small regulatory peptide SgrT and the EIICBGlc of the glucose-phosphotransferase system of *E. coli* K-12. *Metabolites* 2:

756–774

Lewis PJ, Marston AL (1999) GFP vectors for controlled expression and dual labelling of protein fusions in *Bacillus subtilis*. *Gene* 227: 101–110

Mercier R, Dominguez-Cuevas P, Errington E (2012) Crucial role for membrane fluidity in proliferation of primitive cells. *Cell Rep* 1: 417–423

Moriyama Y, Iwamoto A, Hanada H, Maeda M, Futai M (1991) One-step purification of *Escherichia coli* H^+-ATPase (F_OF_1) and its reconstitution into liposomes with neurotransmitter transporters. *J Biol Chem* 266: 22141–22146

Olsen JG, Kadziola A, von Wettstein-Knowles P, Siggaard-Andersen M, Lindquist Y, Larsen S (1999) The X-ray crystal structure of β-ketoacyl [acyl carrier protein] synthase I. *FEBS Lett* 460: 46**–**52

Renz A, Renz M, Klütsch D, Deckers-Hebestreit G, Börsch M (2015) 3D-localisation microscopy and tracking of F_OF_1-ATP synthases in living bacteria. *Proc SPIE* 9331: 93310D

Rickenberg HV, Lester G (1955) The preferential syntheis of β-galactosidase in *Escherichia coli*. *J Gen Microbiol* 13: 279–284

Scheinpflug K, Wenzel M, Krylova O, Bandow JE, Dathe M, Strahl H (2017) Antimicrobial peptide cWFW kills by combining lipid phase separation with autolysis. *Sci Rep* 7: 44332

Shaner NC, Lambert GG, Chammas A, Ni Y, Cranfill PJ, Baird MA, Sell BR, Allen JR, Day RN, Israelsson M *et al* (2013) A bright monomeric green fluorescent protein derived from *Branchiostoma lanceolatum*. *Nat Methods* **10**: 407–409

Strahl H, Hamoen LW (2010) Membrane potential is important for bacterial cell division. *Proc Natl Acad Sci USA* 107: 12281–12286

Stokes NR, Sievers J, Barker S, Bennett JM, Brown DR, Collins I, Errington VM, Foulger D, Hall M, Halsey R *et al* (2005) Novel inhibitors of bacterial cytokinesis identified by a cell-based antibiotic screening assay. *J Biol Chem* 280: 39709–39715

Ursell TS, Nguyen J, Monds RD, Colavin A, Billings G, Ouzounov N, Gitai Z, Shaevitz JW, Huang KC (2014). Rod-like bacterial shape is maintained by feedback between cell curvature and cytoskeletal localisation. *Proc Natl Acad Sci USA* 111: E1025–1034

Verhoeven GS, Dogterom M, den Blaauwen T (2013) Absence of long-range diffusion of OmpA in *E. coli* is not caused by its peptidoglycan binding domain. *BMC Microbiol* 13: 66

Yang X, Lyu Z, Miguel A, McQuillen R, Huang KC, Xiao J (2017) GTPase activity-coupled treadmilling of the bacterial tubulin FtsZ organizes septal cell wall synthesis. *Science* 355: 744–747
