# Supplementary material for: Low membrane fluidity triggers lipid phase separation and protein segregation in living bacteria
Source: EMBO J. 2022 Jan 17;41(5):e109800. doi: 10.15252/embj.2021109800 (PMC8886542; doi:10.15252/embj.2021109800)
Supplement: Supplementary file 2 — Expanded View Figures PDF [file EMBJ-41-e109800-s013.pdf]

# Expanded View Figures

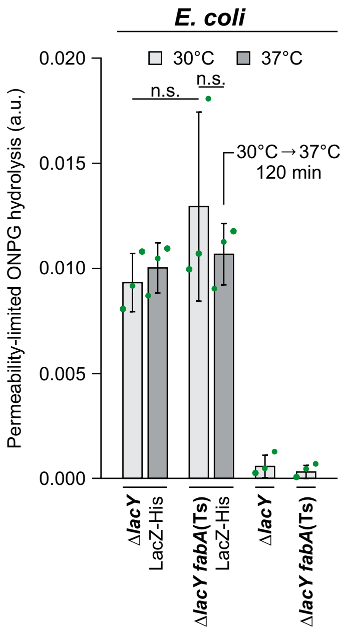


**Figure EV1. Very low membrane fluidity in *E. coli* does not trigger membrane permeabilisation for *ortho*-nitrophenyl β-D-galactopyranoside (ONPG).**

The membrane permeability of ONPG was assessed in strains deficient for the active uptake system LacY simultaneously expressing *lacZ* from a plasmid-encoded leaky *Ptac* promoter (without addition of the inducer IPTG). The graph depicts ONPG hydrolysis rates measured for intact cells upon incubation at 30 and 37°C. In case of the temperature-sensitive *fabA*(Ts) strain, growth at the non-permissive temperature of 37°C was limited to 120 min. As controls, the ONPG conversion rates were measured in strains lacking the *lacZ*-expressing plasmid. Note the lack of significant difference in ONPG conversion rates upon strong depletion of unsaturated fatty acids (*fabA*(Ts) strain at 37°C for 120 min), implying the lack of detectable membrane permeabilisation due to phase separation.

Data information: The graph depicts mean and SD of biological triplicates. The *P* values represent the results of unpaired, two-sided *t*-tests. Insignificant changes ($P > 0.1$) are indicated with n.s. Strains used: *E. coli* Y-Mel.Δ*lacY*, UC1098.Δ*lacY*, Y-Mel.Δ*lacY*/pTM30.*lacZ*-His2, UC1098.Δ*lacY*/pTM30.*lacZ*-His2. Source data are available online for this figure.

 © 2022 The Authors

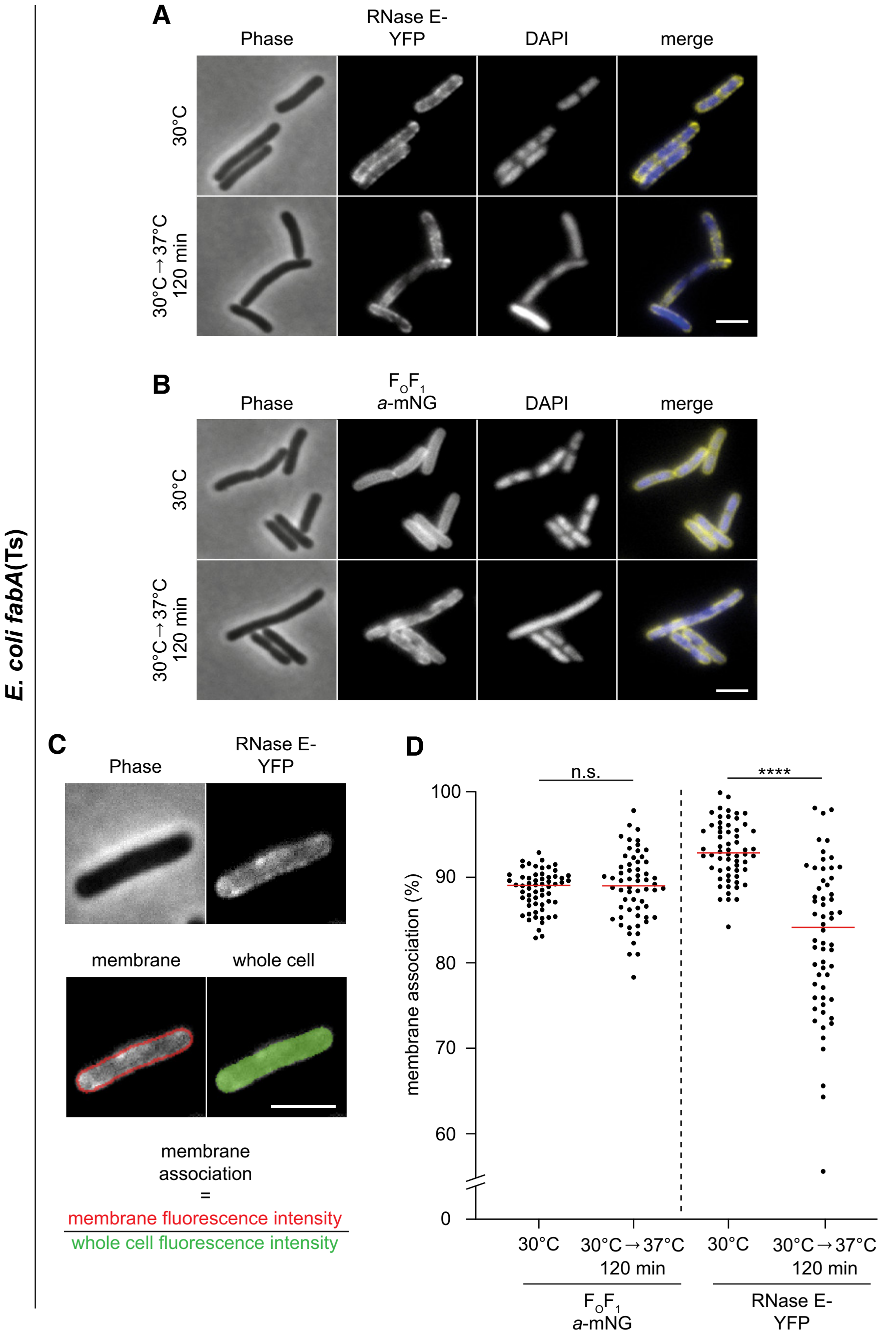


**Figure EV2. Very low membrane fluidity triggers partial dissociation of RNase E from the membrane in *E. coli fabA*(Ts).**

A, B Phase-contrast and fluorescence images of *E. coli fabA*(Ts) strain expressing (A) RNase E-YFP or (B) $F_OF_1$ *a*-mNG. Cells were grown in LB at 30°C to an $OD_{600}$ of 0.3, transferred to the non-permissive temperature of 37°C for 120 min followed by labelling with DAPI and fluorescence microscopy. Note the increasing cytoplasmic localisation of RNase E-YFP upon depletion of the membrane for UFA, which coincides with decondensation of the nucleoid (compare Fig 4B).

C Quantification of membrane association of RNase E. The degree of membrane association was quantified by automated detection of cells using phase-contrast images, defining a 3-pixel wide band around the periphery of the cell, and measuring the relative membrane association as a ratio between the mean peripheral fluorescence signal and the mean fluorescence of the whole cell.

D Relative membrane association of $F_OF_1$ *a*-mNG and RNase E-YFP at 30°C and 37°C for 120 min in individual cells ($n = 60$). Red lines indicate the median.

Data information: (A–D) Experiments are representative of independent biological duplicates. (D) Red lines indicate the median. *P* values represent the results of unpaired, two-sided *t*-tests. Significance was assumed with ****$P < 0.0001$, n.s., not significant (A–C). Scale bar: 3 μm. Strains used: (A, C, D) *E. coli* UC1098/pVK207; (B, D) *E. coli* MG4. Source data are available online for this figure.

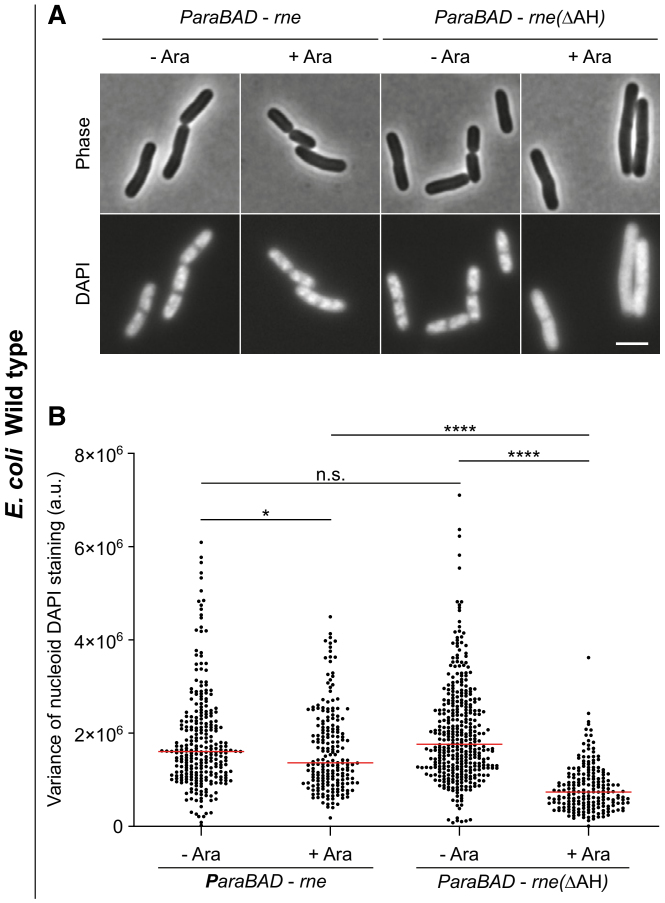


**Figure EV3. Expression of cytoplasmic RNase E is sufficient to trigger decondensation of the nucleoid.**

A Phase-contrast and fluorescence images of *E. coli* WT cells expressing plasmid-encoded full-length membrane-associated RNase E (Rne) and a corresponding construct encoding RNase E that lacks the membrane-binding amphipathic helix (RneΔAH), respectively. The cells depicted were grown in LB medium at 37°C to an $OD_{600}$ of 0.3 followed by induction of *rne* with 0.2% (w/v) arabinose (Ara) for 60 min, labelling with DAPI for 15 min and fluorescence microscopy. Note the decondensation of the nucleoid observed upon expression of cytoplasmically located RneΔAH, but not in the presence of the native membrane-associated RNase E.

B Variance of nucleoid staining. The degree of nucleoid condensation was assessed by analysing the variance of DAPI fluorescence within the cell ($n$ = 199–372). In this type of analysis, a more homogeneous fluorescence signal such as that caused by nucleoid decondensation results in a lower variance of the per pixel fluorescence intensity. Red lines indicate the median.

Data information: (A, B) The experiments are representative of biological duplicates. (B) Red lines indicate the median. *P* values represent results of unpaired, two-sided *t*-tests. Significance was assumed with ****$P < 0.0001$, *$P < 0.05$, n.s., not significant. (A) Scale bar: 3 μm. Strains used: (A, B) *E. coli* MG1655/pJG130, MG1655/pJG131.
Source data are available online for this figure.

 © 2022 The Authors

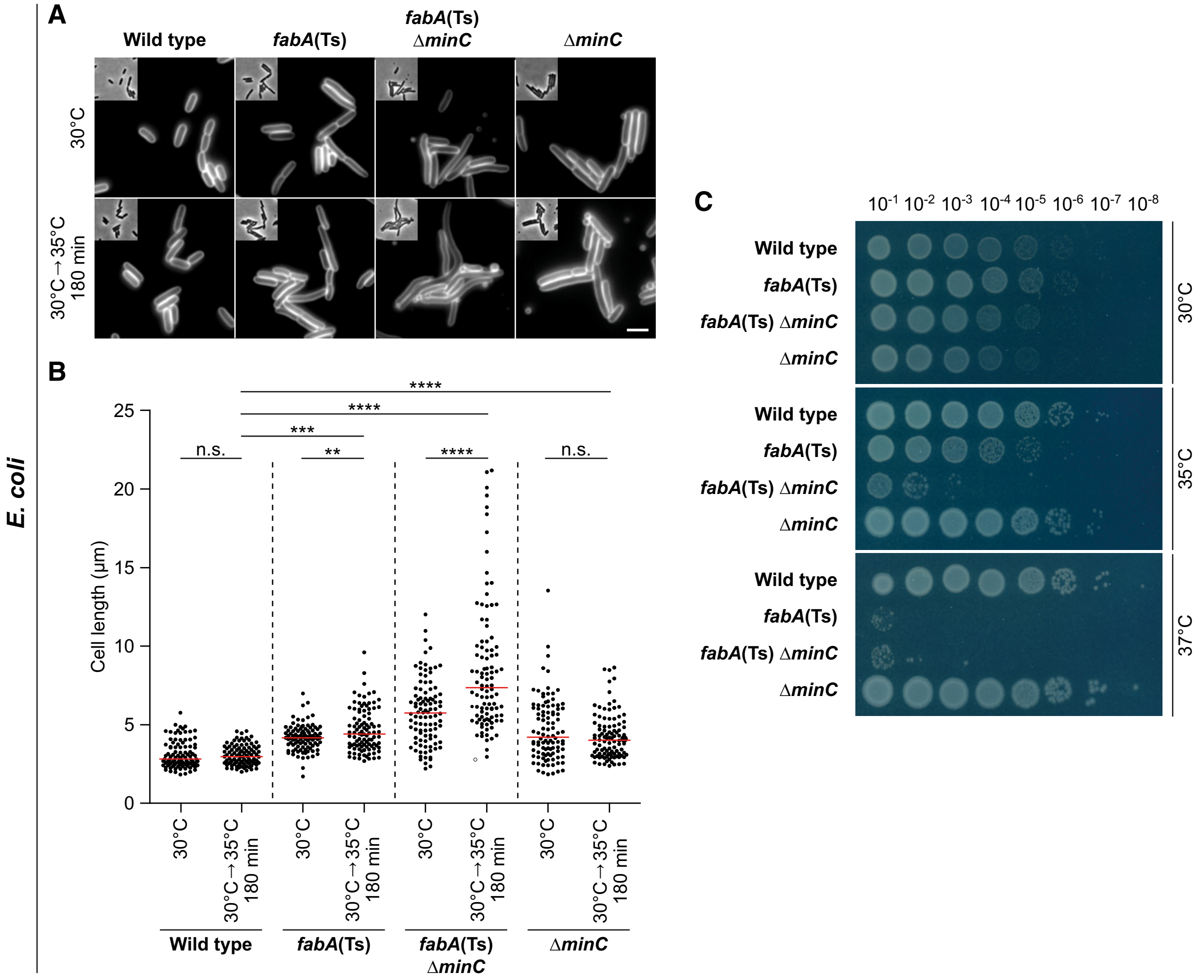


**Figure EV4. Destabilisation of *E. coli* divisome by deletion of the division regulator *minC* triggers hypersensitivity towards low membrane fluidity.**

A Images of *E. coli* WT, *fabA*(Ts), *fabA*(Ts) Δ*minC and* Δ*minC* cells grown either at the permissive temperature (30°C) or for 180 min at 35°C, which is non-permissive for the *fabA*(Ts) Δ*minC* strain. Cells were stained with the outer membrane dye FM 5–95 prior to microscopy. Note the strong cell elongation of the *fabA*(Ts) Δ*minC* strain upon incubation at 35°C, which is indicative of a severe cell division defect.

B Quantification of cell length for cells ($n = 100$) depicted in panel A. Red lines indicate the median.

C Viability of the strains depicted in panel A upon incubation on agar plates in M9-glucose minimal medium overnight at different temperatures. The serial dilutions and spot assays were carried out with pre-cultures grown at 30°C to mid-log growth phase. Note the temperature hypersensitivity and loss of viability of strain *fabA* (Ts) Δ*minC* at 35°C, which indicates that the cell division process has become the limiting factor in tolerance towards low membrane fluidity in this strain. The temperature sensitivity of the strains *fabA*(Ts) Δ*zapA* and *fabA*(Ts) Δ*zapB* supported this view (see Appendix Fig S5).

Data information: (A–C) The experiments are representative of biological triplicates. (B) Red lines indicate the median, while the *P* values represent results of unpaired, two-sided *t*-tests. Significance was assumed with ****$P < 0.0001$, ***$P < 0.001$, **$P < 0.01$, n.s., not significant. (A) Scale bar: 3 µm. Strains used: (A–C) *E. coli* Y-Mel, UC1098, UC1098.Δ*minC*, JW1165-1.
Source data are available online for this figure.

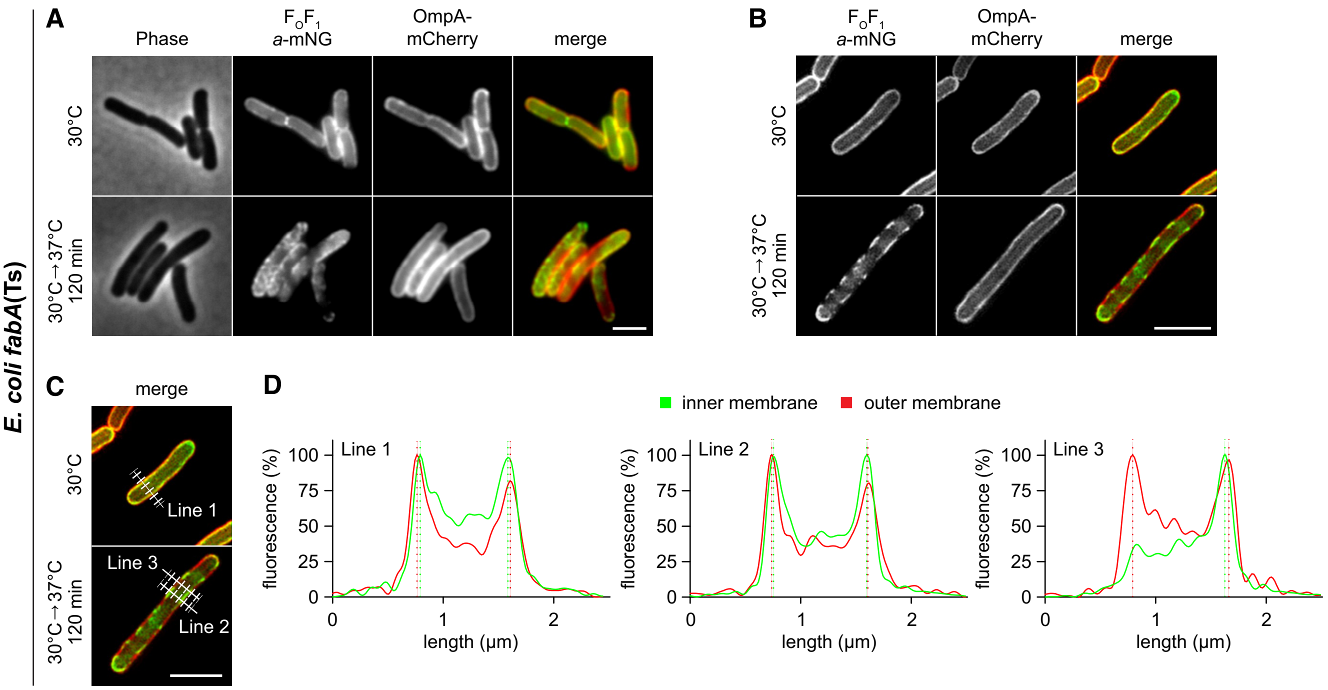


**Figure EV5. Protein segregation induced by very low membrane fluidity is limited to the inner cytoplasmic membrane in *E. coli*.**

A Membrane protein segregation was monitored in *fabA*(Ts) cells expressing as inner membrane marker $F_OF_1$ *a*-mNG and as outer membrane marker OmpA-mCherry. Widefield microscopy images depict phase-contrast, fluorescence and overlay images for cells grown at permissive 30°C or at non-permissive 37°C for 120 min in M9-glucose minimal medium. Note the transition from disperse localisation into a segregated pattern in case of the inner membrane-localised $F_OF_1$ *a*-mNG at 37°C, while the pattern of the outer membrane-localised OmpA-mCherry remains homogeneous at both growth temperatures.

B Super-resolution 2D-SIM (structured illumination microscopy) images of cells expressing $F_OF_1$ *a*-mNG and OmpA-mCherry at both growth temperatures.

C Localisation and orientation of 5-pixel wide lines used to analyse fluorescence intensity profiles depicted in panel D.

D Fluorescence intensity line scans across the cells imaged with 2D-SIM microscopy. Note the small, but detectable outward shift between the inner membrane marker $F_OF_1$ *a*-mNG and outer membrane marker OmpA-mCherry.

Data information: (A–D) Experiments are representative of biological triplicates (A–C). Scale bar: 3 μm. Strain used: (A–D) *E. coli* MG4/pGI10.
Source data are available online for this figure.

 © 2022 The Authors
