# Supplementary material for: Low membrane fluidity triggers lipid phase separation and protein segregation in living bacteria
Source: EMBO J. 2022 Jan 17;41(5):e109800. doi: 10.15252/embj.2021109800 (PMC8886542; doi:10.15252/embj.2021109800)
Supplement: Supplementary file 3 — Movie EV1 [file EMBJ-41-e109800-s006.zip › Movie EV1 legend.docx]

Movie EV1: Time lapse microscopy of fatty acid precursor auxotroph *B. subtilis* Δ*bkd* strain depleted for branched chain fatty acids.

Cells labelled with membrane dye FM 5-95 were grown in the presence of precursor IB, washed precursor-free (PF) and transferred to time lapse slides prepared with PF medium or IB-supplemented medium.

Data information: Cells were imaged for 250 min at 5 min intervals and 350 ms exposure time per frame. The movie frame rate is 5 frames per second. Scale bar, 3 µm. Strain used: *B. subtilis* HS527.
