# Supplementary material for: Low membrane fluidity triggers lipid phase separation and protein segregation in living bacteria
Source: EMBO J. 2022 Jan 17;41(5):e109800. doi: 10.15252/embj.2021109800 (PMC8886542; doi:10.15252/embj.2021109800)
Supplement: Supplementary file 4 — Movie EV2 [file EMBJ-41-e109800-s008.zip › Movie EV2 legend.docx]

**Movie EV2: Time lapse microscopy of the thermosensitive *E. coli* *fabA*(Ts) strain depleted for unsaturated fatty acids.**

Cells chromosomally expressing mNG-labelled ATP synthase (F_O_F_1_ *a*-mNG) were grown at 30°C, transferred to time lapse slides with the same medium and grown at permissive 30°C or at non-permissive 40°C for depletion of UFA.

Data information: Cells were imaged for 150 min in 5 min intervals and 50 ms exposure time per frame. The movie frame rate is 5 frames per second. Deconvolution (only for the merge frames) was performed using SoftWoRx software. Scale bar, 2 µm. Strain used: MG4.
