# Supplementary material for: Low membrane fluidity triggers lipid phase separation and protein segregation in living bacteria
Source: EMBO J. 2022 Jan 17;41(5):e109800. doi: 10.15252/embj.2021109800 (PMC8886542; doi:10.15252/embj.2021109800)
Supplement: Supplementary file 5 — Movie EV3 [file EMBJ-41-e109800-s012.zip › Movie EV3 legend.docx]

**Movie EV3: Trajectory maps of *in vivo* single molecule tracking of mNG-labelled ATP synthase (F_O_F_1_ *a*-mNG) in *E. coli* wild type cells.**

Cells chromosomally expressing F_O_F_1_ *a*-mNG were grown at 30°C or grown at 30°C and shifted to 33°C, 37°C or 40°C for 120 min as indicated.

Data information: The movie shows sequential frames with a frame binning of 2 and 15 frames per second. All trajectories of F_O_F_1_ *a*-mNG complexes with ≥5 consecutive frames are shown. Scale bar, 1 µm. Strain used: *E. coli* MG1.
