# Supplementary material for: Low membrane fluidity triggers lipid phase separation and protein segregation in living bacteria
Source: EMBO J. 2022 Jan 17;41(5):e109800. doi: 10.15252/embj.2021109800 (PMC8886542; doi:10.15252/embj.2021109800)
Supplement: Supplementary file 13 [file EMBJ-41-e109800-s007.pdf]

THE EMBO JOURNAL

Low membrane fluidity triggers lipid phase separation and protein segregation in living bacteria

Marvin Gohrbandt, André Lipski, James Grimshaw, Jessica Buttress, Zunera Baig, Brigitte Herkenhoff, Stefan Walter, Rainer Kurre, Gabriele Deckers-Hebestreit, and Henrik Strahl
DOI: 10.15252/embj.2021109800

Corresponding author(s): Henrik Strahl (h.strahl@ncl.ac.uk) , Gabriele Deckers-Hebestreit (gabriele.deckers-hebestreit@uni-osnabrueck.de)

Review Timeline:

Submission Date:	24th Sep 21
Editorial Decision:	2nd Nov 21
Revision Received:	4th Dec 21
Editorial Decision:	15th Dec 21
Revision Received:	19th Dec 21
Accepted:	21st Dec 21

Editor: Stefanie Boehm

Transaction Report:

(Note: With the exception of the correction of typographical or spelling errors that could be a source of ambiguity, letters and reports are not edited. Depending on transfer agreements, referee reports obtained elsewhere may or may not be included in this compilation. Referee reports are anonymous unless the Referee chooses to sign their reports.)

Dr. Henrik Strahl
Newcastle University
Centre for Bacterial Cell Biology, Biosciences Institute
Faculty of Medical Sciences
Baddiley-Clark Bldg., Richardson Road
Newcastle upon Tyne NE2 4AX
United Kingdom

2nd Nov 2021

Re: EMBOJ-2021-109800
Low membrane fluidity triggers lipid phase separation and protein segregation in vivo

Dear Dr. Strahl,

Thank you for submitting your manuscript assessing membrane fluidity in bacterial models to The EMBO Journal. We have now received three referee reports on your study, which are included below for your information. In light of these comments, we would like to invite you to prepare and submit a revised manuscript.

As you will see, the reviewers appreciate the analysis and acknowledge the interest to the field. Overall, most raised issues can likely be resolved by textual changes, additional explanations and/or revision of the figures. However, referee #3 does raise one point that should be addressed in more detail, namely in which context such changes in membrane fluidity become more critical. This point should be discussed in further detail and experimental data addressing the question added if available. In addition to revising the manuscript and figures as appropriate, please also remember to provide a detailed response to each comment when submitting the revised manuscript. We also encourage authors to include all relevant information on materials and methods in the main manuscript, so please consider moving this section from the Appendix to the main text (there are no page limits). Please also refer to the submission guidelines for revisions (details below and https://www.embopress.org/page/journal/14602075/authorguide#submissionofrevisions) (i.e. regarding the upload of EV figures).

Please note that it is our policy to allow only a single round of major revision. Acceptance depends on a positive outcome of a second round of review and therefore on the completeness of your responses included in the next, final version of the manuscript. Please contact me to discuss any uncertainties regarding specific points or if you have any additional questions regarding this revision. Thank you for the opportunity to consider your work for publication. I look forward to receiving your revised manuscript.

Kind regards,

Stefanie Boehm

Stefanie Boehm
Editor
The EMBO Journal

Referee #1:

Review on the manuscript , Low membrane fluidity triggers lipid phase separation and
protein segregation in vivo'

Using elegant genetic and metabolic perturbation experiments the authors re-engineer the lipid acyl chain composition in Bacillus subtilis and Escherichia coli and study various aspects of membrane biophysics in living cells. In B. subtilis the authors interfere with the production of unsaturated fatty acids and branched chain fatty acids thereby rendering the cells fatty acid precursor-auxotrophic. In E. coli, the authors use a previously isolated, temperature-sensitive fabF fabA(Ts) variant to target the ratio of unsaturated to saturated fatty acids. Even though the approach of combining genetic and metabolic perturbation is not entirely novel, the re-engineered bacterial membranes are characterized with a unique combination of state-of-the art lipidomic analyses, fluorescent membrane probes and tracking experiments. The finding that a perturbed lipid composition in bacteria induces lateral membrane heterogeneities and lipid phase separation justifies a publication in EMBO J. This study is important, because it directly tackles the general misconception that gel phase formation is not acceptable for life. The technical quality of the data and their representation are of highest standards. The manuscript is clearly written and certainly of great interest for the broad readership of EMBO J.

I have reviewed this study previously (for another journal) and was impressed with the responsiveness of the authors to my own critique as well as the critique raised by two additional reviewers. Because I see that all my previous comments have been addressed in the current manuscript, I can strongly recommend this manuscript for publication in EMBO J.

I am sure that this very well executed study will draw a lot of attention.

Referee #2:

The manuscript by Gohrbrandt and colleagues describes a detailed analysis of membrane fluidity adaption in two model bacteria, Escherichia coli and Bacillus subtilis. The authors started with the construction of strains in which either branched chain fatty acid (BCFA) synthesis is deleted in B. subtilis (here they deleted the genes bkd and des) and they created an E. coli strain that can be depleted for unsaturated fatty acids (UFA) by mutating fabA. This mutation renders FabA temperature sensitive. With these two strains in hand, the authors have an elegant model to modulate the lipid composition (and in turn the membrane fluidity). Both strains grow fine when either supplemented with correct fatty acid precursors or at permissive temperature. Using the anisotropic dye DPH they go on and show that depletion of BCFAs or UFAs indeed translates into an altered membrane fluidity as expected. Therefore, these strains can be used to study influence of membrane fluidity on membrane associated processes in a controlled and importantly reversible manner in vivo.
The authors make some key findings. First, they show that the membrane integrity is remarkably robust to changes in fluidity and membrane potential. They also show that several key cellular functions such as cell division and elongation growth are affected. This part of the paper remains somewhat descriptive, but the findings are striking and merit to be inserted in the paper. A more important part is the fact that membrane homogeneity is affected and a segregation of membranes into gel and fluid

phases can be observed in vivo, resulting in a nice example of a phase separation in vivo (that surprisingly does not lead to cell death). Dynamics of membrane integral protein complexes was tested by using mNeonGreen fused ATP synthase (FoF1). Analysis using single-molecule tracking revealed that unrestricted lateral mobility of FoF1 under wild type conditions. However, under UFA depletion conditions, lateral movement of the enzyme complex was reduced. Osmotic stabilization using potassium somehow reduced the degree of partitioning.
The authors conclude that the changes in single-molecule dynamics are reflecting the separation into different membrane environments (exclusion from gel phase, as depicted in their model shown in figure 8. Although this is a plausible and attractive model, I am not fully convinced that the data can really show this. A redistribution, or better exclusion of proteins from gel phases will decrease the space in which the protein can be dynamic, but it does not necessarily mean that the diffusion coefficient needs to changes. Just the confinement radius will be different. It would be good if the model could be discussed in more detail. Generally, when talking about demixing of lipids in experiments where only proteins are looked at is difficult. This does not mean that the entire study needs to be repeated with lipid tracking (which would be difficult anyway). However, a slightly more detailed discussion would help the reader to understand the limitations.
This paper has likely undergone some serious revision before this submission, as a first version was preprinted on biorxiv in November 2019. I did not review any previous versions of this manuscript, but I realized a considerable improvement when comparing the manuscripts. Thus, this paper is already in a very good shape for publication and it is an important contribution to our understanding of membrane organization and in particular to membrane fluidity adaption. I have therefore summarized only a few minor points that the authors might want to address.

Points to consider:
The single molecule tracking data are quite convincing. However, the data presentation could be optimized, even without additional experimental work.
Jump size is typically referred to as jump distance, maybe the authors consider rephrasing.
The Cumulative Probability distribution (7b) is not very self-explanatory compared to typical SPT Plots (e.g. 1. Probability (y) vs jump distance (x) or 2. prob. Density (y) vs. Diffusion coefficient). These type of plots can be used to show the fitting functions.
I am not sure whether in the analyses shown in figure 7b the jump distance is one dimensionally or is the Cumulative probability actually plotted against a squared Jump size (nm^2) and just annotated wrongly?
Even though I am generally convinced by the results displayed in Figure 7, showing the apparent diffusion as a single median (= single population, 7c) might be masking a second population arising in the fabA strain under UFA depletion conditions. Since the membrane is not expected to be homogeneously affected by the increased rigidity/decreased fluidity, not all tracks are expected to be slower compared to the WT. Instead, the range between fast and slow tracks should also increase, which could make a fit with 2 populations more suitable. According to figure S12, this is not the case, or could 33ms frames be too slow to capture very fast molecules?
Why are the data shows Fig 7c as a barplot? Would a boxplot not be the more precise visualization to display the range of the cell-to-cell Dapp median values?
Supplement to Fig. 7: how many tracks were finally utilized per condition for analysis and statistics?
How does the measured diffusion coefficient (appD in µm2/s) compare to other values measured for membrane proteins in the literature?
I have a general question about strain construction. Many strains used here (such as the PtsG-mNG) are expressed from plasmids. Is it confirmed that the fluorescent fusions are functional? Also, did the author check for stability of their fluorescent fusions (by western blotting)?
The authors used polymyxin B to permeabilize the outer membrane of E. coli for the DPH measurements. Could polymyxin B treatment have an effect on the inner membrane?
In Figure EV4 the authors show the hypersensitivity of a fabA(TS) combined with a minC deletion. The authors conclude that cell division becomes growth limiting upon UFA depletion in E. coli. The argumentation is not really clear to me. A minC deletion will lead to an increase in cell length. Could this lead to an increase in sensitivity? Are longer cells in general more sensitive to fluidity changes? What happens in Bacillus if for example DivIVA is deleted and the cells get filamentous?

Referee #3:

In their manuscript entitled 'Low membrane fluidity triggers lipid phase separation and protein segregation in vivo', Gohrbandt and coworkers study the effect of decreasing membrane fluidity (by manipulating membrane lipid composition) in E. coli and B. subtilis. They set up systems to acutely deplete branched chain fatty acids and unsaturated fatty acids from B. subtilis and E. coli lipids respectively which induces a reduction in their membrane fluidity. Following these treatments, they observe membrane depolarization, disturbed localization of membrane-associated cellular factors, membrane phase separation with consequent segregation of transmembrane proteins in the formed domains. The manuscript is well written, the data are of good quality, and the authors' interpretation of the data is justified by the evidence provided. The central question about the cellular consequences of inadequate membrane fluidity is important and the data obtained by the authors are an important advancement towards addressing this issue.

Nonetheless, the observed phenotypes are obtained by changing membrane fluidity by an extent way bigger than that dealt by the cells when reacting to temperature changes during homeoviscous adaptation. Thus, as the authors state "while both E. coli and B. subtilis adapt their membrane composition and fluidity even upon subtle changes in temperature, the failure to do so is not associated with immediate growth-inhibitory consequences". This evidence triggers the question of what selective pressure has operated on these organisms to shape a metabolic rewiring aimed at adjusting subtle changes in membrane fluidity.

In my opinion, the authors should provide at least a perspective explanation for this by subjecting bacteria with mildly affected membrane fluidity to challenges they might encounter in their natural environment (phage intoxication, interaction with antibacterial peptides or antibiotics) and by looking at their capability to adapt to their environment (i.e., to transition from a motile planktonic cell state to a sessile biofilm state).

Referee #1:

Review on the manuscript , Low membrane fluidity triggers lipid phase separation and protein segregation in vivo'

Using elegant genetic and metabolic perturbation experiments the authors re-engineer the lipid acyl chain composition in Bacillus subtilis and Escherichia coli and study various aspects of membrane biophysics in living cells. In B. subtilis the authors interfere with the production of unsaturated fatty acids and branched chain fatty acids thereby rendering the cells fatty acid precursor-auxotrophic. In E. coli, the authors use a previously isolated, temperature-sensitive fabF fabA(Ts) variant to target the ratio of unsaturated to saturated fatty acids. Even though the approach of combining genetic and metabolic perturbation is not entirely novel, the re-engineered bacterial membranes are characterized with a unique combination of state-of-the art lipidomic analyses, fluorescent membrane probes and tracking experiments. The finding that a perturbed lipid composition in bacteria induces lateral membrane heterogeneities and lipid phase separation justifies a publication in EMBO J. This study is important, because it directly tackles the general misconception that gel phase formation is not acceptable for life. The technical quality of the data and their representation are of highest standards. The manuscript is clearly written and certainly of great interest for the broad readership of EMBO J.

I have reviewed this study previously (for another journal) and was impressed with the responsiveness of the authors to my own critique as well as the critique raised by two additional reviewers. Because I see that all my previous comments have been addressed in the current manuscript, I can strongly recommend this manuscript for publication in EMBO J.

I am sure that this very well executed study will draw a lot of attention.

We would like to thank the reviewer for the very positive evaluation of our manuscript, and for the previous review as well.

Referee #2:

The manuscript by Gohrbrandt and colleagues describes a detailed analysis of membrane fluidity adaption in two model bacteria, Escherichia coli and Bacillus subtilis. The authors started with the construction of strains in which either branched chain fatty acid (BCFA) synthesis is deleted in B. subtilis (here they deleted the genes bkd and des) and they created an E. coli strain that can be depleted for unsaturated fatty acids (UFA) by mutating fabA. This mutation renders FabA temperature sensitive. With these two strains in hand, the authors have an elegant model to modulate the lipid composition (and in turn the membrane fluidity). Both strains grow fine when either supplemented with correct fatty acid precursors or at permissive temperature. Using the anisotropic dye DPH they go on and show that depletion of BCFAs or UFAs indeed translates into an altered membrane fluidity as expected. Therefore, these strains can be used to study influence of membrane fluidity on membrane associated processes in a controlled and importantly reversible manner in vivo.

The authors make some key findings. First, they show that the membrane integrity is remarkably robust to changes in fluidity and membrane potential. They also show that several key cellular functions such as cell division and elongation growth are affected. This part of the paper remains somewhat descriptive, but the findings are striking and merit to be inserted in the paper. A more

important part is the fact that membrane homogeneity is affected and a segregation of membranes into gel and fluid phases can be observed in vivo, resulting in a nice example of a phase separation in vivo (that surprisingly does not lead to cell death). Dynamics of membrane integral protein complexes was tested by using mNeonGreen fused ATP synthase (FoF1). Analysis using single-molecule tracking revealed that unrestricted lateral mobility of FoF1 under wild type conditions. However, under UFA depletion conditions, lateral movement of the enzyme complex was reduced. Osmotic stabilization using potassium somehow reduced the degree of partitioning.

The authors conclude that the changes in single-molecule dynamics are reflecting the separation into different membrane environments (exclusion from gel phase, as depicted in their model shown in figure 8. Although this is a plausible and attractive model, I am not fully convinced that the data can really show this. A redistribution, or better exclusion of proteins from gel phases will decrease the space in which the protein can be dynamic, but it does not necessarily mean that the diffusion coefficient needs to changes. Just the confinement radius will be different. It would be good if the model could be discussed in more detail. Generally, when talking about demixing of lipids in experiments where only proteins are looked at is difficult. This does not mean that the entire study needs to be repeated with lipid tracking (which would be difficult anyway). However, a slightly more detailed discussion would help the reader to understand the limitations.

> It is indeed correct that the overcrowding of membrane proteins makes it challenging to differentiate between a reduction of D_{app} in the fluid phase and reduction in dynamic space due to confinement in the remaining fluid phase areas, in which the proteins accumulate. However, the observed displacements appear to be significantly smaller than the remaining fluid phase membrane areas observed by epifluorescence microscopy (compare Fig 5C and E and 6A-D). This argues against confinement as the sole reason for the reduced diffusion dynamics observed. Furthermore, a reduction of the diffusion coefficient would also be consistent with previous *in vitro* work demonstrating a linear decrease of both protein and lipid lateral mobility with increasing membrane protein concentrations (1). We have now added the discussion of this important point in lines 368-378 of the manuscript.

This paper has likely undergone some serious revision before this submission, as a first version was preprinted on biorxiv in November 2019. I did not review any previous versions of this manuscript, but I realized a considerable improvement when comparing the manuscripts. Thus, this paper is already in a very good shape for publication and it is an important contribution to our understanding of membrane organization and in particular to membrane fluidity adaption. I have therefore summarized only a few minor points that the authors might want to address.

> We would like to thank the reviewer for the positive evaluation of our manuscript, and for the constructive comments. Please find below our point-to-point response to the individual points raised.

Points to consider:
The single molecule tracking data are quite convincing. However, the data presentation could be optimized, even without additional experimental work.

Jump size is typically referred to as jump distance, maybe the authors consider rephrasing.

> We have now replaced jump size with jump distance throughout the manuscript.

The Cumulative Probability distribution (7b) is not very self-explanatory compared to typical SPT Plots (e.g. 1. Probability (y) vs jump distance (x) or 2. prob. Density (y) vs. Diffusion coefficient). These type of plots can be used to show the fitting functions.

> Both types of plots cover different aspects of the jump distances analysis. Therefore, we have now additionally provided probability density vs jump distance plots for respective pooled trajectories in Fig 7B (F_OF_1-*a*-mNG) and Appendix Fig 14A (WALP23-mNG) to show the fitting functions for each strain and condition studied. However, CDF plots have the advantage to directly monitor differences between wild type and UFA-depleted membranes, the question we are mostly interested in. We have therefore decided to keep the respective plots as well.

I am not sure whether in the analyses shown in figure 7b the jump distance is one dimensionally or is the Cumulative probability actually plotted against a squared Jump size (nm^2) and just annotated wrongly?

> The jump distance is indeed shown as a one-dimensional distance in nm and is plotted against the cumulative probability.

Even though I am generally convinced by the results displayed in Figure 7, showing the apparent diffusion as a single median (= single population, 7c) might be masking a second population arising in the fabA strain under UFA depletion conditions. Since the membrane is not expected to be homogeneously affected by the increased rigidity/decreased fluidity, not all tracks are expected to be slower compared to the WT. Instead, the range between fast and slow tracks should also increase, which could make a fit with 2 populations more suitable. According to figure S12, this is not the case, or could 33ms frames be too slow to capture very fast molecules?

> The probability density vs jump distance plot of WALP23 at 30°C (Appendix Fig S14A) indicates that the single molecule tracking setup with 33 ms frames used in this study is sufficient to detect fast diffusing signals, if available, since the lateral mobility of WALP23-mNG is higher, compared to that of F_OF_1-*a*-mNG (211 nm compared to 147nm; 30°C *fabA*(Ts) background).
> Under conditions of UFA depletion, however, the probability density vs jump distance plots revealed that the reduced mobility for both molecules, F_OF_1 as well as WALP23, resulted in a sharpened peak with a nearly homogeneous jump distance; a second population could not be detected. As mentioned above, this observation is in line with previous *in vitro* work of Ramadurai *et al* (1), showing a linear decrease of protein mobility with increasing protein concentrations in the membrane.

Why are the data shows Fig 7c as a barplot? Would a boxplot not be the more precise visualization to display the range of the cell-to-cell Dapp median values?

> We have now provided an additional analysis of the D_{app} median values of all trajectories of individual cells to show the cell-to-cell heterogeneity (Appendix Fig S13B) which coincide with the distribution of jump distance median values of individual cells, shown in Appendix Fig S13A.

Supplement to Fig. 7: how many tracks were finally utilized per condition for analysis and statistics?

> We have added Tables (Appendix Tables S2-S4) with detailed information about cell numbers and trajectory counts used for the analyses and subsequent statistics.

How does the measured diffusion coefficient (Dapp in μm2/s) compare to other values measured for membrane proteins in the literature?

In general, diffusion coefficients measured by FRAP or single molecule tracking for cytoplasmic membrane proteins of *E. coli* range from 0.01 to 0.2 $\mu m^2/s$ (3-8). The value determined in this study corresponds well to values determined for mEOS3.2-F_OF_1 (3) (see below for detailed information). We have now included information on the aspect in lines 355-360 of the manuscript.

**F_OF_1-mNG**	**0.0474 + 0.0015 $\mu m^2/s$ measured at 30 °C (this study)**
mEOS3.2-F_OF_1	0.042 ± 0.011 $\mu m^2/s$ measured at 22°C (3)
mEOS3.2-F_OF_1	0.054 ± 0.014 $\mu m^2/s$ measured at 37°C (3)

LacY-YFP	0.0265 ± 0.034 $\mu m^2/s$	(4)
MtlA-YFP	0.0283 ± 0.0037 $\mu m^2/s$	(4)
Tar-YFP	0.0171 ± 0.0019 $\mu m^2/s$	(4)
NagE-YPF	0.0196 ± 0.0024 $\mu m^2/s$	(4)
TatA-GFP	0.13 ± 0.03 $\mu m^2/s$	(5)
TatA-YFP	0.12 ± 0.05 $\mu m^2/s$	(6)
TatA-eGFP	0.026 ± 0.003 $\mu m^2/s$	(7)
GlpT-eGFP	0.153 ± 0.03 $\mu m^2/s$	(7)
MscL-eGFP	0.118 ± 0.003 $\mu m^2/s$	(7)
MscS-sfGFP	0.081 v 0.008 $\mu m^2/s$	(7)
GFP-MotB	0.008 ± 0.0013 $\mu m^2/s$	(8)

I have a general question about strain construction. Many strains used here (such as the PtsG-mNG) are expressed from plasmids. Is it confirmed that the fluorescent fusions are functional? Also, did the author check for stability of their fluorescent fusions (by western blotting)?

Few of the *E. coli* constructs including RNaseE-YFP (source publication listed in Appendix Table S6), LacZ-His (activity of LacZ verified in Fig EV1), OmpA-mCherry (source publication listed in Appendix Table S6) and WALP23-mNG/mScarlet-I (no activity by nature) are indeed expressed from plasmids. However, the majority and the key constructs including F_OF_1-*a*-mNG/mCherry (activity verified in Appendix Fig S10), FtsZ-msfGFP (source publication listed in Appendix Table S1), MreB-msfGFP (source publication listed in Appendix Table S1) and PtsG-mNG (corresponding strain selected by growth on M9-glucose minimal medium; compare lines 517-518 and 524-525 of the manuscript) are chromosomal integrations in their respective native loci. In *B. subtilis*, all of the used fusions (Hbs-GFP, GFP-FtsZ, msfGFP-MreB, and WALP23-mCherry/msfGFP) are expressed from the chromosome.
Many of the protein-FP fusions used here have been constructed earlier and tested for functionality in the respective source publications listed in Appendix Table S1.

The authors used polymyxin B to permeabilize the outer membrane of E. coli for the DPH measurements. Could polymyxin B treatment have an effect on the inner membrane?

Polymyxin B treatment would indeed have a significant effect on the inner membrane through its pore formation ability. However, we did not use Polymyxin B, but its variant Polymyxin B nonapeptide (as mentioned in Materials and Methods (lines 652-654)), which lacks the inner membrane activity and only permeabilises the outer membrane (9-12). Inner membrane disrupting full-length Polymyxin B was only used as a positive control for

membrane depolarisation and pore formation in the context of membrane barrier functions experiments (Fig. 3 and Appendix Fig S3).

In Figure EV4 the authors show the hypersensitivity of a fabA(TS) combined with a minC deletion. The authors conclude that cell division becomes growth limiting upon UFA depletion in E. coli. The argumentation is not really clear to me. A minC deletion will lead to an increase in cell length. Could this lead to an increase in sensitivity? Are longer cells in general more sensitive to fluidity changes? What happens in Bacillus if for example DivIVA is deleted and the cells get filamentous?

This is indeed a valid point although not a trivial one to experimentally test, since longer cells can only be generated by (directly or indirectly) disturbing the cell division process.
Of the two model organisms used here (*E. coli* and *B. subtilis*) only *B. subtilis* encodes *divIVA* making a comparison with *E. coli* Δ*minC* somewhat indirect. However, we did carry out the suggested experiment (see below). It turned out that *B. subtilis* Δ*divIVA* does not grow well under the growth and media conditions used to modify the *B. subtilis* fatty acid composition and fluidity. Thus, a growth defect of Δ*divIVA* itself overshadows any potential fluidity-dependent effects. For these reasons, we chose not to include this inconclusive experiment in the revised version of the manuscript.

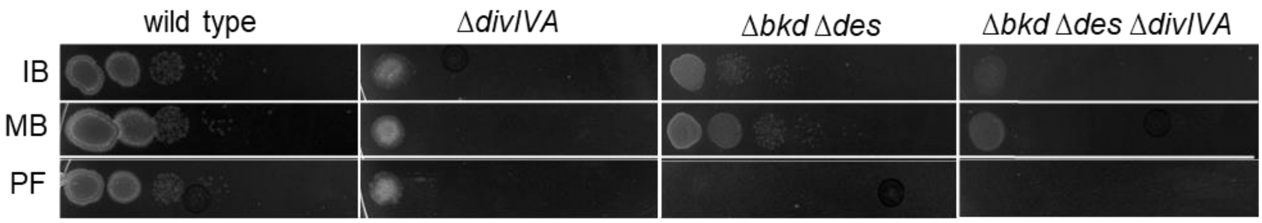

For this reason, we decided to tackle the question differently. While *fabA*(Ts) Δ*minC* combination is significantly more elongated at the permissive temperature (30°C) than *fabA*(Ts) on its own (see Fig EV4), the deletion of non-essential division genes *zapA* or *zapB* does not lead to further elongation of *fabA*(Ts) at 30°C. However, both Δ*zapA* and Δ*zapB* clearly reduce the viability upon conditions in which *fabA*(Ts) can still survive (35°C). Hence, these data argue that the fluidity-sensitivity of *E. coli* division mutants is not simply caused by oversensitivity of longer cells. We have now added this new data in support of our conclusions as a new Appendix Fig S5, mentioned in the manuscript text at lines 251-253.

Referee #3:

In their manuscript entitled 'Low membrane fluidity triggers lipid phase separation and protein segregation in vivo', Gohrbandt and coworkers study the effect of decreasing membrane fluidity (by manipulating membrane lipid composition) in E. coli and B. subtilis. They set up systems to acutely deplete branched chain fatty acids and unsaturated fatty acids from B. subtilis and E. coli lipids respectively which induces a reduction in their membrane fluidity. Following these treatments, they observe membrane depolarization, disturbed localization of membrane-associated cellular factors, membrane phase separation with consequent segregation of transmembrane proteins in the formed domains. The manuscript is well written, the data are of good quality, and the authors' interpretation of the data is justified by the evidence provided. The central question about the cellular consequences of inadequate membrane fluidity is important and the data obtained by the authors are an important advancement towards addressing this issue.

We would like to thank the reviewer for the positive evaluation of our manuscript, and for the constructive comments.

Nonetheless, the observed phenotypes are obtained by changing membrane fluidity by an extent way bigger than that dealt by the cells when reacting to temperature changes during homeoviscous adaptation. Thus, as the authors state "while both E. coli and B. subtilis adapt their membrane composition and fluidity even upon subtle changes in temperature, the failure to do so is not associated with immediate growth-inhibitory consequences". This evidence triggers the question of what selective pressure has operated on these organisms to shape a metabolic rewiring aimed at adjusting subtle changes in membrane fluidity.

In my opinion, the authors should provide at least a perspective explanation for this by subjecting bacteria with mildly affected membrane fluidity to challenges they might encounter in their natural environment (phage intoxication, interaction with antibacterial peptides or antibiotics) and by looking at their capability to adapt to their environment (i.e., to transition from a motile planktonic cell state to a sessile biofilm state).

> We fully agree with the reviewer. This is indeed the “big question” emerging from our study, and also one we are actively pursuing. The Strahl-lab has a PhD student (started last year) working on the very question, whether bacteria with miss-regulated membrane fluidity show sensitivity towards adverse environmental conditions (our focus is on osmolarity and pH) and antimicrobial peptides (focus on cationic antimicrobial peptides, especially LL-37). In addition, we are in the process of writing another full paper that argues for regulation of membrane thickness (rather than regulation of membrane fluidity) as a potential reason for lipid adaptation in response to a changing environment. We feel that attempting to answer these important questions with relatively superficial additional experiments would not be very constructive. Rather, these questions are better answered more comprehensively in dedicated manuscripts. However, we have now included further discussion dealing with this very relevant question (lines 410-423).

References:

1: Ramadurai S, Holt A, Krasnikov V, van den Bogaart G, Killian JA, Poolman B (2009) Lateral diffusion of membrane proteins. *J Am Chem Soc* 131:12650–12656.

2: Ramadurai S, Duurkens R, Krasnikov VV, Poolman B (2010) Lateral diffusion of membrane proteins: Consequences of hydrophobic mismatch and lipid composition. *Biophys J* 99:1482-1489.

3: Renz A, Renz M, Klütsch D, Deckers-Hebestreit G, Börsch M (2015) 3D-localisation microscopy and tracking of F_OF_1-ATP synthases in living bacteria. *Proc SPIE* 9331:93310D.

4: Kumar M, Mommer MS, Sourjik V (2010) Mobility of cytoplasmic, membrane, and DNA-binding proteins in *Escherichia coli*. *Biophys J* 98:552-559.

5: Mullineaux CW, Nenninger A, Ray N, Robinson C (2006) Diffusion of green fluorescent protein in three cell environments in *Escherichia coli*. *J Bacteriol* 188:3442-3448.

6: Leake MC, Greene NP, Godun RM, Granjon T, Buchanan G, Chen S, Berry RM, Palmer T, Berks BC (2008) Variable stoichiometry of the TatA component of the twin-arginine protein transport system observed by *in vivo* single-molecule imaging. *Proc Natl Acad Sci USA* 105:15376-15381.

7: Oswald F, Varadarajan A, Lill H, Peterman EJG, Bollen YEM (2016) MreB-dependent organization of

the *E. coli* cytoplasmic membrane controls membrane protein diffusion. Biophys J 110:1139-1149.

8: Leake MC, Chandler JH, Wadhams GH, Bai F, Berry RM, Armitage JP (2006) Stoichiometry and turnover in single, functioning membrane protein complexes. *Nature* 443:355-358.

9: Vaara M, Vaara T (1983) Polycations sensitize enteric bacteria to antibiotics. *Antimicrob Agents Chemother* 24:107-113.

10: Vaara M (1992) Agents that increase the permeability of the outer membrane. *Microbiol Rev* 56:395-411.

11: Daugelavicius R, Bakiene E, Bamford DH (2000). Stages of polymyxin B interaction with the Escherichia coli cell envelope. *Antimicrob Agents Chemother* 44:2969-78.

12: Lu S, Walters G, Parg R, Dutcher JR (2014). Nanomechanical response of bacterial cells to cationic antimicrobial peptides. *Soft Matter* 10:1806-15.

1st Revision - Editorial Decision 15th Dec 2021

15th Dec 2021

Re: EMBOJ-2021-109800R
Low membrane fluidity triggers lipid phase separation and protein segregation in vivo

Dear Dr. Strahl,

Thank you for submitting your revised manuscript. We have now received comments from referee #2 (please see below) and I am pleased to say that she/she now also supports publication. Therefore, I would ask you to address a number of editorial issues that are listed in detail below in a final revised version of the manuscript.

In addition, before publication, we always also again look at title, abstract and synopsis to ensure that the findings of the study are optimally highlighted, also to a more generalist audience. I will attach a separate document (EMBOJ-2021-109800 productionFiles_auth.docx), with suggested changes and would like to ask you to review this and either add your edits or approve this version. Please upload this file when submitting the revised manuscript as a related manuscript file. Alternatively, you can also send this to me by email.

Please feel free to contact me if you have further questions regarding this final revision or any of the specific points listed below. Thank you again for giving us the chance to consider your manuscript for The EMBO Journal.

Kind regards,

Stefanie

Stefanie Boehm
Editor
The EMBO Journal

Referee #2:

The authors present a revised version of their manuscript "Low membrane fluidity triggers lipid phase separation and protein segregation in vivo". They have adequately addressed all points raised in the reviews. I have no further comments and congratulate all authors to this really insightful paper!

The authors have made all requested editorial changes.

21st Dec 2021

Re: EMBOJ-2021-109800R1
Low membrane fluidity triggers lipid phase separation and protein segregation in living bacteria

Dear Dr. Strahl,

Thank you again for submitting the final revised version of your manuscript and addressing the remaining points. I am pleased to inform you that we have now accepted it for publication in The EMBO Journal.

Your article will be processed for publication in The EMBO Journal by EMBO Press and Wiley, who will contact you with further information regarding production/publication procedures and license requirements.

Should you be planning a Press Release on your article, please get in contact with embojournal@wiley.com as early as possible, in order to coordinate publication and release dates.

Please also be aware that there are country specific agreements with our publisher Wiley, through which you may be eligible for free publication of your article in the open access format (https://authorservices.wiley.com/author-resources/Journal-Authors/open-access/affiliation-policies-payments/jisc-agreement.html). Please contact either the administration at your institution or our publishers at Wiley (embojournal@wiley.com) for further questions.

Congratulations on your successful publication, and thank you again for this contribution to The EMBO Journal! Please continue to consider EMBO Journal for your work in the future.

Kind regards,

Stefanie

Stefanie Boehm
Editor
The EMBO Journal

EMBO PRESS

YOU MUST COMPLETE ALL CELLS WITH A PINK BACKGROUND ↓

PLEASE NOTE THAT THIS CHECKLIST WILL BE PUBLISHED ALONGSIDE YOUR PAPER

Corresponding Author Name: Henrik Strahl
Journal Submitted to: EMBO Journal
Manuscript Number: EMBOJ-2021-109800

Reporting Checklist For Life Sciences Articles (Rev. June 2017)

This checklist is used to ensure good reporting standards and to improve the reproducibility of published results. These guidelines are consistent with the Principles and Guidelines for Reporting Preclinical Research issued by the NIH in 2014. Please follow the journal's authorship guidelines in preparing your manuscript.

A- Figures
1. Data
The data shown in figures should satisfy the following conditions:

- the data were obtained and processed according to the field's best practice and are presented to reflect the results of the experiments in an accurate and unbiased manner.
- figure panels include only data points, measurements or observations that can be compared to each other in a scientifically meaningful way.
- graphs include clearly labeled error bars for independent experiments and sample sizes. Unless justified, error bars should not be shown for technical replicates.
- if n< 5, the individual data points from each experiment should be plotted and any statistical test employed should be justified
- Source Data should be included to report the data underlying graphs. Please follow the guidelines set out in the author ship guidelines on Data Presentation.

2. Captions

Each figure caption should contain the following information, for each panel where they are relevant:

- a specification of the experimental system investigated (eg cell line, species name).
- the assay(s) and method(s) used to carry out the reported observations and measurements
- an explicit mention of the biological and chemical entity(ies) that are being measured.
- an explicit mention of the biological and chemical entity(ies) that are altered/varied/perturbed in a controlled manner.

- the exact sample size (n) for each experimental group/condition, given as a number, not a range;
- a description of the sample collection allowing the reader to understand whether the samples represent technical or biological replicates (including how many animals, litters, cultures, etc.).
- a statement of how many times the experiment shown was independently replicated in the laboratory.
- definitions of statistical methods and measures:
  - common tests, such as t-test (please specify whether paired vs. unpaired), simple χ2 tests, Wilcoxon and Mann-Whitney tests, can be unambiguously identified by name only, but more complex techniques should be described in the methods section;
  - are tests one-sided or two-sided?
  - are there adjustments for multiple comparisons?
  - exact statistical test results, e.g., P values = x but not P values < x;
  - definition of 'center values' as median or average;
  - definition of error bars as s.d. or s.e.m.

Any descriptions too long for the figure legend should be included in the methods section and/or with the source data.

In the pink boxes below, please ensure that the answers to the following questions are reported in the manuscript itself. Every question should be answered. If the question is not relevant to your research, please write NA (non applicable). We encourage you to include a specific subsection in the methods section for statistics, reagents, animal models and human subjects.

USEFUL LINKS FOR COMPLETING THIS FORM

http://www.antibodypedia.com
http://1degreebio.org
http://www.equator-network.org/reporting-guidelines/improving-bioscience-research-repor
http://grants.nih.gov/grants/olaw/olaw.htm
http://www.mrc.ac.uk/Ourresearch/Ethicsresearchguidance/Useofanimals/index.htm
http://ClinicalTrials.gov
http://www.consort-statement.org
http://www.consort-statement.org/checklists/view/32-consort/66-title
http://www.equator-network.org/reporting-guidelines/reporting-recommendations-for-tumo
http://datadryad.org
http://figshare.com
http://www.ncbi.nlm.nih.gov/gap
http://www.ebi.ac.uk/ega
http://biomodels.net/
http://biomodels.net/miriam/
http://jjj.biochem.sun.ac.za
https://osp.od.nih.gov/biosafety-biosecurity-and-emerging-biotechnology/
http://www.selectagents.gov/

B- Statistics and general methods

Please fill out these boxes ↓ (Do not worry if you cannot see all your text once you press return)

1.a. How was the sample size chosen to ensure adequate power to detect a pre-specified effect size?	Sample size was chosen based on previous experience with similar type of experiments, and based on common good practise on the specific field.
1.b. For animal studies, include a statement about sample size estimate even if no statistical methods were used.	N/A
2. Describe inclusion/exclusion criteria if samples or animals were excluded from the analysis. Were the criteria pre-established?	No samples were excluded.
3. Were any steps taken to minimize the effects of subjective bias when allocating animals/samples to treatment (e.g. randomization procedure)? If yes, please describe.	Bias upon quantitative microscopy was mitigated by choosing analysed cells based on phase contrast images (thus obscuring the fluorescence signal), by analysing every cell on a given image field of view if applicable, and using automated cell detection and analysis algorithms and macros.
For animal studies, include a statement about randomization even if no randomization was used.	N/A
4.a. Were any steps taken to minimize the effects of subjective bias during group allocation or/and when assessing results (e.g. blinding of the investigator)? If yes please describe.	Bias upon quantitative microscopy was mitigated by choosing analysed cells based on phase contrast images (thus obscuring the fluorescence signal), by analysing every cell on a given image field of view if applicable, and using automated cell detection and analysis algorithms and macros.
4.b. For animal studies, include a statement about blinding even if no blinding was done	N/A
5. For every figure, are statistical tests justified as appropriate?	Yes
Do the data meet the assumptions of the tests (e.g., normal distribution)? Describe any methods used to assess it.	Yes, as assesed by visual inspection of the distributions and prior knowledge of the type of data.
Is there an estimate of variation within each group of data?	Yes

Is the variance similar between the groups that are being statistically compared?	Yes

C- Reagents

6. To show that antibodies were profiled for use in the system under study (assay and species), provide a citation, catalog number and/or clone number, supplementary information or reference to an antibody validation profile. e.g., Antibodypedia (see link list at top right), 1DegreeBio (see link list at top right).	The antibodies used are monoclonal mouse antibodies specific for FO-a (GDH 14-5C6 (Jäger et al, 1998)) mNeonGreen (32F6, ChromoTek), and secondary IRDye™800DX-labelled goat-anti-mouse IgG (H+L) (LI-COR Biosciences)
7. Identify the source of cell lines and report if they were recently authenticated (e.g., by STR profiling) and tested for mycoplasma contamination.	N/A

* for all hyperlinks, please see the table at the top right of the document

D- Animal Models

8. Report species, strain, gender, age of animals and genetic modification status where applicable. Please detail housing and husbandry conditions and the source of animals.	N/A
9. For experiments involving live vertebrates, include a statement of compliance with ethical regulations and identify the committee(s) approving the experiments.	N/A
10. We recommend consulting the ARRIVE guidelines (see link list at top right) (PLoS Biol. 8(6), e1000412, 2010) to ensure that other relevant aspects of animal studies are adequately reported. See author guidelines, under 'Reporting Guidelines'. See also: NIH (see link list at top right) and MRC (see link list at top right) recommendations. Please confirm compliance.	N/A

E- Human Subjects

11. Identify the committee(s) approving the study protocol.	N/A
12. Include a statement confirming that informed consent was obtained from all subjects and that the experiments conformed to the principles set out in the WMA Declaration of Helsinki and the Department of Health and Human Services Belmont Report.	N/A
13. For publication of patient photos, include a statement confirming that consent to publish was obtained.	N/A
14. Report any restrictions on the availability (and/or on the use) of human data or samples.	N/A
15. Report the clinical trial registration number (at ClinicalTrials.gov or equivalent), where applicable.	N/A
16. For phase II and III randomized controlled trials, please refer to the CONSORT flow diagram (see link list at top right) and submit the CONSORT checklist (see link list at top right) with your submission. See author guidelines, under 'Reporting Guidelines'. Please confirm you have submitted this list.	N/A
17. For tumor marker prognostic studies, we recommend that you follow the REMARK reporting guidelines (see link list at top right). See author guidelines, under 'Reporting Guidelines'. Please confirm you have followed these guidelines.	N/A

F- Data Accessibility

18: Provide a "Data Availability" section at the end of the Materials & Methods, listing the accession codes for data generated in this study and deposited in a public database (e.g. RNA-Seq data: Gene Expression Omnibus GSE39462, Proteomics data: PRIDE PXD000208 etc.) Please refer to our author guidelines for 'Data Deposition'.<br><br>Data deposition in a public repository is mandatory for:<br>a. Protein, DNA and RNA sequences<br>b. Macromolecular structures<br>c. Crystallographic data for small molecules<br>d. Functional genomics data<br>e. Proteomics and molecular interactions	Data availability section is included.
19. Deposition is strongly recommended for any datasets that are central and integral to the study; please consider the journal's data policy. If no structured public repository exists for a given data type, we encourage the provision of datasets in the manuscript as a Supplementary Document (see author guidelines under 'Expanded View' or in unstructured repositories such as Dryad (see link list at top right) or Figshare (see link list at top right).	Numerical source data for all graphs and diagrams is provided with this manuscript.
20. Access to human clinical and genomic datasets should be provided with as few restrictions as possible while respecting ethical obligations to the patients and relevant medical and legal issues. If practically possible and compatible with the individual consent agreement used in the study, such data should be deposited in one of the major public access-controlled repositories such as dbGAP (see link list at top right) or EGA (see link list at top right).	N/A
21. Computational models that are central and integral to a study should be shared without restrictions and provided in a machine-readable form. The relevant accession numbers or links should be provided. When possible, standardized format (SBML, CellML) should be used instead of scripts (e.g. MATLAB). Authors are strongly encouraged to follow the MIRIAM guidelines (see link list at top right) and deposit their model in a public database such as Biomodels (see link list at top right) or JWS Online (see link list at top right). If computer source code is provided with the paper, it should be deposited in a public repository or included in supplementary information.	N/A

G- Dual use research of concern

22. Could your study fall under dual use research restrictions? Please check biosecurity documents (see link list at top right) and list of select agents and toxins (APHIS/CDC) (see link list at top right). According to our biosecurity guidelines, provide a statement only if it could.	N/A
